# Cyclopentannulated Decacyclenes as Carbon‐Based Multistage Electron Acceptors

**DOI:** 10.1002/asia.202500551

**Published:** 2025-06-10

**Authors:** Silas C. Eiden, Erik Misselwitz, Frank Rominger, Milan Kivala

**Affiliations:** ^1^ Organisch‐Chemisches Institut Universität Heidelberg Im Neuenheimer Feld 270 69120 Heidelberg Germany

**Keywords:** Aromaticity, Cyclopentannulation, Decacyclene, Non‐benzenoid, Redox chemistry

## Abstract

We report a series of cyclopentannulated decacyclenes that act as remarkable multistage electron acceptors. The compounds were synthesized in a modular approach via Yamamoto cyclotrimerization of brominated pyracylene precursors, allowing the introduction of electron‐withdrawing, ‐donating, and solubilizing fluoro, methoxy, and *tert*‐butyl moieties, respectively. The subsequent π‐expansion of the polycyclic scaffold was achieved by oxidative cyclodehydrogenation. X‐ray crystallographic analysis revealed the propeller‐shaped geometry of the decacyclene core with a pronounced bond length alternation in the central six‐membered ring and the columnar packing motif in the solid state. Depending on the substitution pattern, the compounds are capable of up to six reversible reductions within a particularly narrow potential range between −1.45 and −2.86 V under electrochemical conditions. This result is in stark contrast to only two reductions of parent decacyclene and clearly demonstrates the major impact of strategic cyclopentannulation on the redox properties of the sp^2^‐carbon scaffold, which was further supported by density functional theory studies.

## Graphical abstract

A series of cyclopentannulated decacyclenes were synthesized in a modular approach. Subsequent cyclization allowed the expansion of the π‐system. The carbon‐based compounds undergo up to six reversible reductions within an impressively narrow potential range between −1.45 and −2.86 V under electrochemical conditions.

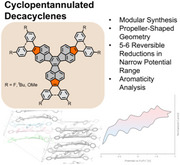

## 1 Introduction

**Figure 1:**
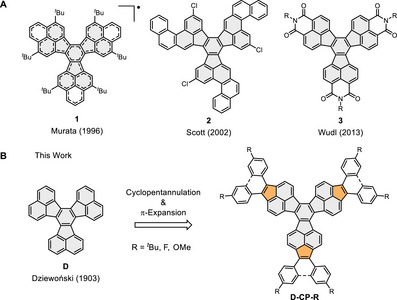
A) Selected examples of decacyclene (**D**) derivatives reported in the literature.^[^ ^17^, ^18^, ^19^ ^]^ B) Cyclopentannulation and π‐expansion of **D** toward **D‐Cp‐*^t^* Bu**, **D‐Cp‐F**, **D‐Cp‐OMe**, and **D‐Cp‐*^t^* Bu‐Cyc** presented in this work.

Polycyclic aromatic hydrocarbons (PAHs) with electron‐accepting properties are crucial for the development of functional optoelectronic materials for various applications.^[^ ^1^ ^]^ A common strategy to obtain such materials is the functionalization of intrinsically electron‐donating π‐conjugated scaffolds with suitable electron‐withdrawing moieties, such as fluoro,^[^ ^2^ ^]^ cyano,^[^ ^3^ ^]^ and imide groups.^[^ ^4^ ^]^ However, the inherent properties of these functionalities, including their reactivity toward nucleophiles and often low solubility, impose limitations on this approach.^[^ ^5^ ^]^ Conversely, the incorporation of non‐benzenoid rings into the carbon‐sp^2^ scaffolds has been demonstrated as a potent strategy to achieve electron‐deficient PAHs. In particular, π‐conjugated five‐membered rings are highly relevant in this context due to their propensity for electron uptake driven by the formation of a formally Hückel aromatic 6π‐electron system.^[^ ^6^, ^7^ ^]^ The resulting cyclopentannulated PAHs (CP‐PAHs) possess unique geometries, electronic structures, and a high electron affinity.^[^ ^8^ ^]^ A prominent CP‐PAH is decacyclene (**D**), which was first reported independently by Dziewoński and Rehländer more than 100 years ago.^[^ ^9^ ^]^ Due to its geometry, optoelectronic and redox properties, **D** and its derivatives have since found a variety of applications, including organometallic chemistry,^[^ ^10^ ^]^ surface modification,^[^ ^11^ ^]^ supramolecular,^[^ ^12^ ^]^ and ferromagnetic materials,^[^ ^13^ ^]^ dopant emitters in organic light emitting diodes (OLEDs),^[^ ^14^ ^]^ and precursors for chiral aromatic structures^[^ ^15^ ^]^ and carbon nanotubes.^[^ ^16^ ^]^ In addition, the π‐expansion of **D** toward phenalenyl moieties yielded the open‐shell scaffold **1** with amphoteric redox properties capable of three reductions and three oxidations under electrochemical conditions (Figure 1).^[^ ^17^ ^]^ The strategic π‐expansion and chlorination in **2** allowed the conversion of this compound to C_60_ upon flash vacuum pyrolysis.^[^ ^18^ ^]^ Decoration of **D** with *N*‐alkylimide functionalities afforded three‐fold symmetric **3**, which formed self‐assembled columnar aggregates used as acceptors in organic photovoltaics.^[^ ^19^ ^]^

Peripheral cyclopentannulation of PAHs has been shown to often result in superior electron‐accepting properties compared to the internally fused counterparts.^[^ ^20^, ^21^ ^]^ Notable examples are the inherently electron‐rich acenes that were successfully transformed into electron acceptors through peripheral cyclopentannulation.^[^ ^20^, ^22^ ^]^

Herein, we report the concise synthesis of a series of cyclopentannulated decacyclenes (**D‐Cp**) via intermolecular cyclotrimerization of dibrominated pyracylene precursors under Ni‐mediated Yamamoto conditions. Our synthetic strategy allows the incorporation of electron‐withdrawing (fluoro in **D‐Cp‐F**), electron‐donating (methoxy in **D‐Cp‐OMe**), and solubilizing (*tert*‐butyl in **D‐Cp‐*^t^* Bu**) groups as well as post‐functionalization to π‐expanded **D‐Cp‐*^t^* Bu‐Cyc**. The resulting polycyclic scaffolds are exceptional electron acceptors capable of up to six reversible reductions under electrochemical conditions, making them the most electron‐accepting decacyclene derivatives reported to date. In addition, we have elucidated the effect of cyclopentannulation and functionalization on the structural and optoelectronic properties both in experiment and in theory.

## 2 Results and Discussion

**Scheme 1:**
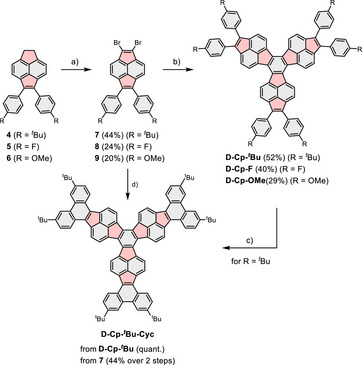
Synthesis of cyclopentannulated decacyclenes **D‐Cp‐** ^*t*^ **Bu**, **D‐Cp‐F**, **D‐Cp‐OMe** and **D‐Cp‐** ^*t*^ **Bu‐Cyc**. Reagents and conditions: a) NBS, AIBN, MeCN, **7**: 80 °C, 18 h **8**: 100 °C, 18 h, **9**: 60 °C, 6 h; b) [Ni(cod)_2_], cod, 2,2′bipy, 1,4‐dioxane, 120 °C, 18 h; c) DDQ, TfOH, CH_2_Cl_2_, 0 °C, 20 min; d) first c) then b). NBS = *N*‐bromosuccinimide, AIBN = azobisisobutyronitrile, cod = 1,5‐cyclooctadiene, 2,2′bipy = 2,2′‐bipyridine, DDQ = 2,3‐dichloro‐5,6‐dicyano‐1,4‐benzoquinone, TfOH = trifluoromethanesulfonic acid.

The arylated dihydropyracylene precursors **4**–**6** were prepared from 5,6‐dibromoacenphthene and the corresponding tolanes according to literature (for synthetic details, see the Supporting Information).^[^ ^23^ ^]^ The subsequent radical bromination followed by elimination afforded the dibrominated pyracylenes **7**–**9** in moderate yields (Scheme 1).

As shown by X‐ray crystallography for **7** and **8** (see the Supporting Information), the resulting compounds feature a pyracylene core, a subunit of buckminster fullerene C_60_, which has been used in the development of electron‐accepting PAHs.^[^ ^6^, ^24^ ^]^ Hence, it is worth noting that our synthetic route establishes a modular access to designer building blocks suitable for the incorporation of electron‐deficient pyracylene moieties into larger PAHs.

**Figure 2:**
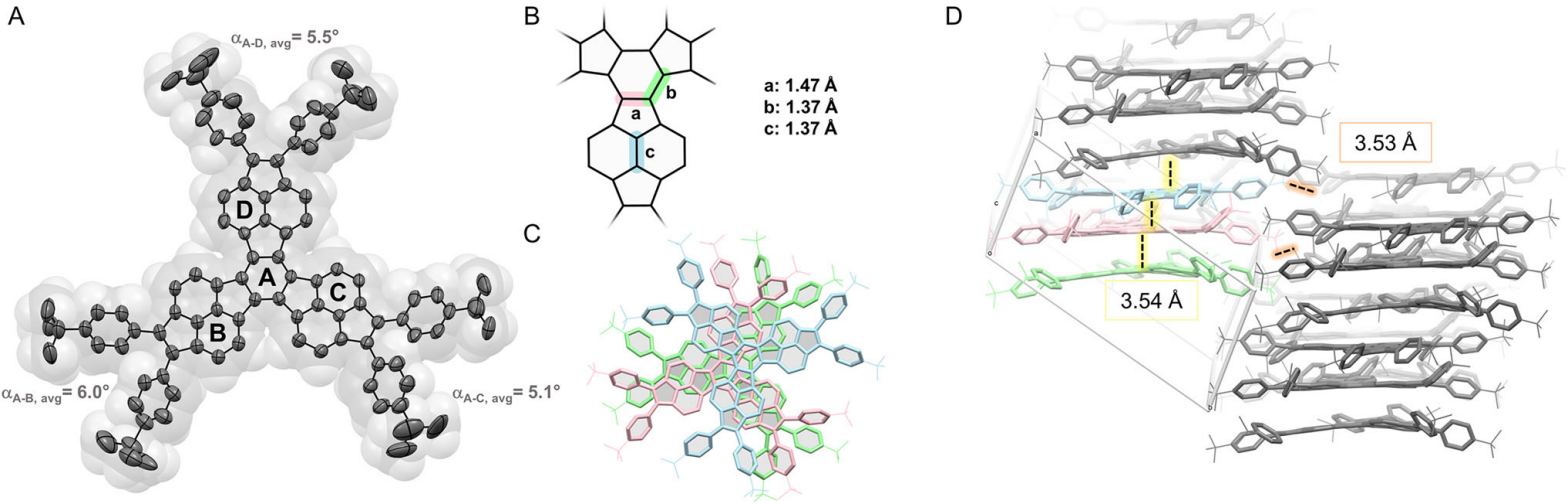
A) X‐ray crystallographic structure of **D‐Cp‐** *^t^* **Bu** with average angles α between the ring planes defined by central six‐membered ring **A** and the naphthalene units containing rings **B**, **C,** and **D**, respectively. B) Characteristic averaged bond lengths in **D‐Cp‐** *^t^* **Bu**. With respect to the five‐membered ring, the bonds **a** and **b** are denoted as endocyclic and exocyclic, respectively. C) Top view of the supramolecular arrangement within the unit cell. The independent molecules are shown in different colors. D) Side view of the packing motif in the solid state. Distances are calculated upon mean planes defined by all carbons of the cyclopentannulated decacyclene core (yellow) or between carbon atoms on the neighboring stacks (orange). Ellipsoids at 50% probability level; hydrogen atoms and solvent molecules omitted for clarity.

Subsequently, nickel‐mediated Yamamoto cyclotrimerization^[^ ^25^ ^]^ of the brominated building blocks **7**–**9** yielded the corresponding decacyclenes **D‐Cp‐*^t^* Bu**, **D‐Cp‐F**, and **D‐Cp‐OMe** in moderate yields between 29% and 52%. The formation of higher cyclooligomers was not observed under these conditions. Oxidative cyclodehydrogenation of **D‐Cp‐*^t^* Bu** with 2,3‐dichloro‐5,6‐dicyano‐1,4‐benzoquinone (DDQ) and trifluoromethanesulfonic acid (TfOH) afforded three‐fold cyclized **D‐Cp‐*^t^* Bu‐Cyc** in quantitative yield. Alternatively, **D‐Cp‐*^t^* Bu‐Cyc** was obtained in an overall yield of 44% over two steps from **7** via oxidative cyclodehydrogenation under the same conditions followed by Yamamoto cyclotrimerization. All target decacyclenes are brown solids that are stable in air under ambient conditions and are sparingly soluble in common organic solvents. The identity of all compounds was unambiguously confirmed by common spectroscopic techniques, revealing the expected three‐fold symmetry of the polycyclic scaffolds (see the Supporting Information). Single crystals of **D‐Cp‐*^t^* Bu** suitable for X‐ray crystallographic analysis were grown by slow gas phase diffusion of methanol into a solution of the compound in THF at room temperature (Figure 2). The compound crystallizes in the triclinic space group *P*1 with three independent molecules in the unit cell, and thus the averaged values calculated upon consideration of all independent molecules were used in the following discussion of the structural features. The cyclopentannulated decacyclene core exhibits a slightly twisted propeller‐like conformation due to intramolecular repulsion between the adjacent C(sp^2^)–H atoms. Similar to parent decacyclene,^[^ ^26^ ^]^ the overall twist is modest, with angles α between the ring planes determined by the central six‐membered ring and the naphthalene units of 6.0°–5.1° (between 9.3° and 7.8° for **D**, for details, see the Supporting Information). The lateral phenyls are twisted out of the plane defined by the π‐expanded decacyclene core with torsion angles ranging from 35.0° to 55.5°. In contrast, the bond length alternation within the central benzenoid ring is significantly increased in **D‐Cp‐*^t^* Bu** compared to parent **D**. Thus, the average length of the endocyclic bonds **a** (Figure 2) fused to the five‐membered rings is 1.47 Å, while the exocyclic bonds **b** are significantly shortened to 1.37 Å (for parent **D**, endocyclic bonds 1.44 Å, exocyclic bonds 1.38 Å).^[^ ^26^ ^]^ Notably, the central bond in the naphthalene subunits **c** of **D‐Cp‐*^t^* Bu** is shortened to 1.37 Å (in **D** 1.40 Å), which is comparable to pyracylene with 1.36 Å.^[^ ^24b^ ^]^ In the solid state, the compound forms supramolecular twisted stacks upon π–π stacking with a distance of 3.54 Å.

**Figure 3:**
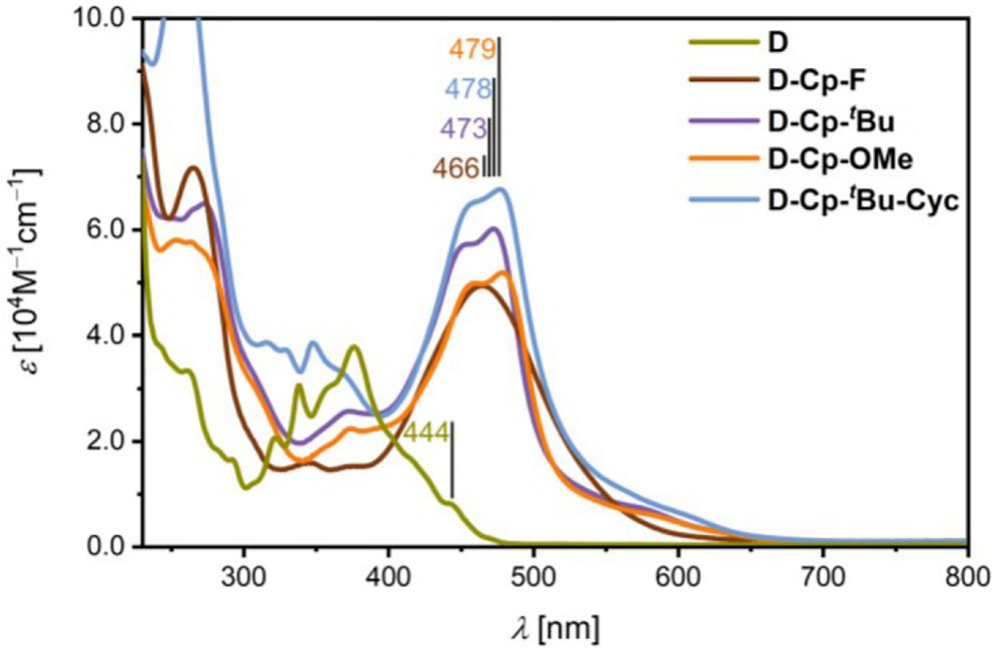
UV/Vis absorption spectra of **D**, **D‐Cp‐F**, **D‐Cp‐** *^t^* **Bu**, **D‐Cp‐OMe**, and **D‐Cp‐** *^t^* **Bu‐Cyc** recorded in CH_2_Cl_2_ at room temperature.

The optoelectronic properties of the compounds were examined via UV/Vis absorption spectroscopy in CH_2_Cl_2_ at room temperature (Figure 3 and Table 1). Consistent with observations reported for related cyclopentannulated PAHs, none of the decacyclene derivatives display photoluminescence,^[^ ^27^ ^]^ in contrast to pristine decacyclene.^[^ ^14^ ^]^ Cyclopentannulation redshifts the longest wavelength absorption maximum (*λ*_max_) by 29 nm from 444 nm (for **D**) to 473 nm (for **D**‐**Cp‐*^t^* Bu**). Functionalization in the periphery has only a moderate influence on the absorption properties, with a slight redshift of *λ*_max_ when going from fluorinated **D‐Cp‐F** (466 nm) and *tert‐*butyl‐substituted **D‐Cp‐*^t^* Bu** (473 nm) to **D‐Cp‐OMe** (479 nm) with the methoxy groups. The cyclization of **D‐Cp‐*^t^* Bu** to **D‐Cp‐*^t^* Bu‐Cyc** results in a negligible redshift of 5 nm to 478 nm. The observed spectral broadening, particularly for **D‐Cp‐F**, is attributed to aggregation. This is supported by the dilution experiments in CH_2_Cl_2_ at rt, showing a redshift of *λ*_max_ upon dilution for all species (see the Supporting Information). Lambert–Beer behavior is observed at concentrations around 10^−6^ m for **D‐Cp‐^*t*^Bu** and **D‐Cp‐OMe** and at 10^−7^ m for **D‐Cp‐^*t*^Bu‐Cyc**. In contrast, **D‐Cp‐F** exhibits persistent spectral broadening in CH_2_Cl_2_ even at concentrations as low as 1.44  ×  10^−7^ m, whereas in toluene, absorption features comparable to those of the other derivatives emerge at concentrations around 10^−8^ m. To corroborate the experimental findings, TD‐DFT calculations at the CAM‐B3LYP^[^ ^28^ ^]^(D3BJ)^[^ ^29^ ^]^/6–311G(d,p)^[^ ^30^ ^]^ level of theory were conducted (for computational details, see the Supporting Information). The calculations support the observed trends and indicate that the most significant contribution to the absorption spectrum originates from the HOMO–1/HOMO→LUMO+2 transitions, which are mainly located at the cyclopentannulated decacyclene core for all compounds. The HOMO→LUMO (S_1_) transitions possess very low oscillator strengths in all systems, which is consistent with the observed lack of photoluminescence.

**Table 1:** Experimental photophysical and electrochemical data of **D‐Cp‐** *^t^* **Bu**, **D‐Cp‐OMe**, **D‐Cp‐F,** and **D‐Cp‐** *^t^* **Bu‐Cyc** and calculated FMOs energies.

Compound	λ_max_/λ_on_ (nm)^a)^, ^b)^ ^)^	*E*_g_^opt^ (eV)^c^ ^)^	*E*_red,1_ (V)^d^ ^)^	*E*_red,2_ (V)	*E*_red,3_ (V)	*E*_red,4_ (V)	*E*_red,5_ (V)	*E*_red,6_ (V)	*E*_HOMO,DFT_/*E*_LUMO,DFT_ (eV)^e^ ^)^	*E*_LUMO,SWV_ (eV)^f^ ^)^
**D‐Cp‐** *^t^* **Bu**	473/653	1.90	−1.50	−1.70	−1.97	−2.29	−2.58		−5.23/−2.73	−3.60
**D‐Cp‐** *^t^* **Bu‐Cyc**	478/664	1.87	−1.56	−1.70	−1.90	−2.26	−2.57	−2.86	−5.25/−2.76	−3.54
**D‐Cp‐OMe**	479/660	1.88	−1.52	−1.74	−2.00	−2.34	−2.62		−5.04/−2.63	−3.58
**D‐Cp‐F**	466/645	1.92	−1.45	−1.66	−1.82	−2.25	−2.51	−2.77	−5.57/−3.06	−3.65

^a)^ Recorded in CH_2_Cl_2_ at room temperature.

^b)^ Estimated using the tangent method.

^c)^ Optical band gap *E*_g_^opt^ = hc/λ_on_.

^d)^ All electrochemical potentials extracted from SWV measurement in THF at room temperature.

^e)^ DFT calculated at B3LYP(D3BJ)/6–311G(d,p) level of theory.

^f)^ Calculated according to *E*_LUMO,SWV_ = −(*E*_red,1_ + 5.10 ) eV; oxidation potential of Fc against vacuum is set at 5.10 eV.^[^ ^36^ ^]^

**Figure 4:**
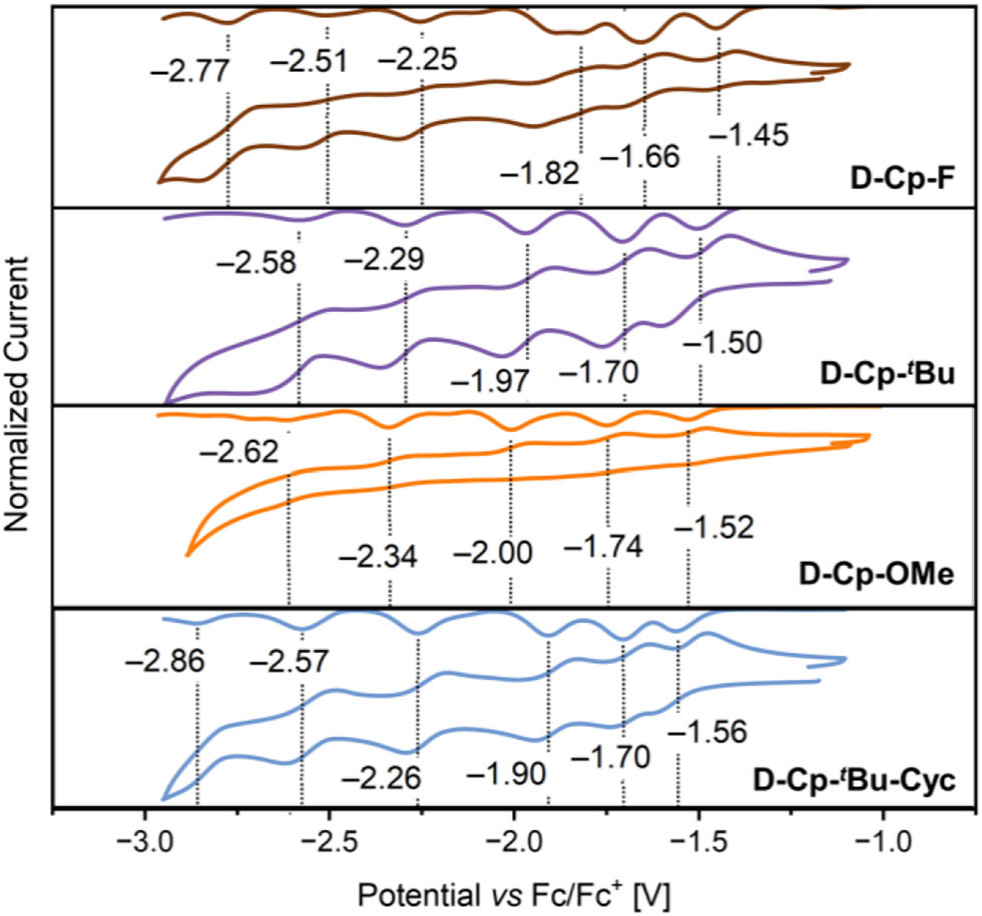
CV and SWV data in the negative potential range of **D‐Cp‐F**, **D‐Cp‐** *^t^* **Bu**, **D‐Cp‐OMe**, and **D‐Cp‐** *^t^* **Bu‐Cyc** versus Fc/Fc^+^ in THF at room temperature (2 mM, scan rate 149 mV s^−1^, *n*‐Bu_4_NPF_6_ as supporting electrolyte).

The electrochemical properties of the cyclopentannulated decacyclenes were investigated by cyclic voltammetry (CV), differential pulse voltammetry (DPV), and square wave voltammetry (SWV) in THF at room temperature (Figure 4, Table 1 and the Supporting Information). Although the oxidations are poorly defined and irreversible for all compounds, the reduction steps show perfect reversibility. Overall, five (**D‐Cp‐** *^t^* **Bu** and **D‐Cp‐OMe**) to six (**D‐Cp‐F** and **D‐Cp‐** *^t^* **Bu‐Cyc**) reduction events are found within the accessible potential window of THF. Notably, pristine decacyclene **D** exhibits only three reversible reductions under analogous conditions (see the Supporting Information). The electrochemistry of decacylene diimides **3** is poorly resolved and shows only two reduction events.^[^ ^19a^ ^]^ The first reduction of all cyclopentannulated decacyclenes occur anodically shifted compared to pristine **D**, which is reduced to the corresponding radical anion at −1.99 V. The largest anodic shift of 540 mV is observed for the fluorinated **D‐Cp‐F** with the first reduction at −1.45 V, followed by **D‐Cp‐** *^t^* **Bu** and **D‐Cp‐OMe** reduced at −1.50 and −1.52 V, respectively. Expansion of the π‐system from **D‐Cp‐** *^t^* **Bu** to **D‐Cp‐** *^t^* **Bu‐Cyc** results in a cathodic shift of 60 mV of the first reduction from −1.50 V to −1.56 V and the occurrence of an additional reduction event for the latter. Furthermore, the six reduction events of **D‐Cp‐** *^t^* **Bu‐Cyc** occur within a remarkably narrow potential range of 1.30 V, which is significantly narrower compared to the six reductions of the paradigmatic carbon‐based electron acceptor C_60_ within 2.10 V.^[^ ^31^ ^]^

Thus, our findings clearly highlight the beneficial effect of cyclopentannulation and π‐expansion on the electron acceptor properties of the decacyclene scaffolds, while lateral substitution with additional electron donors and acceptors has only a subtle effect on the redox behavior.

**Figure 5:**
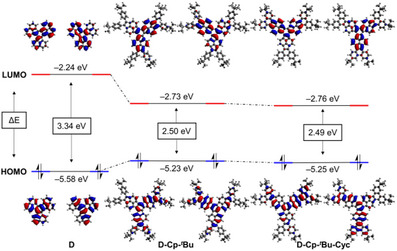
Kohn–Sham molecular orbitals (0.02 e^1/2^ Bohr) of **D**, **D‐Cp‐** *^t^* **Bu**, and **D‐Cp‐** *^t^* **Bu‐Cyc** calculated at the B3LYP(D3BJ)/6–311G(d,p) level of theory.

DFT calculations at the B3LYP^[^ ^32^ ^]^(D3BJ)/6–311G(d,p) level of theory were employed to assess the evolution of the electronic structure with focus on (anti)aromaticity upon cyclopentannulation and π‐expansion (Table 1 and the Supporting Information). In agreement with the X‐ray crystallographic data of **D‐Cp‐** *^t^* **Bu**, the calculated structures of all decacyclene derivatives possess a *D*_3_ symmetry resulting in a set of doubly degenerate frontier molecular orbitals (FMOs). Cyclopentannulation of the decacyclene core energetically elevates the HOMO and lowers the LUMO, thus narrowing the HOMO–LUMO gap by 0.84 eV from 3.34 eV for **D** to 2.50 eV for **D‐Cp‐** *^t^* **Bu**. Further cyclization of **D‐Cp‐** *^t^* **Bu** toward **D‐Cp‐** *^t^* **Bu‐Cyc** leaves the energy levels largely unaffected. Compared to **D‐Cp‐*^t^* Bu**, substitution with electron‐donating methoxy groups in **D‐Cp‐OMe** elevates both the HOMO and LUMO by 0.19 and 0.10 eV, respectively (see the Supporting Information). Conversely, substitution with electron‐withdrawing fluoro substituents in **D‐Cp‐F** lowers the HOMO and LUMO by 0.34 and 0.33 eV, respectively. The HOMO–LUMO gaps of all cyclopentannulated decacyclenes are comparable in the range of 2.41–2.51 eV (Figure 5).

**Figure 6:**
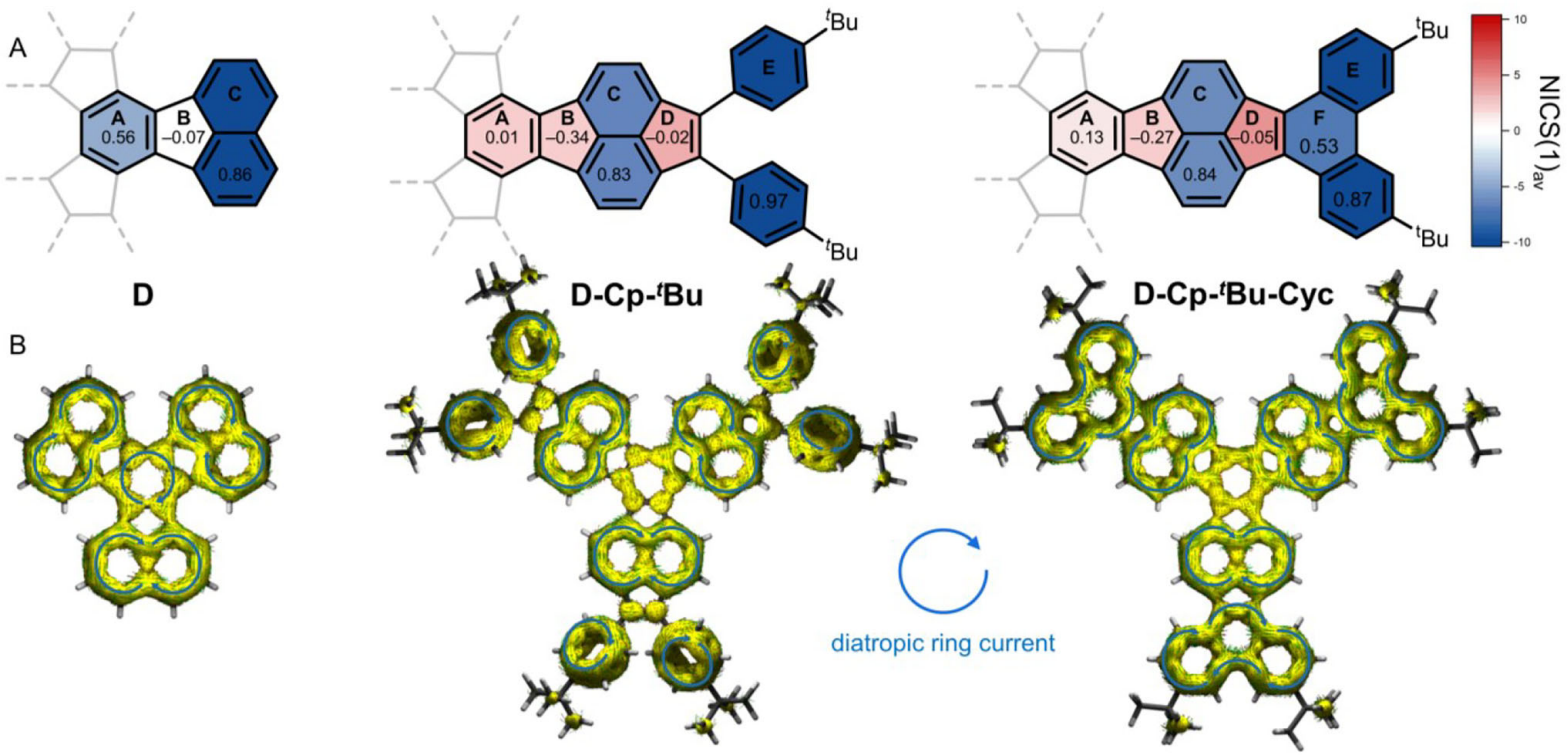
A) Schematic depiction of NICS(1)_av_ values of **D**, **D‐Cp‐** *^t^* **Bu**, and **D‐Cp‐** *^t^* **Bu‐Cyc** highlighted with a color code (for numerical values, see the Supporting Information) and HOMA values based on DFT‐optimized structures. B) π‐only ACID plots (isovalue 0.02 a.u.) for **D**, **D‐Cp‐** *^t^* **Bu**, and **D‐Cp‐** *^t^* **Bu‐Cyc**. Diatropic ring currents are highlighted with blue arrows with magnetic field vector oriented perpendicular to the viewing plane. All calculations were carried out at the B3LYP(D3BJ)/6‐311G(d,p) level of theory.

To elucidate the evolution of (anti)aromaticity within the compound series, nucleus‐independent chemical shifts (NICS(1)_av_) were calculated using the standard GIAO method^[^ ^33^ ^]^ at the B3LYP(D3BJ)/6–311G(d,p) level of theory (Figure 6 and the Supporting Information). To account for the nonplanarity of the decacyclene scaffolds, NICS(1)_av_, obtained as the average of NICS(+1) and NICS(–1), together with the harmonic oscillator model of aromaticity (HOMA from +1 (aromatic) to −1 (antiaromatic)) based on the DFT‐optimized geometries were used in the following discussion (for comparison with the X‐ray crystallographic data, see the Supporting Information).^[^ ^34^ ^]^ In addition, π‐only anisotropy of induced current density (ACID) was calculated at the same level of theory (for details, see Supporting Information).^[^ ^35^ ^]^ A combined discussion of these indices, including both magnetic and structural manifestations of (anti)aromaticity, is expected to provide a well‐rounded picture of the investigated compounds.

In parent **D**, the central six‐membered ring A (NICS(1)_av_: −4.80, HOMA: +0.56) and the naphthalene moieties C (NICS(1)_av_: −9.55, HOMA: +0.86) are clearly aromatic, which is also indicated by the diatropic ring currents. The five‐membered rings show no ring current and are essentially nonaromatic (NICS(1)_av_: +0.34, HOMA: −0.07). The cyclopentannulation toward **D‐Cp‐** *^t^* **Bu** renders the central six‐membered ring A essentially nonaromatic (NICS(1)_av_: +1.40, HOMA: +0.01), while the adjacent five‐membered ring B becomes weakly antiaromatic (NICS(1)_av_: +2.02, HOMA: −0.34). The reduced aromatic character of ring A in **D‐Cp‐** *^t^* **Bu** is in good agreement with the clearly discernible bond length alternation revealed by X‐ray crystallography (Figure 2).^37^ The additional five‐membered ring D is weakly antiaromatic (NICS(1)_av_: +3.22, HOMA: −0.02) and shows no ring current similar to rings A and B. Moreover, the aromaticity of the naphthalene subunit C in **D‐Cp‐** *^t^* **Bu** is also reduced (NICS(1)_av_: −6.82, HOMA: +0.83) and the six‐membered rings E are clearly aromatic (NICS(1)_av_: −9.75, HOMA: +0.97). Cyclization toward **D‐Cp‐** *^t^* **Bu‐Cyc** has only a negligible influence on the (anti)aromatic character of the polycyclic scaffold, and the corresponding indices remain largely unaffected. The newly formed six‐membered ring F displays a moderate aromatic character (NICS(1)_av_: −7.19, HOMA: +0.53). Consequently, a diatropic ring current in the newly formed phenanthrene moiety is observed. Lateral substitution with electron acceptors and donors in **D‐Cp‐F** and **D‐Cp‐OMe**, respectively, has basically no effect on the (anti)aromaticity characteristics (for details, see the Supporting Information). Overall, cyclopentannulation of **D** to **D‐Cp‐** *^t^* **Bu** considerably reduces the aromatic character of the entire polycyclic scaffold, which can most likely be attributed to the increased localization of the C═C bonds.^[^ ^6^, ^21^ ^]^

## 3 Conclusion

In summary, a modular synthetic route toward a series of unprecedented cyclopentannulated decacyclenes has been developed. The synthetic approach relies on a nickel‐mediated Yamamoto cyclotrimerization of novel dibrominated pyracylene precursors as the key step and allows the introduction of electron‐withdrawing, ‐donating, and solubilizing substituents. Subsequent oxidative cyclodehydrogenation provided for π‐expansion of the polycyclic scaffold. The X‐ray crystallographic study revealed the propeller‐shaped conformation of the decacyclene core with a pronounced bond length alternation of the central six‐membered ring. Although the effect of substitution on the UV/Vis absorption properties of the compounds is rather small, the electrochemical studies revealed their exceptional redox behavior compared to parent decacyclene. Depending on the substitution pattern, the compounds are capable of up to six reversible reduction steps within a particularly narrow potential range between −1.45 and −2.86 V under electrochemical conditions. Computational density functional theory studies provided further insights into the structural and optoelectronic properties of the compounds, revealing the major impact of cyclopentannulation on the (anti)aromatic character of the entire polycyclic framework.

The newly developed cyclopentannulated decacyclenes highlight the potential of strategic incorporation of π‐conjugated five‐membered rings into polycyclic carbon‐sp^2^ scaffolds to realize robust multielectron‐accepting compounds that are of potential interest for the development of organic charge storage materials. Future studies may focus on enhancing the redox activity of structurally related scaffolds by direct functionalization of the five‐membered ring‐containing core with electron‐withdrawing substituents or by further π‐expansion, in particular by incorporating additional five‐membered rings. In contrast, the outer aromatic units may primarily serve as sites for the introduction of solubilizing groups, as they have only a negligible influence on the optoelectronic properties of the polycyclic scaffold.

## Conflict of Interest

The authors declare no conflict of interest.

## Supporting information



Supporting Information

Supporting Information

## Data Availability

The data that support the findings of this study are available from the corresponding author upon reasonable request.
