# Supplementary material for: Cyclopentannulated Decacyclenes as Carbon‐Based Multistage Electron Acceptors
Source: Chem Asian J. 2025 Jun 10;20(17):e00551. doi: 10.1002/asia.202500551 (PMC12447868; doi:10.1002/asia.202500551)
Supplement: Supplementary file 1 — Supporting Information [file ASIA-20-e00551-s002.docx]

Cyclopentannulated Decacyclenes as Carbon‑Based Multistage Electron Acceptors

S. C. Eiden,^‡^ E. Misselwitz,^‡^ F. Rominger and M. Kivala*

‡ These authors contributed equally to this work.

**Table of Contents**

[1. Experimental Details 3](#_Toc196464136)

[1.1. General Reaction Conditions 3](#_Toc196464137)

[1.2. Instruments Used 3](#_Toc196464138)

[2. Synthesis 6](#_Toc196464139)

[3. NMR Data 24](#_Toc196464140)

[4 Mass Spectrometry 42](#_Toc196464141)

[5 HPLC Chromatograms of D-Cp-*^t^*Bu-Cyc and D-Cp-F 44](#_Toc196464142)

[6. X-Ray Crystallographic Data 45](#_Toc196464143)

[7. UV/Vis Absorption Spectroscopy Data 70](#_Toc196464144)

[8. Electrochemical Data 77](#_Toc196464145)

[9. Frontier Molecular Orbitals 84](#_Toc196464146)

[10. Theoretical UV/Vis Absorption Data 87](#_Toc196464147)

[11. Nucleus Independent Chemical Shift (NICS) 90](#_Toc196464148)

[12. Anisotropy of Induced Current Density (ACID) 94](#_Toc196464149)

[13. Harmonic Oscillator Model of Aromaticity (HOMA) 96](#_Toc196464150)

[14. References 97](#_Toc196464151)

1. Experimental Details
   1. General Reaction Conditions

All solvents and reagents were purchased at reagent grade from commercial suppliers (Merck/Sigma-Aldrich, TCI, Thermo Fisher Scientific, Acros Organics, Honeywell, BLD Pharmatech) and used without additional purification. Acenaphthylen-1(2*H*)-one was obtained from BLD Pharmatech and used without further purification. All reactions were performed in sealed Biotage microwave reaction vials (10−20 mL, or 2.0−5.0 mL in combination with aluminum caps and septa). Thin layer chromatography was monitored on ALUGRAM aluminum plates from Macherey-Nagel, coated with 0.20 mm SiO_2_, by irradiation with UV-light (λ= 365 and 254 nm). Flash column chromatography was carried out with SiO_2_ from Macherey-Nagel (technical grade 60 M, pore size 60 Å, 40−63 µm particle size).

- 1. Instruments Used

**Nuclear Magnetic Resonance (NMR)** spectra were recorded at room temperature (295 K) unless noted otherwise on a Bruker Avance III 300, 400, 500, 600 or 700 at the Institute of Organic Chemistry (Heidelberg University). Proton broad band decoupling was applied for ^13^C measurements.^13^C spectra of compounds including fluorine atoms were measured with ^1^H and ^19^F decoupling unless mentioned otherwise. Deuterated solvents were used as purchased from Merck/Sigma-Aldrich, Deutero or Eurisotop. Chemical shifts (reported in parts per million ppm) were referenced^[1]^ to *δ*_H_ = 7.26 ppm (CDCl_3_), 5.32 ppm (CD_2_Cl_2_) and 5.91 ppm (1,1,2,2-tetrachloroethane-*d*_2_) for ^1^H and *δ*_C_ = 77.16 ppm (CDCl_3_), 53.84 ppm (CD_2_Cl_2_) and 74.2 ppm (1,1,2,2-tetrachloroethane-*d*_2_) for ^13^C and interpreted with MestReNova Version 14.1.2-25024. Apparent multiplicity is reported as s (singlet), d (doublet), dd (doublet of doublets), t (triplet), or m (multiplet).

**High-Performance Liquid Chromatography** **(HPLC)** was performed on a setup from Shimadzu consisting of a LC20-AP preparative pump, a DGU-405 degassing unit, a CTO-40C column oven, an SPD-M40 diode array detector, an FCV-20AH2 valve unit, an FRC-10A fraction collector and an CBM-20A communication bus module. On the analytical scale a Macherey-Nagel 5 µm (250 x 4.60 mm) normal phase column equipped with a column protection system and a Macherey-Nagel 5 µm (3 mm x 4 mm) precolumn was used. On the preparative scale a Macherey-Nagel 5 µm (250 x 21 mm) normal phase column equipped with a column protection system and a Macherey-Nagel 5 µm (10 mm x 16 mm) precolumn was employed

**UV/Vis Absorption and Emission Spectra** were recorded on an Agilent Cary 60 UV/Vis spectrometer and measured in CH_2_Cl_2_ in the wavelength region of 230 to 800 nm under ambient conditions (rt: room temperature). The abbreviation br. (broad) sh. (shoulder) refers to saddle points or shoulders in the absorption spectrum. Fluorescence analysis was carried out employing a JASCO FP-8500 Fluorescence Spectrometer with a JASCO ILF-835 (100 mm) integrating sphere. The data obtained was interpreted with Spectra Manager from JASCO.

**X-Ray Crystallography.** Single crystals were obtained by slow gas phase diffusion under the given conditions. The Bruker APEX-II Quazar diffractometer (radiation MoKα, λ = 0.71073 Å) with a CCD area detector and the STOE Stadivari instrument (radiation CuKα, λ=1.54178 Å) with a Pilatus CCD area detector (0.5° ω-scans) were used for data collection by the X-Ray crystallography department of the Organic Chemical Institute of Heidelberg University. Structures were solved with the ShelXT^[2]^ structure solution program and refined against F^2^ with a full-matrix least-squares algorithm with ShelXL.^[3]^ Hydrogen atoms were treated with riding models. Graphic visualization and measurement of torsion angles were done with Mercury 2020.1.^[4]^

**Electrochemistry.** A BASi Cell Stand instrument with a glassy carbon disk working electrode (3.0 mm diameter), an Ag/AgCl (3 m NaCl) quasi-reference electrode, and a platinum wire auxiliary electrode were used to record the voltammograms. Before each measurement, a 0.1 m electrolyte solution of *n*-Bu_4_NPF_6_ in anhydrous THF was degassed by purging with nitrogen for 20 min. The respective compounds were measured at a scan rate of 149 mV s^–1^ followed by the addition of ferrocene as the internal standard and re-measurement. Square wave voltammograms (SWV) and differential pulse voltammograms (DPV) were measured under the same experimental conditions.

**Infrared Spectroscopy.** A JASCO FT/IR-6000 FTIR spectrometer was operated in ATR mode to record infrared spectra. The respective transmission spectra are baseline corrected, depicted in cm^‑1^ and labeled according to the following abbreviations: s (strong), m (medium), w (weak), and br (broad).

**Melting Point.** The melting point was determined on a Büchi M‑560 melting point apparatus in open capillaries. Decomp. refers to decomposition.

**Mass Spectrometry.** Mass spectra were obtained from the facility of Heidelberg University and recorded on a JEOL AccuTOF GCx (electron ionization (EI)) or a Bruker timsTOFfleX (matrix assisted laser desorption ionization (MALDI)) instrument.

**High‑Performance Liquid Chromatography.** High‑performance liquid chromatography (HPLC) was performed on a setup from Shimadzu consisting of a LC20‑AP preparative pump, a DGU‑405 degassing unit, a CTO‑40C column oven, an SPD‑M40 diode array detector, an FCV‑20AH_2_ valve unit, an FRC‑10A fraction collector and an CBM‑20A communication bus module. On the analytical scale, a Macherey‑Nagel 5 µm (250 x 4.60 mm) normal phase column equipped with a column protection system and a Macherey‑Nagel 5 µm (3 mm x 4 mm) precolumn was used. On the preparative scale, a Macherey‑Nagel 5 µm (250 x 21 mm) normal phase column equipped with a column protection system and a Macherey‑Nagel 5 µm (10 mm x 16 mm) precolumn was employed.

**Computational Details.** Quantum chemical calculations were performed using the Gaussian 16 program package.^[5]^ Ground state geometry optimizations were performed by employing the B3LYP^[6]^ functional, the 6-311G(d,p)^[7]^ basis set and Grimme`s D3 dispersion correction^[8]^ with BJ-damping.^[9]^ Thereby, ultra-tight convergence criteria of the respective computational method were used. Frequency calculations at the same level of theory were employed to verify the geometries as local minima possessing no imaginary frequencies. Nucleus independent chemical shifts (NICS) were calculated using the Gauge‑Independent Atomic Orbital (GIAO)^[10,11]^ approach, as implemented in Gaussian 16 at the GIAO-B3LYP(D3BJ)/6-311G(d,p) level of theory. In NICS calculations, dummy atoms are placed at the center of the ring above the ring plane, which is defined as the mean plane between all carbon atoms of the respective ring. NICS(1)_av_ values correspond to the arithmetic mean of the NICS(+1) and NICS(–1) values. Analysis of the results was done with the Multiwfn 3.8 software.^[12]^ Ring-current analysis was accomplished by using the Continuous Set of Gauge Transformations (CSGT)^[10,13]^ method at the CSGT‑B3LYP(D3BJ)/6-311G(d,p) level of theory and visualized using the ACID program package of the Herges group.^[14]^ Analysis of the  character of occupied orbitals were conducted with Multiwfn 3.8 software.^[12]^ Calculation of HOMA values was accomplished with py.aroma software.^[15]^ Excited state calculations were carried out within the time-dependent (TD) DFT approximation, using the CAM-B3LYP^[16]^ functional with the same basis-set and dispersion correction as above alongside the polarizable continuum model (PCM)^[17]^ for solvation using dichloromethane parameters. For every compound, the energetically lowest 50 excited states were calculated. A linewidth of 0.2 eV was assumed for the predicted UV/Vis spectra using the Gaussview 6 software. ^[18]^

1. Synthesis

Compounds **S11**,^[19]^ **S3**,^[19]^ **S6**,^[20]^ **S8**^[20]^ and **D**^[21]^ were synthesized according to literature procedures.

**Scheme S1.** Synthetic strategy towards cyclotrimers **D‑Cp‑*^t^*Bu**, **D‑Cp‑F**, **D‑Cp‑OMe** and **D‑Cp‑*^t^*Bu‑Cyc**. NBS = *N*-bromosuccinimide, DMF = *N,N*‑dimethylformamide, AIBN = azobisisobutyronitrile, cod = 1,5-cyclooctadiene, 2,2’-bipy = 2,2’-bipyridine, DDQ = 1,3 dichloro-5,6-dicyano‑1,4-benzoquinone, DBU = 1,8‑diazabiscycloundec-7-en, *p*-TsOH = *para*-toluenesulfonic acid, *o*‑DCB = *ortho*-dichlorobenzene, TfOH = trifluoromethanesulfonic acid.

**Diacenaphtho[1,2-*j*:1*'*,2*'*-l]fluoranthene (D).**^[21]^ A round-bottom flask was charged with acenaphthylen-1(2*H*)‑one (**S9**) (1.50 g, 8.92 mmol), *p*-toluenesulfonic acid monohydrate (5.94 g, 31.2 mmol), propionic acid (2.31 g, 2.34 mL, 31.2 mmol) and *o*-dichlorobenzene (7.59 mL). The reaction mixture was heated at 105 °C for 18 h. Upon cooling to room temperature, the mixture was poured into MeOH (200 mL) and filtered. The residue was washed with copious amounts of MeOH (500 mL) and EtOH (600 mL). Sequential recrystallization from hot CHCl_3_ (250 mL) and hot CH_2_Cl_2_ (200 mL) afforded **D**(89.7 mg, 199 µmol, 7%) as a yellow solid.

M.p.: 375–376°C (lit. 375-376 °C).^[21]^

*R*_f_ = 0.32 (SiO_2_, petroleum ether/CH_2_Cl_2_ 1:1).

^1^H NMR (700 MHz, 1,1,2,2-tetrachloroethane-*d_2_*, 393 K): *δ* = 8.82 (d, *J* = 7.0 Hz, 6H), 7.99 (d, *J* = 7.9 Hz, 6H), 7.84 (t, *J* = 7.5 Hz, 6H) ppm.

^13^C{^1^H} NMR (176 MHz, 1,1,2,2-tetrachloroethane-*d_2_*, 393 K): *δ* = 137.0, 135.7, 134.0, 130.4, 127.5, 126.8, 123.2 ppm.

HRMS (MALDI, DCTB): calcd. for C_36_H_18_ [M]^+^: 450.1403; found: 450.1406.

Analytical data are consistent with the literature.^[21,22]^

**5,6-Dibromo-1,2-dihydroacenaphthylene (S11).**^[19]^ A three-neck flask was charged with DMF (500 mL) and acenaphthene (170 g, 1.10 mol) and cooled to –5 °C. Over a period of 4 h, NBS (500 g, 2.76 mmol) was added in four portions. After complete addition, the reaction was allowed to warm to 10 °C and stirred for 20 h. The formed precipitate was filtered off and the crude product was recrystallized from hot CHCl_3_ (850 mL) to obtain **S11** (89.0 g, 286 mmol, 26%) as off-white crystals.

M.p.: 175 °C (lit. 172–173 °C).^[19]^

*R*_f_ = 0.60 (SiO_2_, PE).

^1^H NMR (400 MHz, CD_2_Cl_2_): δ = 7.80 (d, *J* = 7.4 Hz, 2H), 7.12 (d, *J* = 7.2 Hz, 2H), 3.31 (s, 4H) ppm.

^13^C{^1^H} NMR (101 MHz, CD_2_Cl_2_): δ = 148.0, 142.4, 136.3, 128.1, 121.5, 114.4, 30.5 ppm.

HRMS (EI): calcd. for C_12_H_8_Br_2_: 309.8987 [M]^+^; found: 309.8970.

Analytical data are consistent with the literature.^[19]^

**1,2-Bis(4-(*tert*-butyl)phenyl)ethyne (S3).** A suspension of 1‑iodo‑4‑(*tert*‑butyl)benzene (**S1**) (6.00 g, 4.08 mL, 23.1 mmol), 1-(*tert*-butyl)-4-ethynylbenzene (**S2**) (3.65 g, 4.15 mL, 23.1 mmol), CuI (878 mg, 4.61 mmol) and [Pd(PPh_3_)_4_] (266 mg, 231 μmol) in degassed NEt_3_(300 ml) was heated at 100 °C for 2 h. Upon cooling to room temperature, the mixture was filtered through a plug of SiO_2_ and the solvent was removed under reduced pressure. The crude product was purified by column chromatography (SiO_2_, PE) to **S3** (5.76 g, 19.9 mmol, 86%) as colorless crystals.

M.p.: 177 °C.

*R*_f_ = 0.26 (SiO_2_, PE).

^1^H NMR (400 MHz, CD_2_Cl_2_): δ = 7.46 (d, *J* = 8.6 Hz, 4H), 7.39 (d, *J* = 8.6 Hz, 4H), 1.33 (s, 18H) ppm.

^13^C{^1^H} NMR (101 MHz, CD_2_Cl_2_): δ = 152.0, 131.6, 125.9, 120.8, 89.2, 35.1, 31.3 ppm.

HRMS (EI): calcd. for C_22_H_26_: 290.2029 [M]^+^; found: 290.2028.

Analytical data are consistent with the literature.^[19]^

.

**1,2-Bis(4-fluorophenyl)ethyne (S6).**^[20]^ A dry pressure vessel was charged with CuI (182 mg, 955 μmol) and [Pd(PPh_3_)_2_Cl_2_] (335 mg, 477 μmol) under ambient conditions. Under an overpressure of N_2_, dry benzene (20 mL), 1‑fluoro‑4‑iodobenzene (**S4**) (2.12 g, 9.55 mmol), trimethylsilylacetylene (**S5**) (469 mg, 661 µL, 477 mmol) and H_2_O (86.0 μL), previously purged with N_2_ for 20 min, were added. After addition of DBU (7.27 g, 7.13 mL, 47.7 mmol), the vessel was sealed, and the reaction mixture was purged with N_2_ for 20 min. Subsequently, the reaction mixture was heated at 60 °C for 18 h. Upon cooling to room temperature, the mixture was poured into 2m HCl (100 mL) and extracted with Et_2_O (3 × 100 mL). The combined organic phases were washed with 1m aq. HCl (100 mL) and saturated aq. NaCl (100 mL) and then dried over MgSO_4_. The crude product was purified by column chromatography (SiO_2_, PE) to **S6** (846 mg, 3.96 mmol, 83%) as colorless crystals.

M.p.: 92–93 °C (lit. 92 °C).^[23]^

*R*_f_ = 0.35 (SiO_2_, PE).

^1^H NMR (400 MHz, CD_2_Cl_2_): δ = 7.52 (dd, *J* = 8.9, 5.4 Hz, 4H), 7.07 (t, *J* = 8.8 Hz, 4H) ppm.

^13^C{^1^H,^19^F} NMR (126 MHz, CD_2_Cl_2_): δ = 162.6, 133.4, 119.2, 115.6, 87.8 ppm.

^19^F{^1^H} NMR (470 MHz, CD_2_Cl_2_): δ = –111.6 ppm.

HRMS (EI): calcd. for C_14_H_8_F_2_: 214.0589 [M]^+^; found: 214.0600.

Analytical data are consistent with the literature.^[20]^

**1,2-Bis(4-methoxyphenyl)ethyne (S8).**^[20]^ A dry pressure vessel was charged with CuI (610 mg, 3.21 mmol) and [Pd(PPh_3_)_2_Cl_2_] (1.13 g, 1.60 mmol). Under an overpressure of N_2_, dry toluene (50 mL), 4-bromoanisole **S7** (6.00 g, 4.02 mL, 32.1 mmol), trimethylsilylacetylene (**S5**) (1.58 g, 2.22 mL, 16.0 mmol) and H_2_O (289 μL), previously purged with N_2_ for 20 min, were added. After addition of DBU (29.3 g, 28.8 mL, 192 mmol), the vessel was sealed, and the reaction mixture purged with N_2_ for 20 min. Subsequently, the reaction mixture was heated at 60 °C for 18 h. Upon cooling to room temperature, the mixture was poured into 2m aq. HCl (100 mL) and extracted with Et_2_O (3 × 100 mL). The combined organic phases were washed with 1M aq. HCl (100 mL) and saturated aq. NaCl (100 mL) and then dried over MgSO_4_. The crude product was purified by column chromatography (SiO_2_, PE/CH_2_Cl_2_ 4:1) to **S8** (1.60 g, 6.74 mmol, 42%) as a pale yellow solid.

M.p.: 150 °C (lit. 142 °C).^[23]^

*R*_f_ = 0.26 (SiO_2_, PE/CH_2_Cl_2_ 4:1).

^1^H NMR (400 MHz, CD_2_Cl_2_): δ = 7.44 (d, *J* = 8.8 Hz, 4H), 6.88 (d, *J* = 8.8 Hz, 4H), 3.82 (s, 6H) ppm.

^13^C{^1^H} NMR (101 MHz, CD_2_Cl_2_): δ = 160.0, 133.2, 116.0, 114.4, 88.2, 55.7 ppm.

HRMS (EI): calcd. for C_16_H_14_O_2_: 238.0988 [M]^+^; found: 238.0984.

The reported experimental data are consistent with the literature. ^[20]^

**5,6-Bis(4-(*tert*-butyl)phenyl)-1,2-dihydrocyclopenta[*fg*]acenaphthylene (4).**^[24]^ A pressure vessel was charged with 5,6-dibromo-1,2‑dihydroacenaphthylene (**S11**) (1.00 g, 3.21 mmol), 1,2-bis(4-(*tert*-butyl)phenyl)ethyne (**S3**) (1.12 g, 3.85 mmol), K_2_CO_3_ (664 mg, 4.81 mmol), Xantphos (100 mg, 17.3 mmol) and Pd(OAc)_2_ (36.0 mg, 160 µmol). The vessel was sealed and dry DMF (40 mL) was added. The reaction mixture was purged with N_2_ for 20 min and heated at 120 °C for 72 h. Upon cooling to room temperature, the mixture was filtered through a plug of Celite and the solvent was removed under reduced pressure. The product was isolated by column chromatography (SiO_2_, PE/CH_2_Cl_2_ 8:1) to yield **4** (738 mg, 1.67 mmol, 52%) as an orange solid.

M.p.: 172 °C.

*R*_f_ = 0.5 (SiO_2_, PE/CH_2_Cl_2_ 9:1).

^1^H NMR (400 MHz, CD_2_Cl_2_): δ = 7.79 (d, *J* = 7.0 Hz, 2H), 7.49 – 7.47 (m, 6H), 7.43 – 7.40 (m, 4H), 3.55 (s, 4H), 1.38 (s, 18H) ppm.

^13^C{^1^H} NMR (101 MHz, CD_2_Cl_2_): δ = 150.3, 147.3, 137.4, 136.4, 135.8, 133.9, 129.9, 127.3, 126.2, 125.7, 121.2, 34.9, 33.0, 31.6 ppm.

IR (FT-ATR): *ṽ* = 3732 (w), 3054 (w), 3030 (w), 2958 (s), 2189 (w), 2166 (w), 1618 (m), 1515 (m), 1467 (m), 1429 (s), 1361 (m), 1266 (m), 1205 (m), 1137 (m), 1115 (m), 1054 (m), 1021 (m), 948 (m), 838 (s), 783 (m), 739 (m), 644 (m), 561 (s), 532 (m) cm^–1^.

UV/Vis: (CH_2_Cl_2_): *λ*_max_ (*ε*) = 249 (63100), 334 (13800), 360 (15500), 431 (3100) nm (m^‑1^cm^‑1^).

HRMS (EI): calcd. for C_34_H_34_: 442.2655 [M]^+^; found: 442.2634.

The analytical data are consistent with the literature.^[24]^

**5,6-Bis(4-fluorophenyl)-1,2-dihydrocyclopenta[*fg*]acenaphthylene (5).** A microwave tube was charged with 5,6-dibromo-1,2-dihydroacenaphthylene (**S11**) (500 mg, 1.60 mmol), 1,2‑bis(4‑fluorophenyl)ethyne (**S6**) (412 mg, 1.92 mmol), K_2_CO_3_ (250 mg, 1.60 mmol), Xantphos (50.0 mg, 8.75 mmol) and Pd(OAc)_2_ (18.0 mg, 80.0 µmol). The tube was sealed and dry DMF (20 mL) was added. The reaction mixture was purged with N_2_ for 20 min and then heated for at 120 °C for 72 h. Upon cooling to room temperature, the mixture was filtered through a plug of Celite and the solvent was removed under reduced pressure. The product was isolated by column chromatography (SiO_2_, PE/CH_2_Cl_2_ 8:1) to yield **5** (228 mg, 624 μmol, 39%) as a yellow solid.

M.p.: 210 °C.

*R*_f_ = 0.6 (SiO_2_, PE/ CH_2_Cl_2_ 8:1).

^1^H NMR (400 MHz, CDCl_3_): δ = 7.78 (d, *J* = 7.0 Hz, 2H), 7.48 – 7.43 (m, 6H), 7.09 (t, *J* = 8.9 Hz, 4H), 3.56 (s, 4H) ppm.

^13^C{^1^H,^19^F} NMR (101 MHz, CDCl_3_): δ = 162.1, 147.3, 136.7, 135.6, 132.2, 131.6, 126.8, 126.1, 121.1, 115.7, 32.8 ppm. (one signal coincident or not observed)

^19^F{^1^H} NMR (470 MHz, CDCl_3_): δ = –115.0 ppm.

IR (FT-ATR): *ṽ* = 3044 (w), 2924 (w), 2823 (w), 2023 (w), 1894 (w), 1730 (w), 1598 (m), 1533 (m), 1509 (m), 1467 (m), 1421 (m), 1349 (m), 1295 (m), 1263 (m), 1217 (s), 1153 (m), 1091 (m), 1052 (m), 1014 (m), 837 (s), 809 (m), 758 (m), 663 (m), 641 (m), 603 (m), 568 (m) cm^-1^.

UV/Vis: (CH_2_Cl_2_): *λ*_max_ (*ε*) = 248 (53900), 333 (12400), 359 (13300), 421 (2400) nm (m^–1^cm^‑1^).

HRMS (EI): calcd. for C_26_H_16_F_2_: 366.1215 [M]^+^; found: 366.1206.

**5,6-Bis(4-methoxyphenyl)-1,2-dihydrocyclopenta[*fg*]acenaphthylene (6).** A microwave tube was charged with 5,6‑dibromo‑1,2-dihydroacenaphthylene (**S11**) (500 mg, 1.60 mmol), 1,2‑bis(4‑methoxyphenyl)-ethyne (**S8**) (458 mg, 1.92 mmol), K_2_CO_3_ (250 mg, 1.60 mmol), Xantphos (50.0 mg, 8.75 mmol) and Pd(OAc)_2_ (18.0 mg, 80.0 µmol). The tube was sealed and dry DMF (20 mL) was added. The reaction mixture was purged with N_2_ for 20 min and then heated at 120 °C for 72 h. Upon cooling to room temperature, the mixture was filtered through a plug of Celite and the solvent was removed under reduced pressure. The product was isolated by column chromatography (SiO_2_, PE/CH_2_Cl_2_ 1:2) to yield **6** (400 mg, 1.02 mmol, 64%) as an orange solid.

M.p.: 191 °C.

*R*_f_ = 0.6 (SiO_2_, CH_2_Cl_2_/PE 2:1).

^1^H NMR (400 MHz, CD_2_Cl_2_): δ = 7.76 (d, *J* = 7.0 Hz, 2H), 7.47 – 7.42 (m, 6H), 6.92 (d, *J* = 8.8 Hz, 4H), 3.84 (s, 6H), 3.54 (s, 4H) ppm.

^13^C{^1^H} NMR (101 MHz, CD_2_Cl_2_): δ = 159.2, 147.2, 136.8, 136.4, 135.7, 131.5, 129.3, 127.2, 125.8, 121.1, 114.3, 55.6, 32.9 ppm.

IR (FT-ATR): *ṽ* = 3035 (m), 2941 (m), 2923 (m), 2827 (m), 1605 (s), 1570 (w), 1532 (m), 1511 (s), 1468 (m), 1428 (s), 1354 (m), 1283 (m), 1263 (m), 1241 (s), 1170 (s), 1135 (m), 1101 (m), 1076 (m), 1055 (m), 1033 (s), 998 (m), 961 (m) cm^–1^.

UV/Vis: (CH_2_Cl_2_): *λ*_max_ (*ε*) = 249 (62800), 285 (15200), 336 (13900), 362 (15900), 378 (12600),
446 (3200) nm (m^–1^cm^–1^).

HRMS (EI): calcd. for C_28_H_22_O_2_: 390.1614 [M]^+^; found: 390.1595.

**1,2-Dibromo-5,6-bis(4-(*tert*-butyl)phenyl)cyclopenta[*fg*]acenaphthylene (7).** Compound **4** (500 mg, 1.13 mmol), NBS (804 mg, 4.52 mmol) and AIBN (185 mg, 1.13 mmol) were dissolved in dry MeCN (15 mL) and the mixture was purged with N_2_ for 20 min. The reaction was heated at 80 °C for 18 h. Upon cooling to room temperature, the mixture was poured into saturated aq. Na_2_S_2_O_3_ (200 mL) and extracted with CH_2_Cl_2_ (3 × 100 mL). The combined organic phases were washed with saturated aq. Na_2_S_2_O_3_ (100 mL) followed by saturated aq. NaCl (100 mL) and dried over MgSO_4_. After evaporation of the solvent under reduced pressure,the crude product was purified by column chromatography (SiO_2_, PE/CH_2_Cl_2_ 10:1) to yield **7** (299 mg, 497 µmol, 44%) as a dark green solid.

M.p.: 243 °C.

*R*_f_ = 0.6 (SiO_2_, PE/CH_2_Cl_2_ 8:1).

^1^H NMR (400 MHz, CD_2_Cl_2_): δ = 7.32 (d, *J* = 8.5 Hz, 4H), 7.21 (d, *J* = 8.6 Hz, 4H), 6.74 (d, *J* = 6.9 Hz, 2H), 6.68 (d, *J* = 6.9 Hz, 2H), 1.32 (s, 18H) ppm.

^13^C{^1^H} NMR (101 MHz, CD_2_Cl_2_): δ = 151.4, 144.8, 141.1, 139.3, 131.9, 131.6, 130.1, 128.7, 125.7, 125.2, 124.7, 124.6, 35.0, 31.4 ppm.

IR (FT-ATR): *ṽ* = 3034 (w), 2957 (w), 2901 (w), 2865 (w), 1604 (w), 1571 (w), 1509 (w), 1454 (m), 1408 (w), 1361 (w), 1285 (w), 1245 (w), 1217 (w), 1171 (w), 1160 (w), 1112 (w), 1094 (w), 998 (w), 970 (m), 837 (m), 823 (s), 798 (w), (m), 531 (w) cm^–1^.

UV/Vis: (CH_2_Cl_2_): *λ*_max_ (*ε*) = 296 (18600), 269 (21600), 356 (16900) nm (m^–1^cm^–1^).

HRMS (MALDI, DCTB): calcd. for C_34_H_30_Br_2_: 596.0714 [M]^+^; found: 596.0709.

**1,2-Dibromo-7,10-di-*tert*-butyldibenzo[*j,l*]cyclopenta[*cd*]fluoranthene (S12).** A Schlenk-tube was charged with compound **7** (50.0 mg, 83.5 µmol), dry CH_2_Cl_2_ (50.0 mL) was added and the reaction mixture was cooled to 0 °C. First, DDQ (20.9 mg, 91.9 µmol) was added followed by the dropwise addition of triflic acid (100 µL). The reaction mixture was stirred at 0 °C for 15 min. Subsequently, saturated aq. Na_2_CO_3_ (50 mL) was added. The resulting mixture was extracted with CH_2_Cl_2_ (3 × 50 mL) and the combined organic phases were washed with H_2_O (2 × 100 mL). Evaporation of the solvent under reduced pressuregave **S12** (48.0 mg, 80.5 µmol, quant.) as a dark red solid.

M.p.: 249 °C.

*R*_f_ = 0.5 (SiO_2_, PE/CH_2_Cl_2_ 8:1).

^1^H NMR (400 MHz, CD_2_Cl_2_): δ = 8.55 (d, *J* = 1.9 Hz, 2H), 7.99 (d, *J* = 8.6 Hz, 2H), 7.63 (d, *J* = 8.6, 1.9 Hz, 2H), 7.20 (d, *J* = 7.0 Hz, 2H), 6.60 (d, *J* = 6.9 Hz, 2H), 1.52 (s, 18H) ppm.

^13^C{^1^H} NMR (101 MHz, CD_2_Cl_2_): δ = 149.7, 141.9, 138.9, 136.8, 134.0, 131.5, 129.6, 128.0, 126.1, 125.7, 125.0, 124.7, 124.1, 119.3, 35.5, 31.5 ppm.

IR (FT-ATR): *ṽ* = 3725 (w), 3627 (w), 2952 (w), 2863 (w), 2360 (w), 2342 (w), 2294 (w), 2213 (w), 2164 (m), 2154 (w), 1997 (w), 1980 (w), 1969 (w), 1454 (w), 1408 (w), 1361 (s), 1160 (w), 1113 (w), 877 (w), 798 (w), 705 (w), 690 (w), 649 (w) cm^–1^.

UV/Vis: (CH_2_Cl_2_): *λ*_max_ (*ε*) = 250 (57100), 266 (52300), 318 (22800), 337 (19800), 352 (29800), 375 (20600), 424 (8400) nm (m^–1^cm^–1^).

HRMS (MALDI, DCTB): calcd. for C_34_H_28_Br_2_: 594.0552 [M]^+^; found: 594.0547.

**1,2-Dibromo-5,6-bis(4-fluorophenyl)cyclopenta[*fg*]acenaphthylene (8).** Compound **5** (200 mg, 546 µmol), NBS (389 mg, 2.18 mmol) and AIBN (89.6 mg, 546 µmol) were dissolved in dry MeCN (10 mL) and the mixture was purged with N_2_ for 20 min. The reaction mixture was heated at 100°C for 18 h. Upon cooling to room temperature, the mixture was poured into saturated aq. Na_2_S_2_O_3_ (100 mL) and extracted with CH_2_Cl_2_ (3 × 50 mL). The combined organic phases were washed with saturated aq. Na_2_S_2_O_3_ (50 mL) followed by saturated aq. NaCl (50 mL) and dried over MgSO_4_. After removal of the solvent under reduced pressure,the crude product was purified by column chromatography (SiO_2_, PE/CH_2_Cl_2_ 20:1) to yield **8** (69.0 mg, 131 µmol, 24%) as a dark green solid.

M.p.: 270 °C.

*R*_f_ = 0.5 (SiO_2_, PE/CH_2_Cl_2_ 10:1).

^1^H NMR (400 MHz, CD_2_Cl_2_): δ = 7.25 – 7.22 (m, 4H), 7.03 – 6.99 (m, 4H), 6.75 (d, *J* = 6.8 Hz, 2H), 6.71 (d, *J* = 6.8 Hz, 2H) ppm.

^13^C{^1^H,^19^F} NMR (126 MHz, CD_2_Cl_2_): δ = 162.7, 143.9, 140.7, 139.6, 131.2, 130.9, 130.5, 125.3, 124.9, 124.6, 116.4, 115.9 ppm.

^19^F{^1^H} NMR (470 MHz, CD_2_Cl_2_): δ = –113.8 ppm.

IR (FT-ATR): *ṽ* = 3013 (w), 2951 (w), 2865 (w), 2170 (w), 2003 (w), 1605 (w), 1511 (w), 1454 (w), 1410 (m), 1361 (w), 1246 (w), 1160 (w), 1094 (w), 1023 (w), 998 (w), 969 (s), 824 (w), 783 (w), 757 (w), 705 (w), 691 (w), 614 (w), 583 (w), 557 (w), 525 (w), 512 (s) cm^–1^.

UV/Vis: (CH_2_Cl_2_): *λ*_max_ (*ε*) = 288 (17000), 364 (19600), 383 (15900) nm (m^–1^cm^–1^).

HRMS (MALDI, DCTB): calcd. for C_26_H_12_F_2_Br_2_: 521.9248 [M]^+^; found: 521.9233.

**1,2-Dibromo-5,6-bis(4-methoxyphenyl)cyclopenta[*fg*]acenaphthylene (9).** Compound **6** (500 mg, 1.28 mmol), NBS (912 mg, 5.12 mmol) and AIBN (210 mg, 1.28 mmol) were dissolved in dry MeCN (20 mL) and the mixture was purged with N_2_ for 20 min. The reaction mixture was heated at 60 °C for 6 h. Upon cooling to room temperature, the mixture was poured into saturated aq. Na_2_S_2_O_3_ (200 mL) and extracted with CH_2_Cl_2_ (3 × 100 mL). The combined organic phases were washed with saturated aq. Na_2_S_2_O_3_ (100 mL) followed by saturated aq. NaCl (100 mL) and dried over MgSO_4_. After removal of the solvent under reduced pressure,the crude product was purified by column chromatography (SiO_2_, PE/CH_2_Cl_2_ 2:1). The resulting compound was further purified by HPLC to yield **9** (91 mg, 166 µmol, 20%) as a brown solid.

M.p.: 200 °C.

*R*_f_ = 0.6 (SiO_2_, PE/CH_2_Cl_2_ 2:1).

HPLC: *t*_R_ = 12.1 min (SiO_2_, 250 x 21 mm, flow = 20 mL/min, *n*-heptane/CH_2_Cl_2_ 3:2)

^1^H NMR (400 MHz, CD_2_Cl_2_): δ = 7.18 (d, *J* = 8.8 Hz, 4H), 6.82 (d, *J* = 8.8 Hz, 4H), 6.69 (d, *J* = 6.8 Hz, 2H), 6.65 (d, *J* = 6.8 Hz, 2H), 3.80 (s, 6H) ppm.

^13^C{^1^H} NMR (101 MHz, CD_2_Cl_2_): δ = 159.7, 144.8, 140.3, 139.2,131.5, 130.4, 130.4, 127.2, 124.8, 124.7, 124.5, 114.2, 55.6 ppm.

IR (FT-ATR): *ṽ* = 3003 (w), 2958 (w), 2832 (w), 2053 (w), 1909 (w), 1861 (w), 1664 (w), 1603 (m), 1570 (m), 1531 (w), 1453 (w), 1409 (w), 1344 (w), 1286 (s), 1241 (m), 1172 (m), 1102 (w), 1029 (m), 968 (m), 841 (m), 822 (m), 582 (m) cm^–1^.

UV/Vis: (CH_2_Cl_2_): *λ*_max_ (*ε*) = 292 (19900), 377 (21400) nm (m^–1^cm^–1^).

HRMS (MALDI, DCTB): calcd. for C_28_H_18_O_2_Br_2_: 543.9668 [M]^+^; found: 543.9667.

**1,2,7,8,13,14‑Hexakis(4‑(*tert*‑butyl)phenyl)cyclopenta[5,6]acenaphtho[1,2‑*j*]cyclopenta[5,6]acenaphtho-[1,2-*l*]cyclopenta[*cd*]fluoranthene (D‑Cp‑*^t^*Bu).** A microwave vial was charged with compound **7** (50.0 mg, 83.6 µmol), [Ni(cod)_2_] (46.0 mg, 167 µmol), 2,2’ bipyridine (50.0 mg, 320 µmol) and 1,5‑cyclooctadiene (54.2 mg, 334 µmol) in a glovebox. The vial was sealed and 1,4‑dioxane (4.0 mL) previously purged with N_2_ for 2 h was added. The reaction mixture was stirred at 120 °C for 24 h. Upon cooling to room temperature, the reaction was diluted with toluene (10 mL) and filtered through a plug of SiO_2_. The plug was thoroughly rinsed with toluene (200 mL) and the solvent was removed under reduced pressure. Acetone (40 mL) was added to the black residue and the suspension was ultrasonicated for 1 min and allowed to settle for 1 h. The dark precipitate was filtered off and washed with acetone (30 mL) to obtain **D‑Cp‑*^t^*Bu** (18.0 mg, 13.9 µmol, 52%) as a brown solid.

M.p.: > 400 °C.

*R*_f_ = 0.9 (SiO_2_, PE/ethyl acetate 2:1).

^1^H NMR (400 MHz, CD_2_Cl_2_/CS_2_ (75:25 vol%)): δ = 7.50 (d, *J* = 7.3 Hz, 6H), 7.28 (d, *J* = 8.4 Hz, 12H), 7.24 (d, *J* = 8.5 Hz, 12H), 7.17 (d, *J* = 7.0 Hz, 6H), 1.39 (s, 54H) ppm.

^13^C{^1^H} NMR (101 MHz, CD_2_Cl_2_/CS_2_ (75:25 vol%)): δ = 150.3, 140.6, 139.8, 139.4, 137.7, 134.2, 133.0, 129.7, 129.1, 125.6, 125.4, 124.4, 34.9, 31.8 ppm.

IR (FT-ATR): *ṽ* = 2955 (s), 2899 (w), 2185 (w), 2013 (m), 1986 (w), 1602 (w), 1509 (w), 1455 (w), 1435 (w), 1408 (w), 1360 (w), 1283 (w), 1268 (w), 1242 (w), 1160 (s), 811 (m), cm^‑1^.

UV/Vis: (CH_2_Cl_2_): *λ*_max_ (*ε*) = 273 (65900), 372 (26500), 454 (59800), 472 (62700) nm (m^‑1^cm^‑1^).

HRMS (MALDI, DCTB): calcd. for C_102_H_90_: 1314.7037 [M]^+^; found: 1314.7062.

**1,2,7,8,13,14‑Hexakis(4‑(*tert*‑butyl)phenyl)cyclopenta[5,6]acenaphtho[1,2‑*j*]cyclopenta[5,6]acenaphtho-[1,2-*l*]cyclopenta[*cd*]fluoranthene (D‑Cp‑*^t^*Bu‑Cyc).**

**Via oxidative cyclodehydrogenation from D‑Cp‑*^t^*Bu:**

A Schlenk tube was charged with **D‑Cp‑*^t^*Bu** (20.0 mg, 15.2 µmol), dry CH_2_Cl_2_ (20 mL) was added and the suspension was cooled to 0 °C. DDQ (11.4 mg, 50.2 µmol) was added, followed by dropwise addition of triflic acid (50.0 µL). The reaction mixture was stirred at 0 °C for 15 min and subsequently sat. aq. Na_2_CO_3_ (50.0 mL) was added. The mixture was extracted with CH_2_Cl_2_ (3 × 100 mL). The combined organic phases were washed with water (3 × 50 mL) and dried over MgSO_4_ to give **D‑Cp‑*^t^*Bu‑Cyc** (19.5 mg, 15.17 µmol, quant.) as a brown solid.

**Via Yamamoto cyclotrimerization from S12:**

A microwave vial was charged with **S12** (20.0 mg, 33.5 µmol), [Ni(cod)_2_] (18.4 mg, 67.1 µmol), 2,2’‑bipyridine (10.5 mg, 67.1 µmol) and 1,5-cyclooctadiene (7.26 mg, 67.1 µmol) in a glovebox. The vial was sealed and 1,4‑dioxane (2.0 mL), previously purged with N_2_ for 2 h, was added. The reaction mixture was heated at 120 °C for 24 h. Upon cooling to room temperature, the reaction was diluted with CH_2_Cl_2_ and the mixture was washed with H_2_O (3 × 50 mL). Upon drying of the organic phase with MgSO_4_and removal of the solventunder reduced pressure, acetone (40 mL) was added to the black residue and the suspension was ultrasonicated for 1 min. The suspension was allowed to settle for 1 h. The dark precipitate was filtered off and washed with acetone (30 mL) to obtain **D‑Cp‑*^t^*Bu‑Cyc** (6.50 mg, 4.92 µmol, 44%) as a brown solid.

M.p.: > 400 °C

^1^H NMR (700 MHz, 1,1,2,2-tetrachloroethane-*d_2_*, 408 K,): δ = 8.53 (s, 6H), 8.17 (d, 6H), 7.63 (s, 6H), 7.51 (d, *J* = 8.4 Hz, 6H), 7.42 (s, 6H), 1.58 (s, 54H) ppm.

^13^C{^1^H} NMR (176 MHz, 1,1,2,2-tetrachloroethane-*d_2_*, 408 K): Solubility too low to obtain a meaningful spectrum.

IR (FT-ATR): *ṽ* = 3079 (w), 3048 (w), 2951 (w), 2863 (w), 1705 (w), 1614 (w), 1555 (w), 1517 (w), 1462 (w), 1419 (w), 1394 (w), 1360 (w), 1298 (w), 1258 (w), 1201 (w), 1106 (w), 1022 (w), 999 (w), 962 (w), 911 (w), 877 (w), 818 (m), 743 (w), 723 (w), 690 (w), 646 (w) cm^–1^.

UV/Vis: (CH_2_Cl_2_): *λ*_max_ (*ε*) = 257 (59100), 348 (20200), 457 (30000), 479 (32800) nm (m^‑1^cm^‑1^).

HRMS (MALDI, DCTB): calcd. for C_102_H_84_: 1308.6568 [M]^+^; found: 1308.6567.

**1,2,7,8,13,14 Hexakis(4-fluorophenyl)cyclopenta[5,6]acenaphtho[1,2*‑j*]cyclopenta[5,6]acenaphtho[1,2*-l*]cyclopenta[*cd*]fluoranthene (D‑Cp‑F).** A microwave vial was charged with compound **8** (40.0 mg, 76.6 µmol), [Ni(cod)_2_] (52.7 mg, 191 µmol), 2,2’‑bipyridine (50.0 mg, 320 µmol) and 1,5-cyclooctadiene (54.2 mg, 334 µmol) in a glovebox. Solvent 1,4‑dioxane (4.0 mL), previously purged with N_2_ for 2 h, was added and the reaction mixture was heated at 120 °C for 24 h. Upon cooling to room temperature, the reaction mixture was diluted with toluene (50 mL). The mixture was washed with 2m aq. HCl (100 mL) and H_2_O (2 × 100 mL) and the solvents were removed under reduced pressure. Acetone (40 mL) was added to the black residue and the suspension was ultrasonicated for 1 min. The suspension was allowed to settle for 1 h. The dark precipitate was filtered off and washed with acetone (50 mL) to obtain **D‑Cp‑F** (10.0 mg, 10.2 µmol,40%) as a brown solid.

M.p.: > 400 °C.

*R*_f_ = 0.8 (SiO_2_, PE/ethyl acetate 1:1).

^1^H NMR (700 MHz, 1,1,2,2-tetrachloroethane-*d_2_*, 408 K): δ = 7.86 (d, *J* = 7.2 Hz, 3H), 7.82 (d, *J* = 7.3 Hz, 3H), 7.76 (d, *J* = 6.9 Hz, 3H), 7.54 (d, *J* = 6.9 Hz, 3H), 7.42 (dd, *J* = 8.5, 5.6 Hz, 12H), 7.05 (dt, *J* = 11.1, 8.7 Hz, 12H) ppm.

^13^C{^1^H} NMR (176 MHz, 408 K, 1,1,2,2-tetrachloroethane-*d_2_*, 408 K): δ = 161.8, 140.9, 139.6, 139.4, 138.0, 131.2, 131.1, 125.6, 124.8, 124.2, 115.7, 115.6 ppm.

^19^F{^1^H} NMR (282 MHz, 1,1,2,2-tetrachloroethane-*d_2_*, rt): δ = –112.9 ppm.

IR (FT-ATR): *ṽ* = 3036 (w), 2922 (w), 2851 (w), 1898 (w), 1717 (w), 1601 (w), 1529 (m), 1510 (m), 1475 (m), 1433 (m), 1415 (w), 1350 (w), 1297 (w), 1279 (w), 1181 (w), 1155 (m), 1092 (w), 1066 (w), 1051 (w), 1004 (w), 957 (m), 837 (m), 574 (m), 523 (m) cm^–1^.

UV/Vis: (CH_2_Cl_2_): *λ*_max_ (*ε*) = 268 (38000), 463 (35100) nm (m^–1^cm^–1^).

HRMS (MALDI, DCTB): calcd. for C_78_H_36_F_6_: 1086.2716 [M]^+^; found: 1086.2726.

**1,2,7,8,13,14-Hexakis(4-methoxyphenyl)cyclopenta[5,6]acenaphtho[1,2-*j*]cyclopenta[5,6]acenaphtho-
[1,2‑*l*]cyclopenta[*cd*]fluoranthene (D‑Cp‑OMe).** A microwave vial was charged with compound **9** (50 mg, 91.5 µmol), [Ni(cod)_2_] (50.3 mg, 183 µmol), 2,2’‑bipyridine (50.0 mg, 320 µmol) and 1,5‑cyclooctadiene (54.2 mg, 334 µmol) in a glovebox. The vial was sealed and 1,4‑dioxane (4.00 mL), previously purged with N_2_ for 2 h, was added under an overpressure of N_2_. The reaction was heated at 120 °C for 24 h. Upon cooling to room temperature, the reaction was diluted with CH_2_Cl_2_ (50 mL). The mixture was washed with 2m aq. HCl (100 mL) and H_2_O (2 × 100 mL) and the solvents were removed under reduced pressure. Acetone (40 mL) was added to the black residue and the suspension was ultrasonicated for 1 min. The suspension was allowed to settle for 1 h. The dark precipitate was filtered off and washed with acetone (50 mL) to obtain **D‑Cp‑OMe** (10.4 mg, 8.85 µmol, 29%) as a brown solid.

M.p.: > 400 °C.

^1^H NMR (700 MHz, 1,1,2,2-tetrachloroethane-*d_2_*, 408 K): δ = 7.67 (d, *J* = 7.3 Hz, 6H), 7.32 (d, *J* = 8.5 Hz, 12H), 7.26 (d, *J* = 7.2 Hz, 6H), 6.86 (d, *J* = 8.4 Hz, 12H), 3.84 (s, 18H) ppm.

^13^C{^1^H} NMR (176 MHz, 1,1,2,2-tetrachloroethane-*d_2_*, 408 K): δ = 159.5, 141.5, 139.6, 139.2, 137.7, 131.2, 130.9, 128.5, 125.3, 124.2, 120.6, 114.5, 55.7 ppm.

IR (FT-ATR): *ṽ* = 2919 (m), 2849 (w), 2060 (w), 2025 (w), 1714 (w), 1664 (w), 1604 (m), 1572 (w), 1527 (w), 1512 (m), 1462 (m), 1434 (m), 1352 (w), 1286 (m), 1172 (m), 1105 (w), 1031 (m), 998 (m), 956 (w), 833 (m), 815 (m), 756 (w), 527 (m) cm^–1^.

UV/Vis: (CH_2_Cl_2_): *λ*_max_ (*ε*) 270 (60800), 374 (25900), 457 (53600), 477 (56100) nm (m^‑1^cm^‑1^).

HRMS (MALDI, DCTB): calcd. for C_84_H_54_O_6_: 1158.3915 [M]^+^; found: 1158.3920.

1. NMR Data

**Figure S1.** ^1^H NMR spectrum of S11 (400 MHz, CD_2_Cl_2_), *H_2_O, **^+^**DMF.

**Figure S2.** ^13^C{^1^H} NMR spectrum of S11 (101 MHz, CD_2_Cl_2_).

**Figure S3.** ^1^H NMR spectrum of S3 (400 MHz, CD_2_Cl_2_), *H_2_O.

**Figure S4.** ^13^C{^1^H} NMR spectrum of S3 (101 MHz, CD_2_Cl_2_).

**Figure S5.** ^1^H NMR spectrum of S6 (400 MHz, CD_2_Cl_2_), *H_2_O.

**Figure S6.** ^13^C{^1^H,^19^F} NMR spectrum of S6 (126 MHz, CD_2_Cl_2_).

**Figure S7.** ^19^F{^1^H} NMR spectrum of **S6** (470 MHz, CD_2_Cl_2_).

**Figure S8.** ^1^H NMR spectrum of S8 (400 MHz, CD_2_Cl_2_), *H_2_O.

**Figure S9.** ^13^C{^1^H} NMR spectrum of S8 (101 MHz, CD_2_Cl_2_).

**Figure S10.** ^1^H NMR spectrum of 4 (400 MHz, CD_2_Cl_2_), *H_2_O.

**Figure S11.** ^13^C{^1^H} NMR spectrum of 4 (101 MHz, CD_2_Cl_2_).

**Figure S12.** ^1^H NMR spectrum of 5 (400 MHz, CDCl_3_), *H_2_O.

**Figure S13.** ^13^C{^1^H,^19^F} NMR spectrum of 5 (101 MHz, CDCl_3_).

**Figure S14.** ^19^F{^1^H} NMR spectrum of **5** (470 MHz, CDCl_3_).

**Figure S15.** ^1^H NMR spectrum of 6 (400 MHz, CD_2_Cl_2_), *H_2_O.

**Figure S16.** ^13^C{^1^H} NMR spectrum of 6 (101 MHz, CD_2_Cl_2_).

**Figure S17.** ^1^H NMR spectrum of 7 (400 MHz, CD_2_Cl_2_), *H_2_O.

**Figure S18.** ^13^C{^1^H} NMR spectrum of 7 (101 MHz, CD_2_Cl_2_).

**Figure S19.** ^1^H NMR spectrum of 8 (400 MHz, CD_2_Cl_2_), *H_2_O.

**Figure S20.** ^13^C{^1^H,^19^F} NMR spectrum of **8** (126 MHz, CD_2_Cl_2_).

**Figure S21.** ^19^F{^1^H} NMR spectrum of **8** (470 MHz, CD_2_Cl_2_).

**Figure S22.** ^1^H NMR spectrum of 9 (400 MHz, CD_2_Cl_2_).

**Figure S23.** ^13^C{^1^H} NMR spectrum of 9 (101 MHz, CD_2_Cl_2_).

**Figure S24.** ^1^H NMR spectrum of S12 (400 MHz, CD_2_Cl_2_).

**Figure S25.** ^13^C{^1^H} NMR spectrum of S12 (101 MHz, CD_2_Cl_2_).

**Figure S26.** ^1^H NMR spectrum of D‑Cp‑*^t^*Bu (400 MHz, CD_2_Cl_2_/CS_2_ (75:25 vol%)), *H_2_O, ^+^C_6_H_6_.

**Figure S27.** ^13^C{^1^H} NMR spectrum of **D‑Cp‑*^t^*Bu** (101 MHz, CD_2_Cl_2_/CS_2_ (75:25 vol%)), *CS_2_.

**Figure S28.** ^1^H NMR spectrum of **D‑Cp‑F** (700 MHz, 1,1,2,2-tetrachloroethane-*d_2_*, 408 K), *H_2_O ^+^unidentified impurity.

**Figure S29.** ^13^C{^1^H} NMR spectrum of **D‑Cp‑F** (176 MHz, 1,1,2,2-tetrachloroethane-*d_2_*, 408 K).

**Figure S30.** ^19^F{^1^H} NMR spectrum of **D‑Cp‑F** (282 MHz, 1,1,2,2-tetrachloroethane-*d_2_*).

**Figure S31.** ^1^H NMR spectrum of **D‑Cp‑**OMe (700 MHz, 1,1,2,2-tetrachloroethane-*d_2_*, 408 K), *H_2_O.

**Figure S32.** ^13^C{^1^H} NMR spectrum of D‑Cp‑OMe (176 MHz, 1,1,2,2-tetrachloroethane-*d_2_*, 408 K), *H_2_O.

**Figure S33.** ^1^H NMR spectrum of D‑Cp‑*^t^*Bu‑Cyc (700 MHz, 1,1,2,2-tetrachloroethane-*d_2_*, 408 K), *H_2_O.

**Figure S34.** ^1^H NMR spectrum of D (700 MHz, 1,1,2,2-tetrachloroethane-*d_2_*, 393 K), *H_2_O.

**Figure S35.** ^13^C{^1^H} NMR spectrum of D (176 MHz, 1,1,2,2-tetrachloroethane-*d_2_*, 393 K).

1. Mass Spectrometry


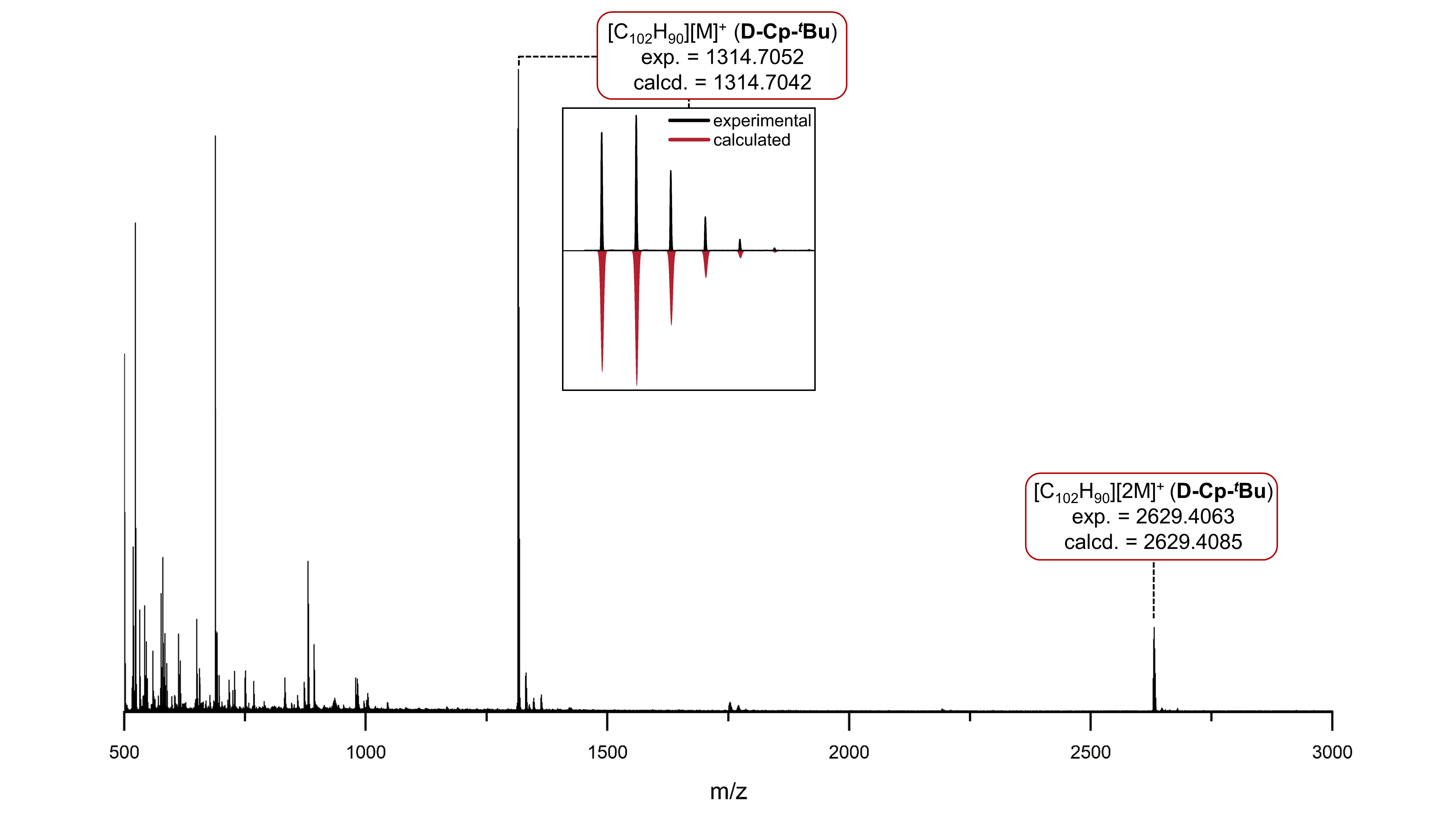


**Figure S36.** HRMS (MALDI, DCTB) of **D‑Cp‑*^t^*Bu**.

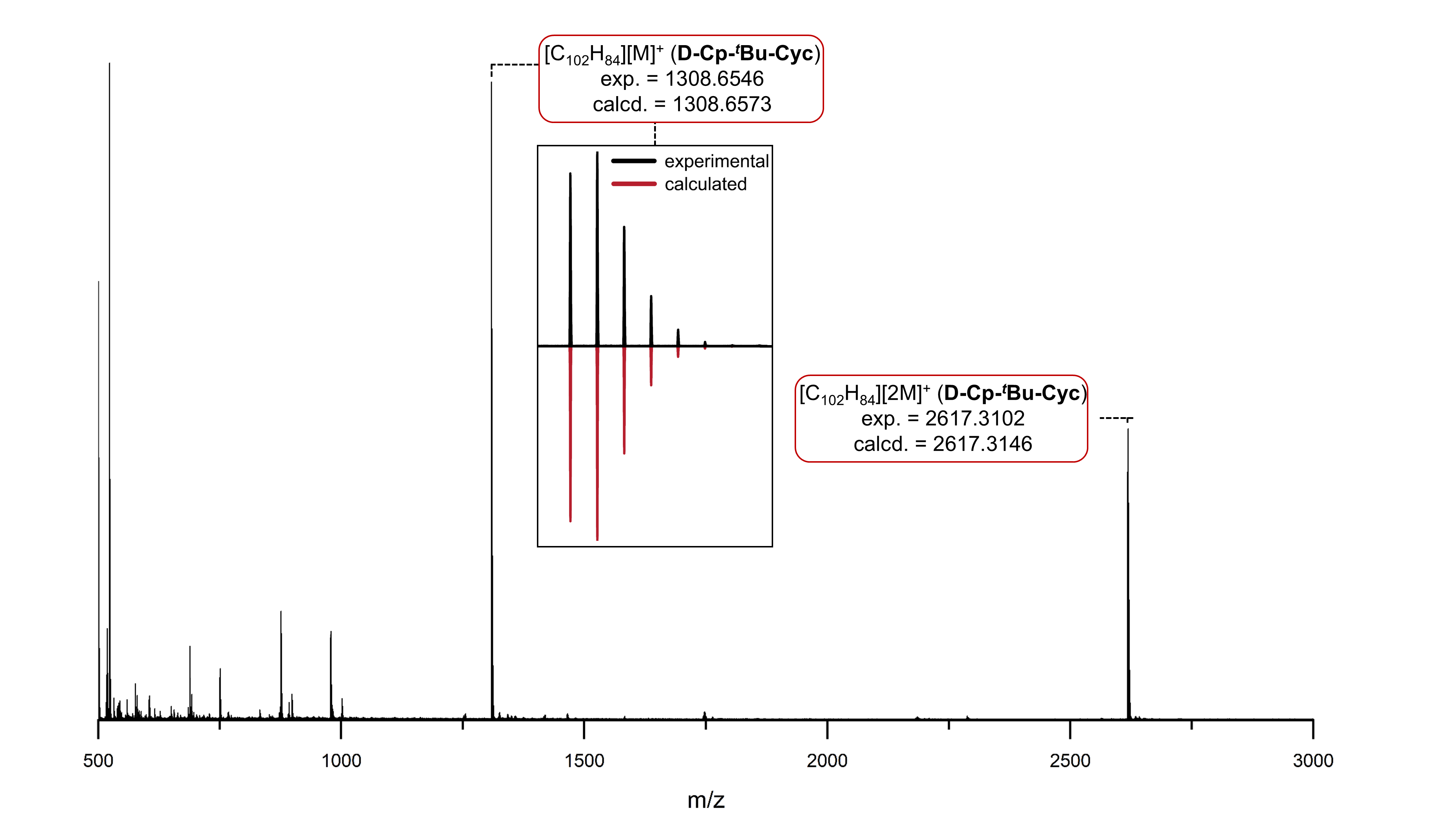


**Figure S37.** HRMS (MALDI, DCTB) of **D‑Cp‑*^t^*Bu‑Cyc**.


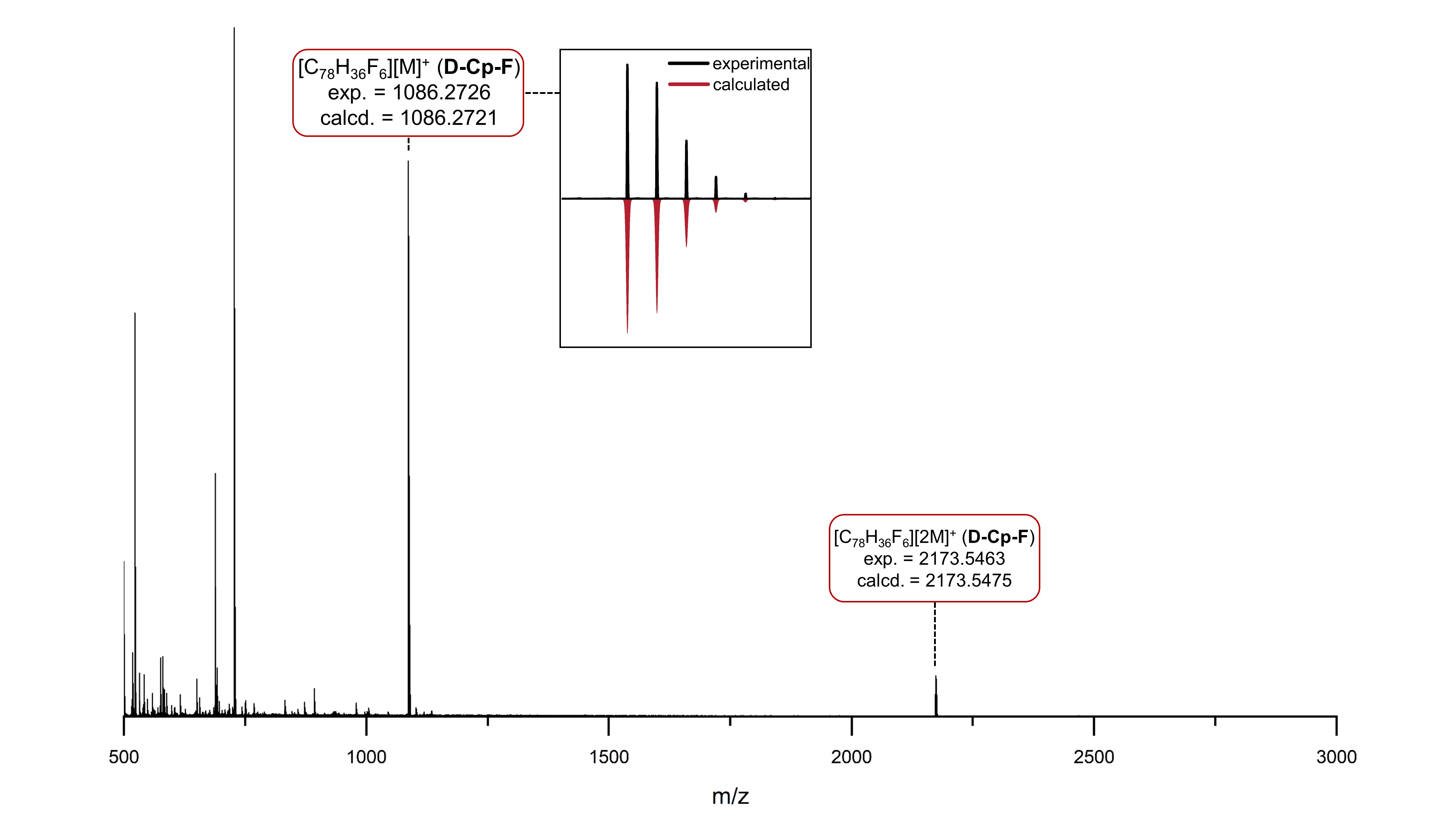


**Figure S38.** HRMS (MALDI, DCTB) of **D‑Cp‑F**.


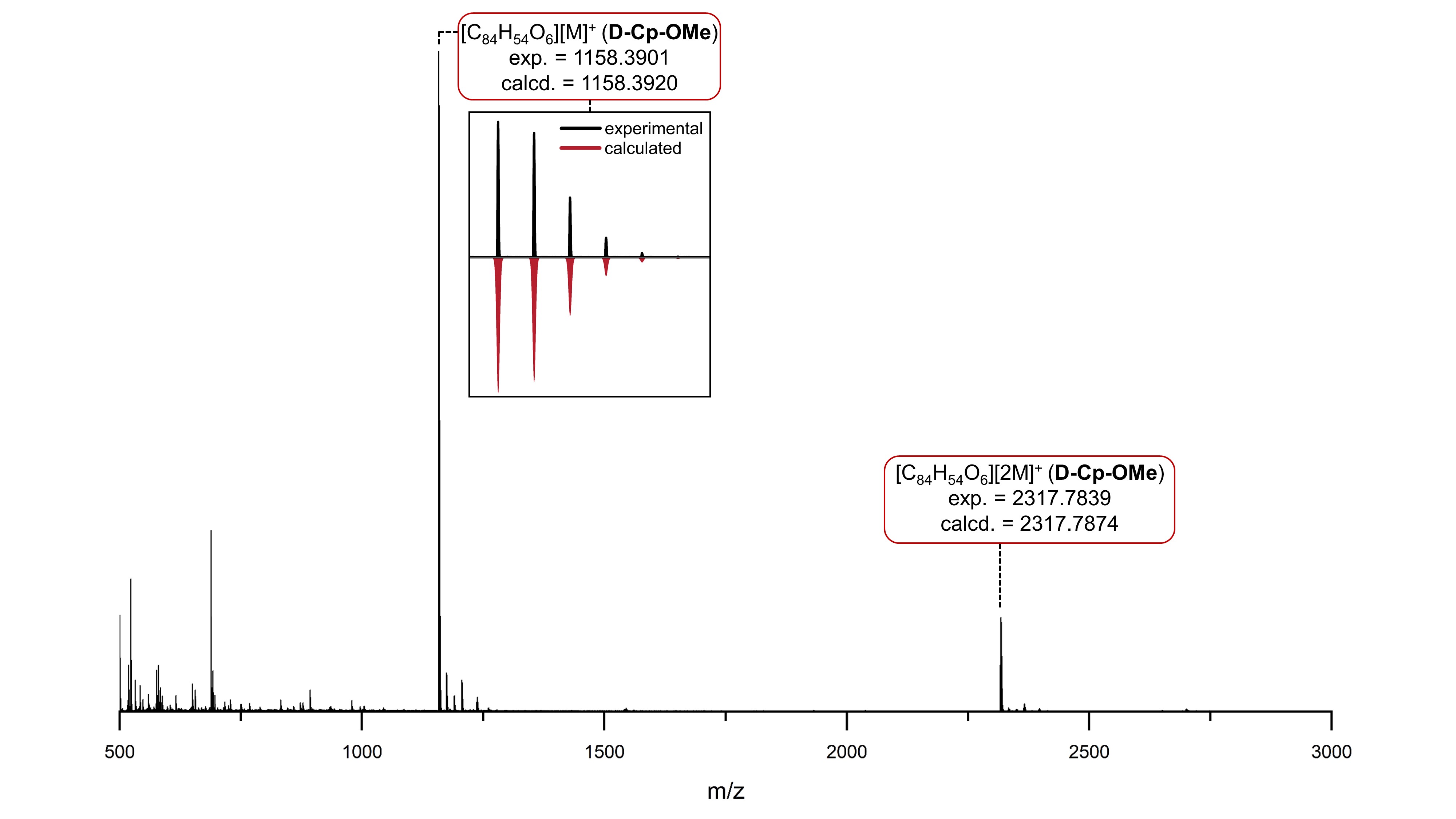


**Figure S39.** HRMS (MALDI, DCTB) of **D-Cp‑OMe**.

1. HPLC Chromatograms of D-Cp-*^t^*Bu-Cyc and D-Cp-F

**Figure S40.** HPLC chromatogram (toluene/*n*-heptane 1:1, analytical scale) of the sample of **D-Cp-F**, which was used for NMR studies at different detection wavelengths (466 nm, 444 nm, 374 nm).

**Figure S41.** HPLC chromatogram (toluene/*n*-heptane 1:2, analytical scale) of the sample of **D-Cp-*^t^*Bu-Cyc**, which was used for NMR studies at different detection wavelengths (466 nm, 444 nm, 374 nm).

1. X-Ray Crystallographic Data


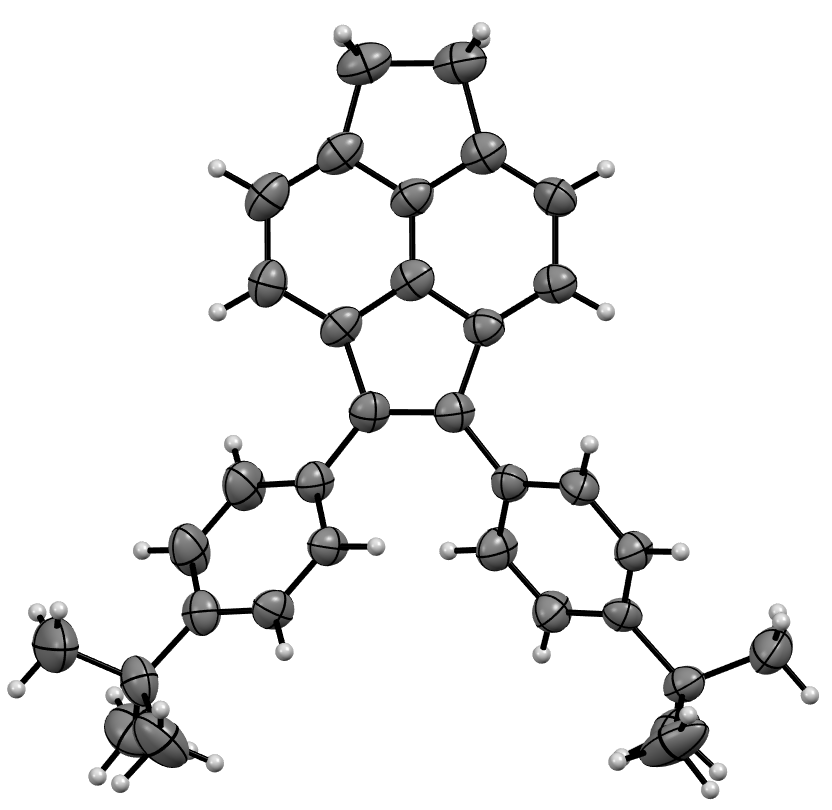


**Table S1.** X-ray crystallographic data and structure refinement of compound **4** obtained by vapor diffusion of MeOH into a solution of compound **4** in toluene at rt.

| CCDC | | 2440183 | |
| --- | --- | --- | --- |
| Empirical Formula | | C_34_H_34_ | |
| Formula Weight | | 442.61 | |
| Temperature / K | | 200(2) | |
| Wavelength / Å | | 1.54178 | |
| Crystal System | | monoclinic | |
| Space Group | | *P2_1/c_* | |
| Z | | 8 | |
| a / Å  b / Å  c / Å | α / deg  β / deg  γ / deg | 12.1421(9)  29.5584(17)  14.5789(11) | 90  91.546(6)  90 |
| Volume / Å^3^ | | 5230.5(6) | |
| Density (Calculated) / g/cm^3^ | | 1.124 | |
| Absorption Coefficient / mm^-1^ | | 0.471 | |
| Crystal Shape (Color) | | column (orange) | |
| Crystal Size / mm^3^ | | 0.053 × 0.050 × 0.032 | |
| Theta Range for Data Collection / deg | | 3.0 to 54.2 | |
| Index Ranges | | –12h12, –29k31, –8l≤15 | |
| Reflections Collected | | 26884 | |
| Reflections (Independent) | | 6380 (R(int) = 0.1646) | |
| Reflections (Observed) | | 3406 (I > 2σ(I)) | |
| Absorption Correction | | Semi-empirical from equivalents | |
| Max. and min. Transmission | | 0.99 and 0.85 | |
| Refinement Method | | Full-matrix least-squares on F^2^ | |
| Data/restraints/parameters | | 6380 / 606 / 625 | |
| Goodness-of-fit on F2 | | 1.16 | |
| Final R Indices (l > 2σ (l)) | | R1 = 0.105, wR2 = 0.161 | |
| Largest Diff. Peak and Hole / eÅ^–3^ | | 0.43 and –0.34 | |


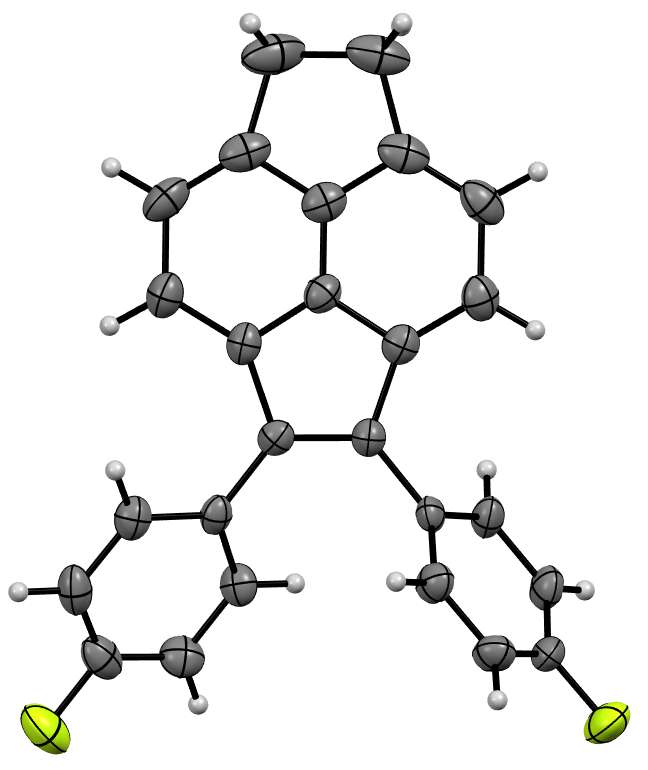


**Table S2.** X-ray crystallographic data and structure refinement of compound **5** obtained by vapor diffusion of MeOH into a solution of compound **5** in toluene at rt.

| CCDC | | 2440184 | |
| --- | --- | --- | --- |
| Empirical Formula | | C_26_H_16_F_2_ | |
| Formula Weight | | 366.39 | |
| Temperature / K | | 200(2) | |
| Wavelength / Å | | 0.71073 | |
| Crystal System | | monoclinic | |
| Space Group | | *P2_1/c_* | |
| Z | | 4 | |
| a / Å  b / Å  c / Å | α / deg  β / deg  γ / deg | 14.2211(18)  6.0472(7)  21.1080(3) | 90  102.1620(3)  90 |
| Volume / Å^3^ | | 1774.5(4) | |
| Density (Calculated) / g/cm^3^ | | 1.370 | |
| Absorption Coefficient / mm^-1^ | | 0.090 | |
| Crystal Shape (Color) | | pole (orange) | |
| Crystal Size / mm^3^ | | 0.832 × 0.022 × 0.020 | |
| Theta Range for Data Collection / deg | | 1.5 to 24.1 | |
| Index Ranges | | –16h16, –6k6, –24l≤24 | |
| Reflections Collected | | 14764 | |
| Reflections (Independent) | | 2812 (R(int) = 0.1037) | |
| Reflections (Observed) | | 1763 (I > 2σ(I)) | |
| Absorption Correction | | Semi-empirical from equivalents | |
| Max. and min. Transmission | | 0.96 and 0.55 | |
| Refinement Method | | Full-matrix least-squares on F^2^ | |
| Data/restraints/parameters | | 2812 / 0 / 253 | |
| Goodness-of-fit on F2 | | 1.06 | |
| Final R Indices (l > 2σ (l)) | | R1 = 0.055, wR2 = 0.111 | |
| Largest Diff. Peak and Hole / eÅ^–3^ | | 0.19 and –0.23 | |


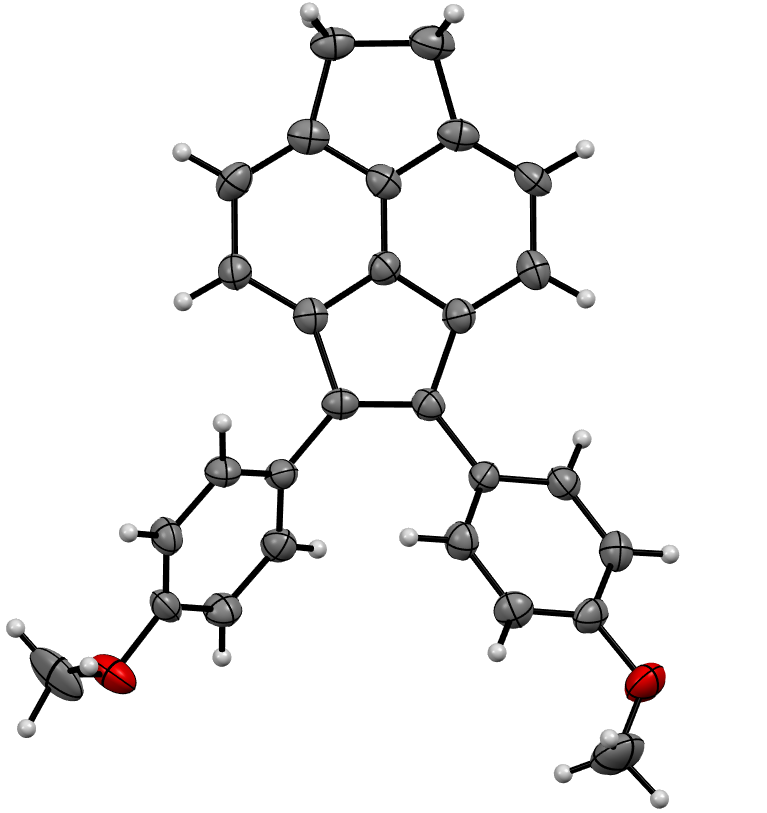


**Table S3.** X-ray crystallographic data and structure refinement of compound **6** obtained by vapor diffusion of MeOH into a solution of compound **6** in toluene at rt.

| CCDC | | 2440185 | |
| --- | --- | --- | --- |
| Empirical Formula | | C_28_H_22_O_2_ | |
| Formula Weight | | 390.45 | |
| Temperature / K | | 200(2) | |
| Wavelength / Å | | 0.71073 | |
| Crystal System | | monoclinic | |
| Space Group | | *I2/a* | |
| Z | | 8 | |
| a / Å  b / Å  c / Å | α / deg  β / deg  γ / deg | 23.2000(4)  6.1234(11)  28.3810(7) | 90  98.0620(4)  90 |
| Volume / Å^3^ | | 3991.9(14) | |
| Density (Calculated) / g/cm^3^ | | 1.300 | |
| Absorption Coefficient / mm^-1^ | | 0.080 | |
| Crystal Shape (Color) | | plank (orange) | |
| Crystal Size / mm^3^ | | 0.452 × 0.089 × 0.022 | |
| Theta Range for Data Collection / deg | | 1.4 to 23.2 | |
| Index Ranges | | –25h25, –6k6, –28l≤31 | |
| Reflections Collected | | 15348 | |
| Reflections (Independent) | | 2856 (R(int) = 0.1188) | |
| Reflections (Observed) | | 1764 (I > 2σ(I)) | |
| Absorption Correction | | Semi-empirical from equivalents | |
| Max. and min. Transmission | | 0.96 and 0.68 | |
| Refinement Method | | Full-matrix least-squares on F^2^ | |
| Data/restraints/parameters | | 2856 / 0 / 273 | |
| Goodness-of-fit on F2 | | 0.99 | |
| Final R Indices (l > 2σ (l)) | | R1 = 0.067, wR2 = 0.158 | |
| Largest Diff. Peak and Hole / eÅ^–3^ | | 0.22 and –0.23 | |


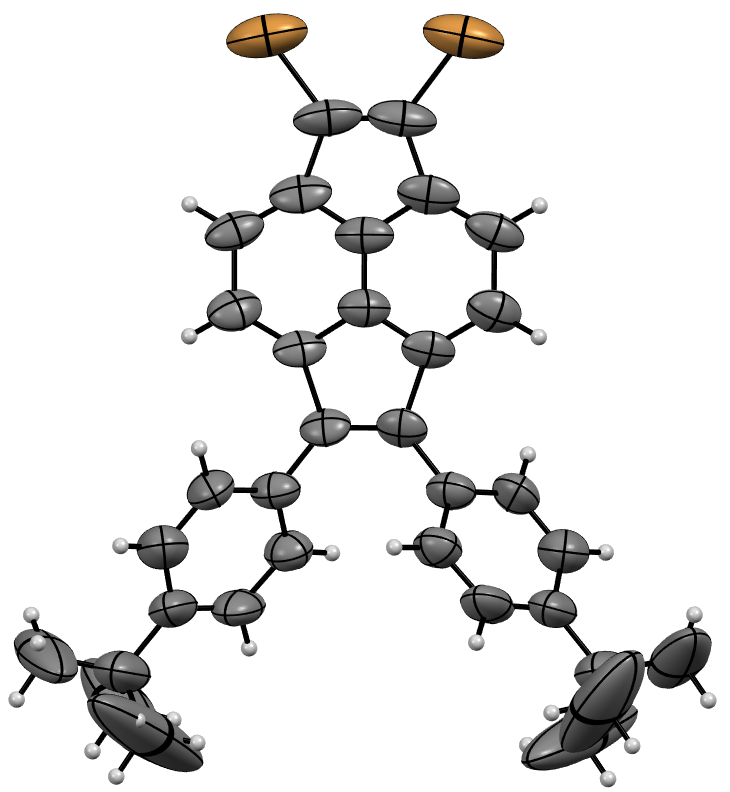


**Table S4.** X-ray crystallographic data and structure refinement of compound **7** obtained by vapor diffusion of MeOH into a solution of compound **7** in toluene at rt.

| CCDC | | 2440186 | |
| --- | --- | --- | --- |
| Empirical Formula | | C_34_H_30_Br_2_ | |
| Formula Weight | | 596.42 | |
| Temperature / K | | 200(2) | |
| Wavelength / Å | | 1.54178 | |
| Crystal System | | tetragonal | |
| Space Group | | *P4_1_2_1_2* | |
| Z | | 4 | |
| a / Å  b / Å  c / Å | α / deg  β / deg  γ / deg | 22.2491(8)  22.2491(8)  14.0453(7) | 90  90  90 |
| Volume / Å^3^ | | 6952.7(6) | |
| Density (Calculated) / g/cm^3^ | | 1.320 | |
| Absorption Coefficient / mm^-1^ | | 3.140 | |
| Crystal Shape (Color) | | plank (brown) | |
| Crystal Size / mm^3^ | | 0.196 × 0.034 × 0.012 | |
| Theta Range for Data Collection / deg | | 2.8 to 49.7 | |
| Index Ranges | | –22h21, –22k22, –8l≤13 | |
| Reflections Collected | | 34994 | |
| Reflections (Independent) | | 3535 (R(int) = 0.1611) | |
| Reflections (Observed) | | 1548 (I > 2σ(I)) | |
| Absorption Correction | | Semi-empirical from equivalents | |
| Max. and min. Transmission | | 1.00 and 0.88 | |
| Refinement Method | | Full-matrix least-squares on F^2^ | |
| Data/restraints/parameters | | 3535 / 447 / 380 | |
| Goodness-of-fit on F2 | | 0.95 | |
| Final R Indices (l > 2σ (l)) | | R1 = 0.053, wR2 = 0.111 | |
| Largest Diff. Peak and Hole / eÅ^–3^ | | 0.31 and –0.21 | |


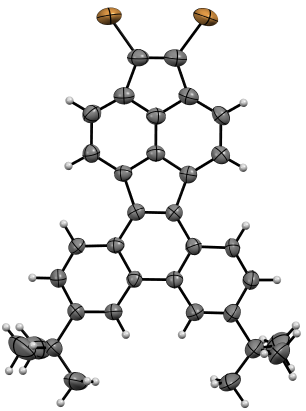


**Table S5.** X-ray crystallographic data and structure refinement of compound **S12** obtained by vapor diffusion of MeOH into a solution of compound **S12** in toluene at rt.

| CCDC | | 2440187 | |
| --- | --- | --- | --- |
| Empirical Formula | | C_36.33_H_30.67_Br_2.03_ | |
| Formula Weight | | 629.73 | |
| Temperature / K | | 200(2) | |
| Wavelength / Å | | 1.54178 | |
| Crystal System | | monoclinic | |
| Space Group | | *P2_1_/n* | |
| Z | | 24 | |
| a / Å  b / Å  c / Å | α / deg  β / deg  γ / deg | 26.3143(5)  20.1366(6)  35.3764(7) | 90  111.712(1) 90 |
| Volume / Å^3^ | | 17415.4(7) | |
| Density (Calculated) / g/cm^3^ | | 1.44 | |
| Absorption Coefficient / mm^-1^ | | 3.76 | |
| Crystal Shape (Color) | | irregular (brown) | |
| Crystal Size / mm^3^ | | 0.071 x 0.065 x 0.060 | |
| Theta Range for Data Collection / deg | | 1.8 to 63.7 | |
| Index Ranges | | –30h16, –23k22, –41l≤40 | |
| Reflections Collected | | 135583 | |
| Reflections (Independent) | | 28459 (R(int) = 0.1279) | |
| Reflections (Observed) | | 15295 (I > 2σ(I)) | |
| Absorption Correction | | Semi-empirical from equivalents | |
| Max. and min. Transmission | | 0.75 and 0.59 | |
| Refinement Method | | Full-matrix least-squares on F^2^ | |
| Data/restraints/parameters | | 28459 / 2025 / 2085 | |
| Goodness-of-fit on F2 | | 1.01 | |
| Final R Indices (l > 2σ (l)) | | R1 = 0.062, wR2 = 0.143 | |
| Largest Diff. Peak and Hole / eÅ^–3^ | | 0.71 and –0.51 | |


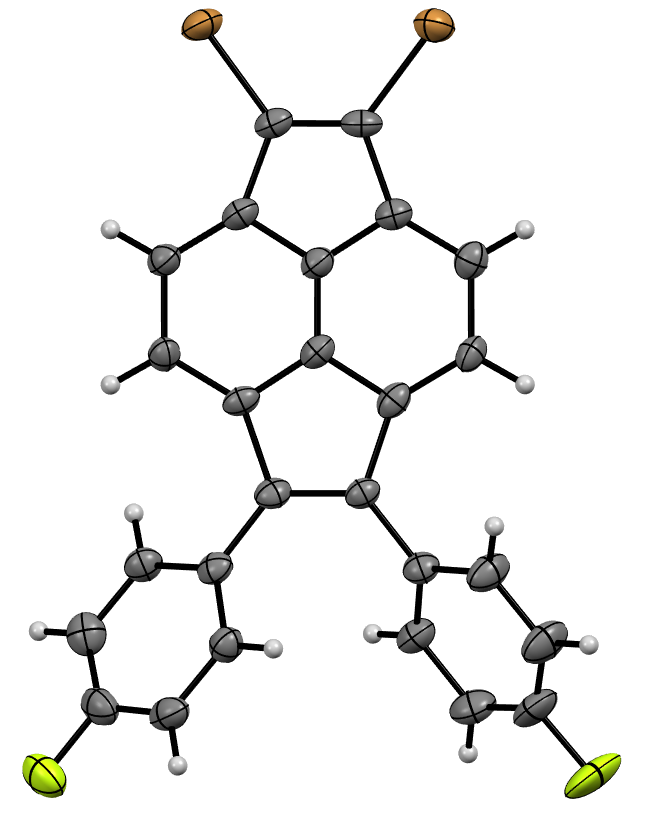


**Table S6.** X-ray crystallographic data and structure refinement of compound **8** obtained by vapor diffusion of MeOH into a solution of compound **8** in toluene at rt.

| CCDC | | 2440188 | |
| --- | --- | --- | --- |
| Empirical Formula | | C_26_H_12_Br_2_F_2_ | |
| Formula Weight | | 522.18 | |
| Temperature / K | | 200(2) | |
| Wavelength / Å | | 0.71073 | |
| Crystal System | | monoclinic | |
| Space Group | | *P2_1/c_* | |
| Z | | 4 | |
| a / Å  b / Å  c / Å | α / deg  β / deg  γ / deg | 21.7164(16)  12.6005(9)  7.3146(6) | 90  90.1900(2)  90 |
| Volume / Å^3^ | | 2001.8(3) | |
| Density (Calculated) / g/cm^3^ | | 1.730 | |
| Absorption Coefficient / mm^-1^ | | 4.080 | |
| Crystal Shape (Color) | | needle (brown) | |
| Crystal Size / mm^3^ | | 0.069 × 0.030 × 0.016 | |
| Theta Range for Data Collection / deg | | 0.9 to 26.0 | |
| Index Ranges | | –26h26, –15k15, –9l≤9 | |
| Reflections Collected | | 20138 | |
| Reflections (Independent) | | 3927 (R(int) = 0.0624) | |
| Reflections (Observed) | | 2829 (I > 2σ(I)) | |
| Absorption Correction | | Semi-empirical from equivalents | |
| Max. and min. Transmission | | 0.94 and 0.84 | |
| Refinement Method | | Full-matrix least-squares on F^2^ | |
| Data/restraints/parameters | | 3927 / 0 / 271 | |
| Goodness-of-fit on F2 | | 1.11 | |
| Final R Indices (l > 2σ (l)) | | R1 = 0.039, wR2 = 0.094 | |
| Largest Diff. Peak and Hole / eÅ^–3^ | | 1.04 and –0.50 | |


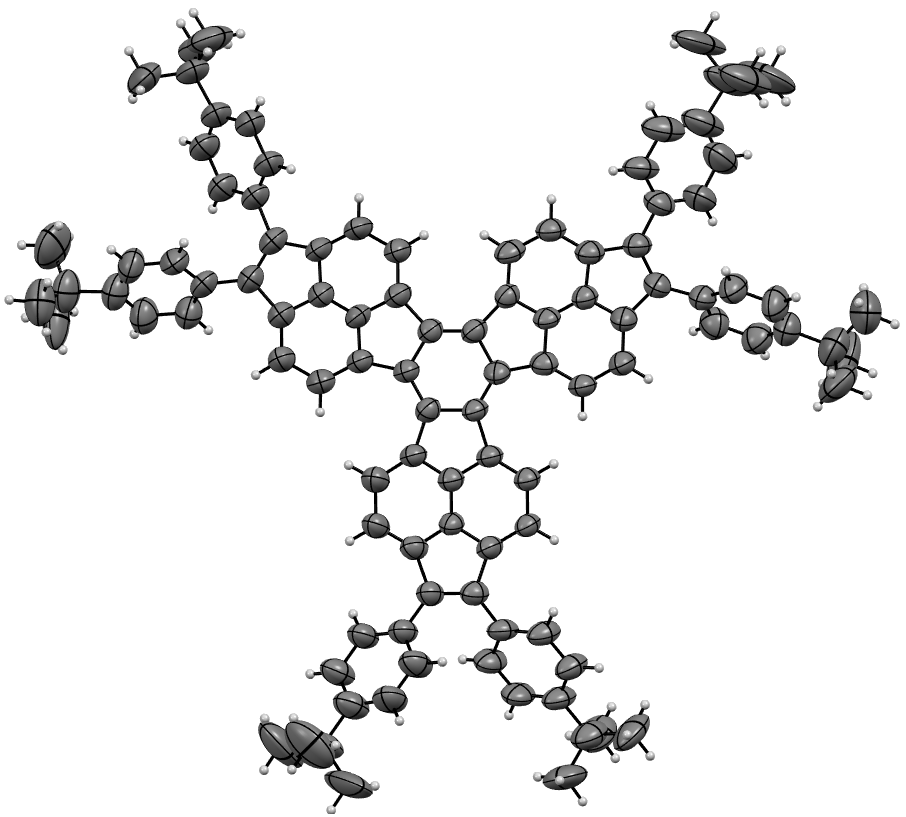


**Table S7.** X-ray crystallographic data and structure refinement of compound **D‑Cp‑*^t^*Bu** obtained by vapor diffusion of MeOH into a solution of compound **D‑Cp‑*^t^*Bu** in THF at rt.

| CCDC | | 2440189 | |
| --- | --- | --- | --- |
| Empirical Formula | | C_102_H_90_ | |
| Formula Weight | | 1315.73 | |
| Temperature / K | | 200(2) | |
| Wavelength / Å | | 1.54178 | |
| Crystal System | | triclinic | |
| Space Group | | *P1* | |
| Z | | 3 | |
| a / Å  b / Å  c / Å | α / deg  β / deg  γ / deg | 11.8100(3)  24.6262(6)  25.9785(6) | 114.146(2)  101.326(2)  95.130(2) |
| Volume / Å^3^ | | 6637.4(3) | |
| Density (Calculated) / g/cm^3^ | | 0.99 | |
| Absorption Coefficient / mm^-1^ | | 0.42 | |
| Crystal Shape (Color) | | plank (brown) | |
| Crystal Size / mm^3^ | | 0.448 × 0.094 × 0.023 | |
| Theta Range for Data Collection / deg | | 1.9 to 53.4 | |
| Index Ranges | | –12h10, –25k25, –22l≤27 | |
| Reflections Collected | | 63293 | |
| Reflections (Independent) | | 21723 (R(int) = 0.1374) | |
| Reflections (Observed) | | 13843 (I > 2σ(I)) | |
| Absorption Correction | | Semi-empirical from equivalents | |
| Max. and min. Transmission | | 0.99 and 0.91 | |
| Refinement Method | | Full-matrix least-squares on F^2^ | |
| Data/restraints/parameters | | 21723 / 18075 / 2755 | |
| Goodness-of-fit on F2 | | 0.99 | |
| Final R Indices (l > 2σ (l)) | | R1 = 0.075, wR2 = 0.200 | |
| Largest Diff. Peak and Hole / eÅ^–3^ | | 0.24 and -0.19 | |


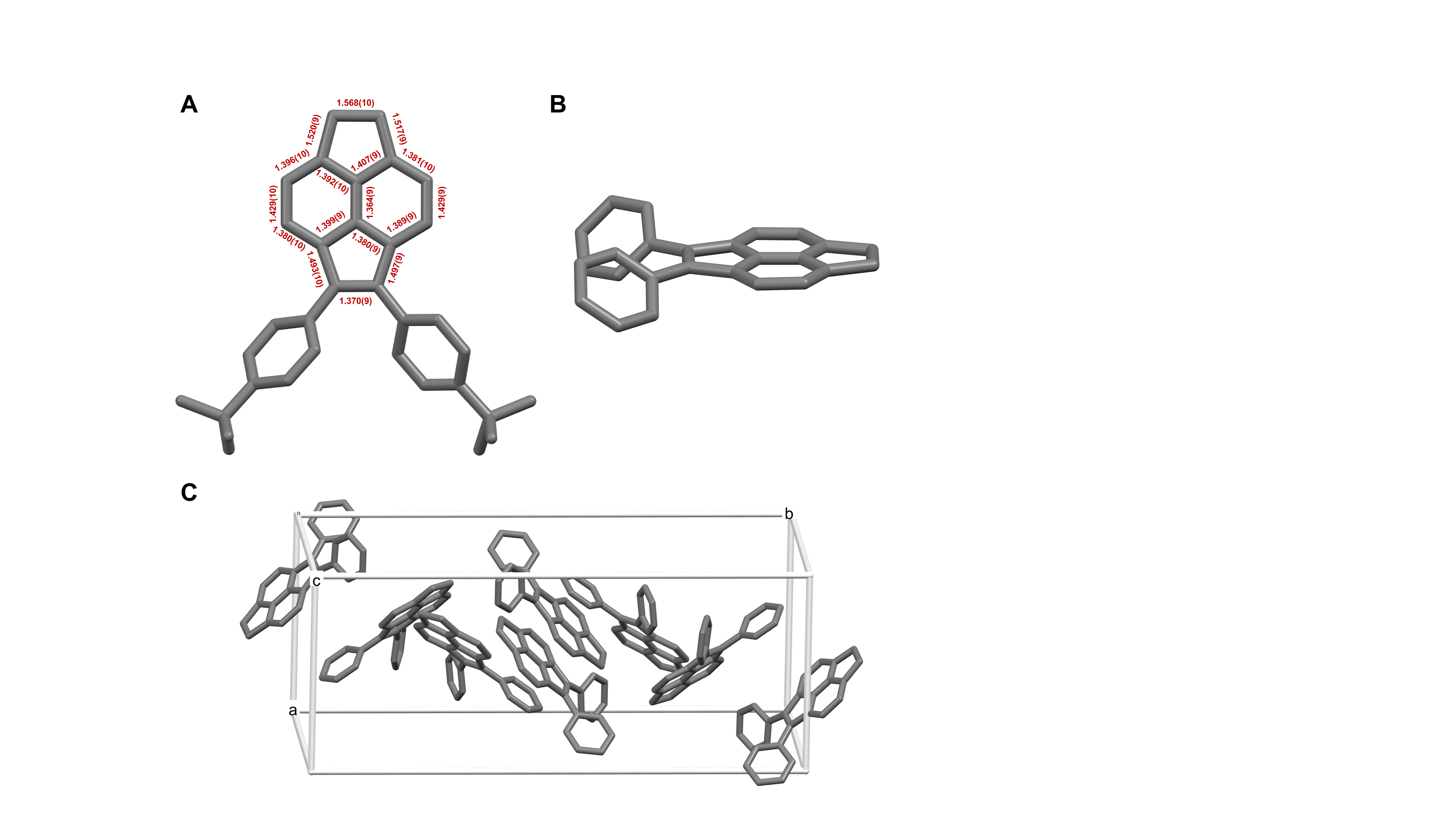


**Figure S42.** Solid state characterization of **4**. Color code: carbon (grey). a) Top view with bond lengths of the core scaffold given in Å. b) Side view of the molecule. c) Visualization of the unit cell.


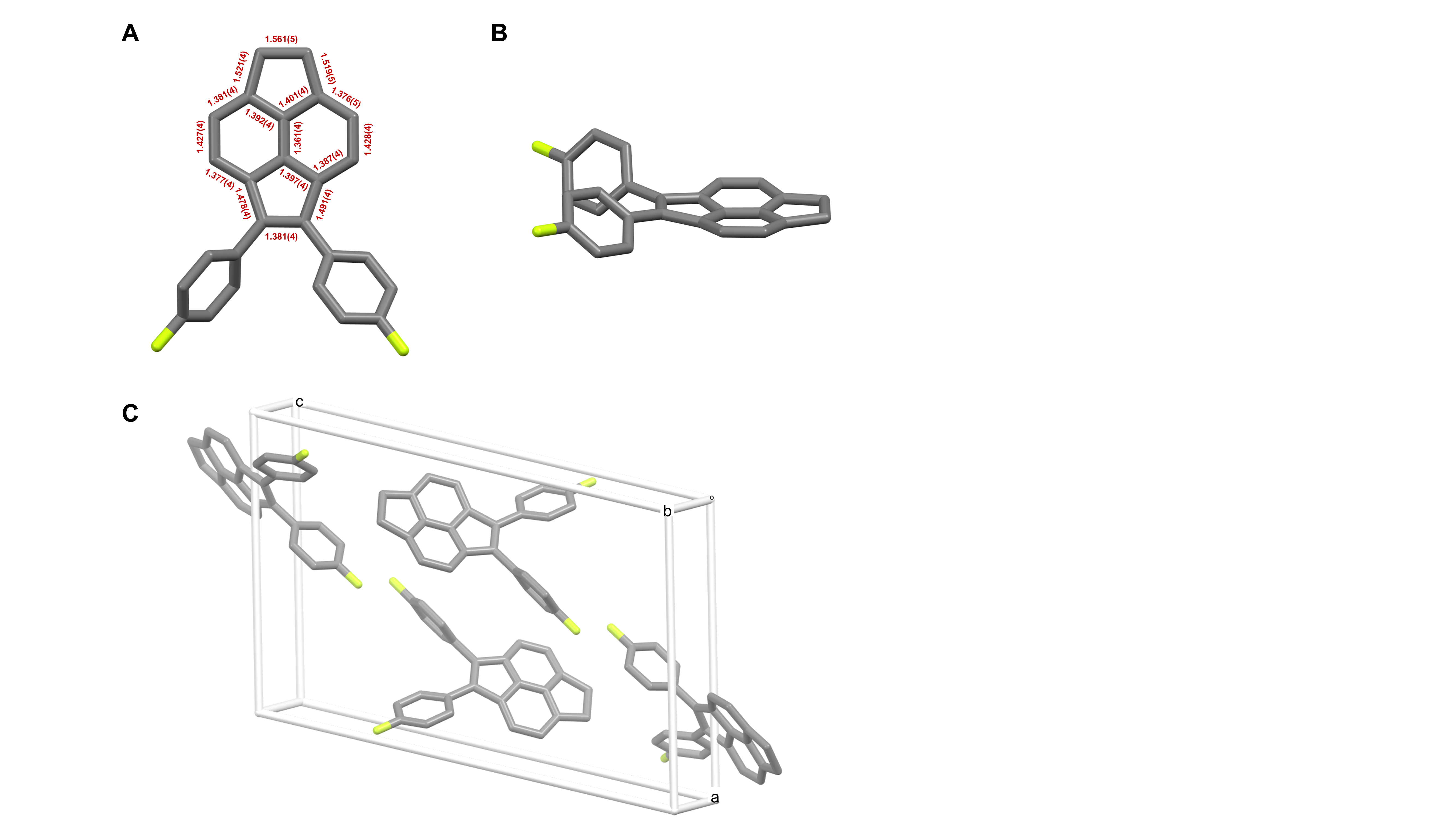


**Figure S43.** Solid state characterization of **5**. Color code: carbon (grey), fluorine (yellow). a) Top view with bond lengths of the core scaffold given in Å. b) Side view of the molecule. c) Visualization of the unit cell.


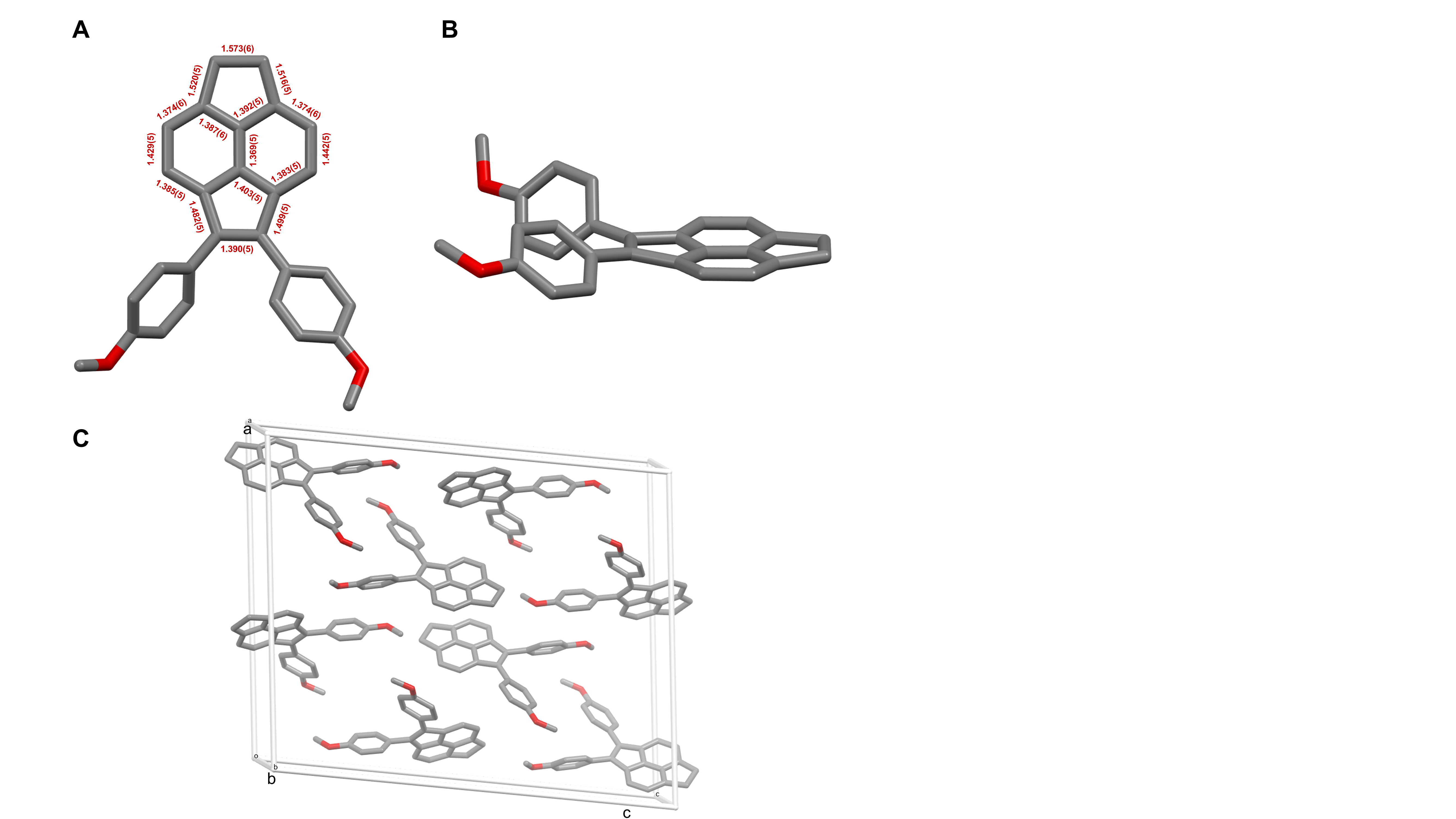


**Figure S44.** Solid state characterization of **6**. Color code: carbon (grey), oxygen (red). a) Top view with bond lengths of the core scaffold given in Å. b) Side view of the molecule. c) Visualization of the unit cell.


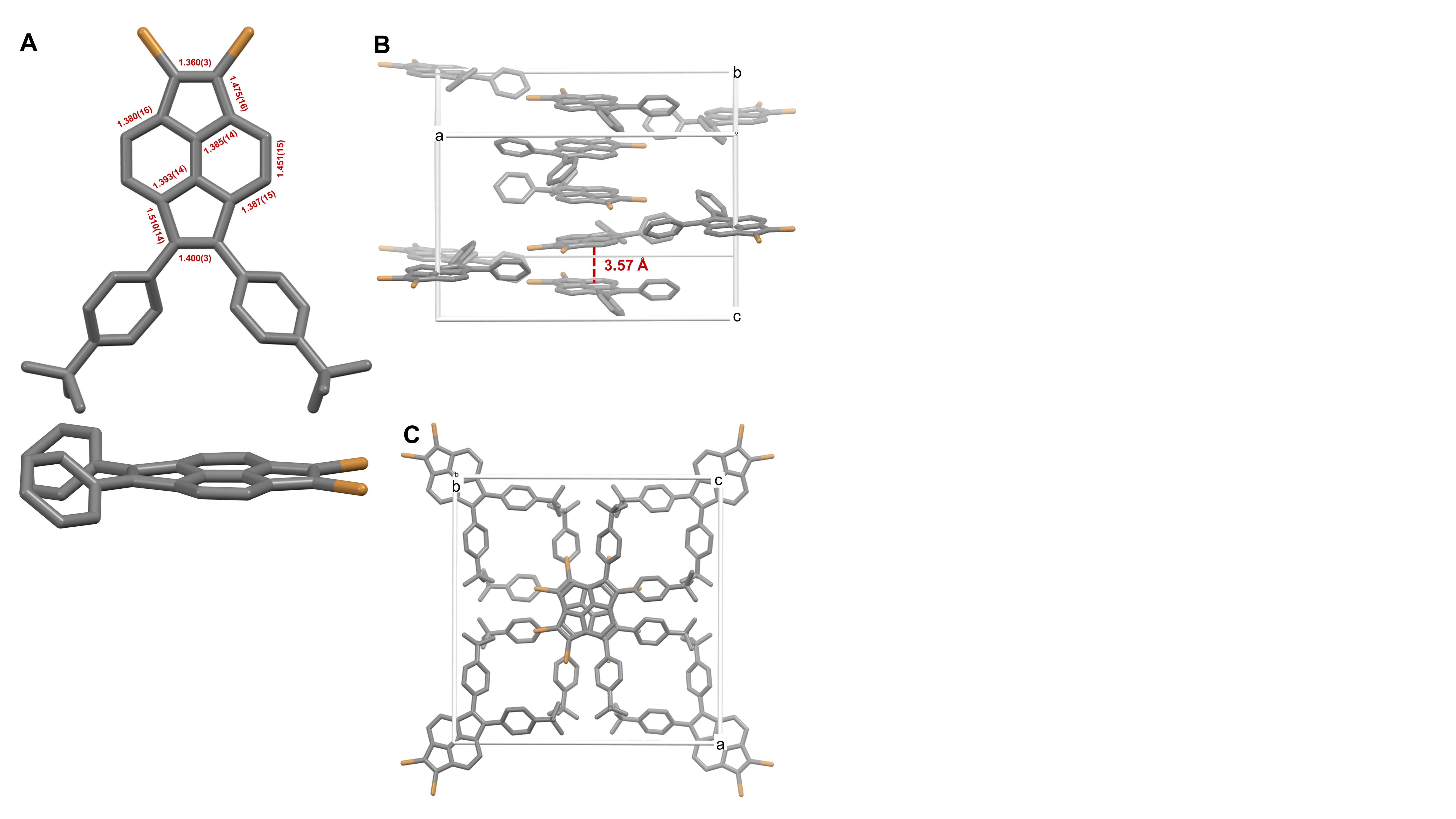


**Figure S45.** Solid state characterization of **7**. Color code: carbon (grey), bromine (brown). a) Top view with bond lengths of the core scaffold given in Å(top) and side view with removed *tert*-butyl groups (bottom). b) Side view of the unit cell. c) Top view of the unit cell.


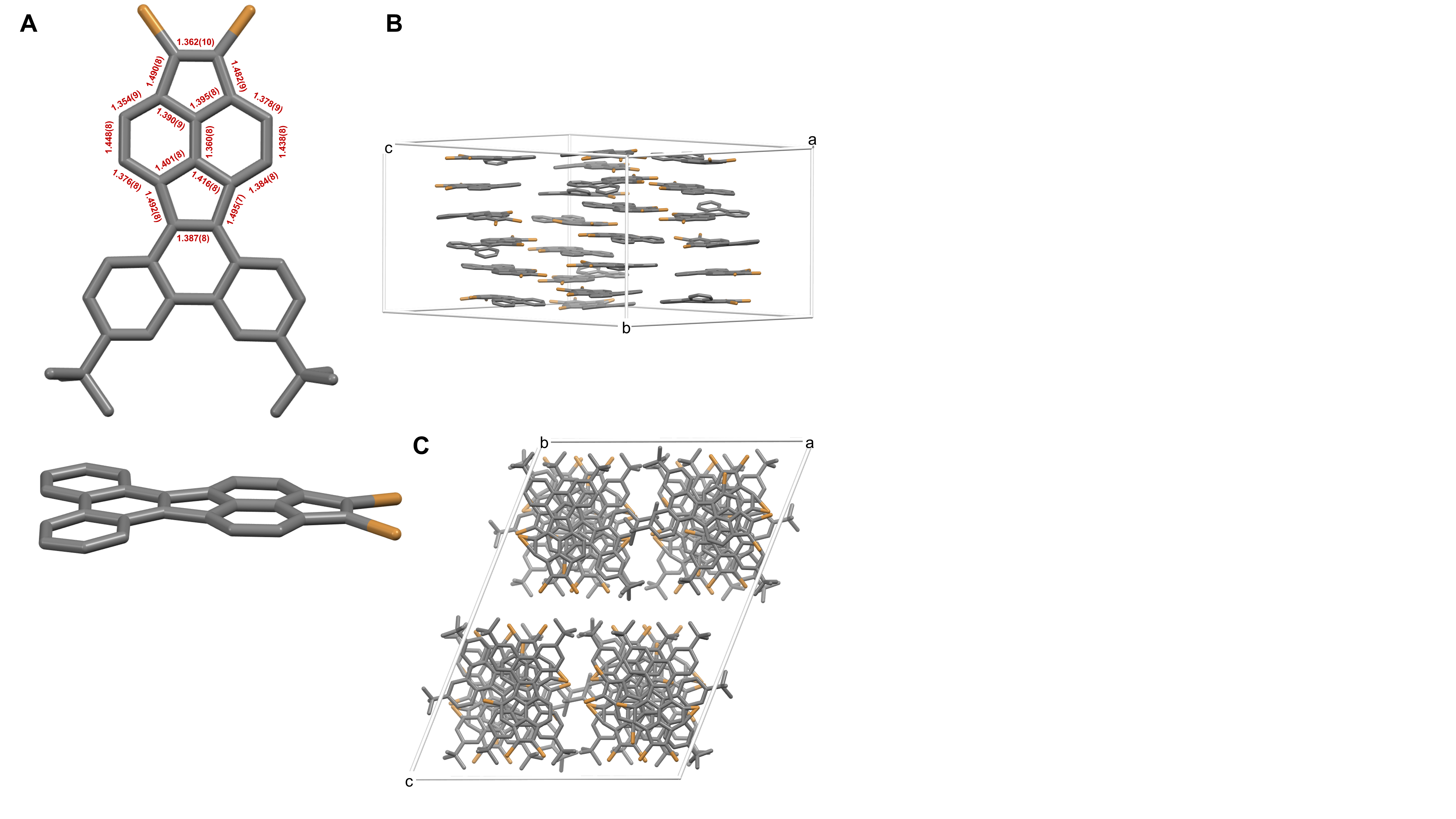


**Figure S46.** Solid state characterization of **S12**. Color code: carbon (grey), bromine (brown). a) Top view with bond lengths of the core scaffold given in Å (top). *tert*-Butyl groups removed in the side view (bottom). b) Visualization of the unit cell. c) Top view of the unit cell.


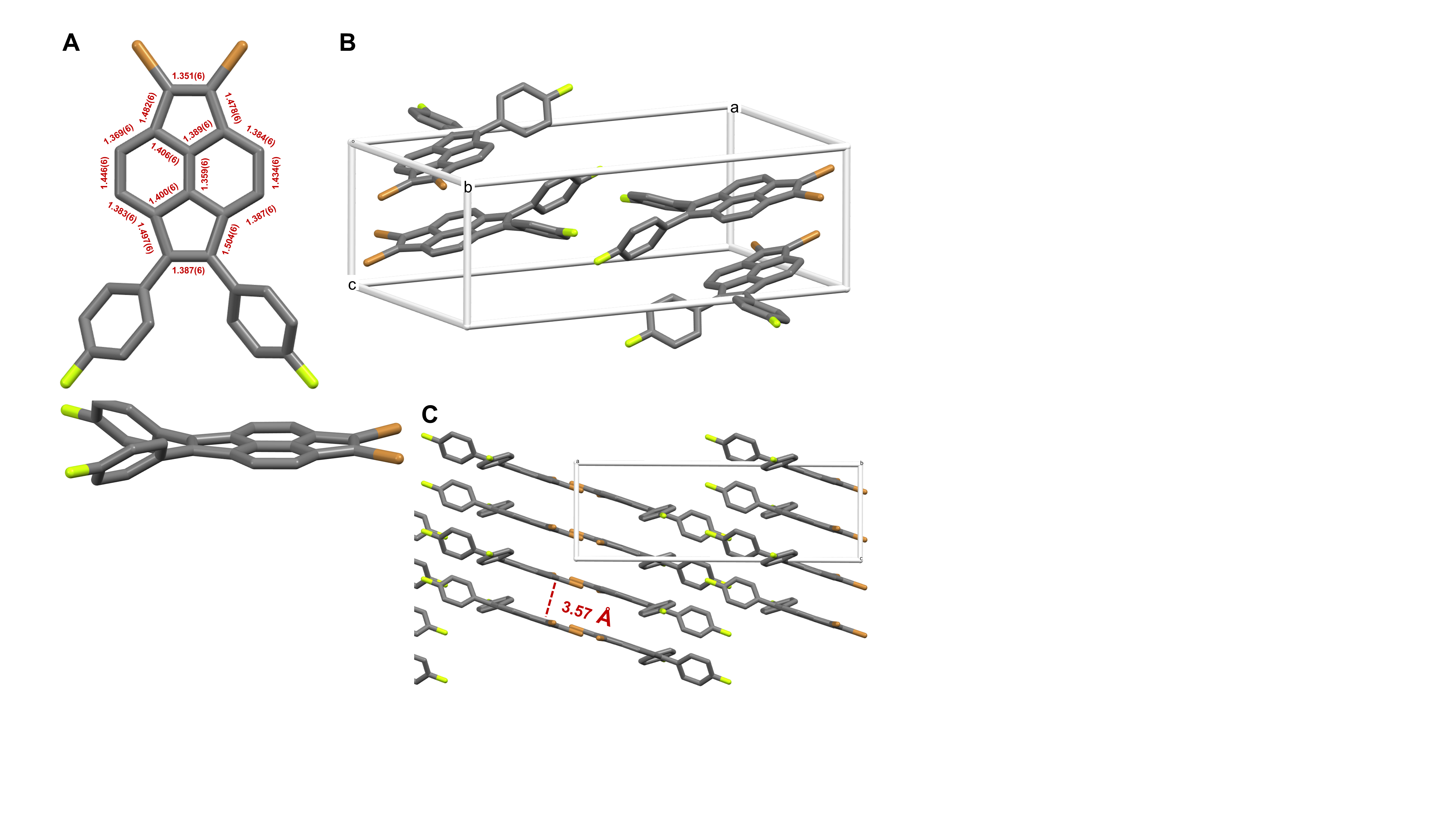


**Figure S47.** Solid state characterization of **8**. Color code: carbon (grey), bromine (brown), fluorine (yellow).
a) Top view with bond lengths of the core scaffold given in Å (Top). Side view of structure (bottom). b) Visualization of the unit cell. c) Molecular arrangement of **8** in the solid state.


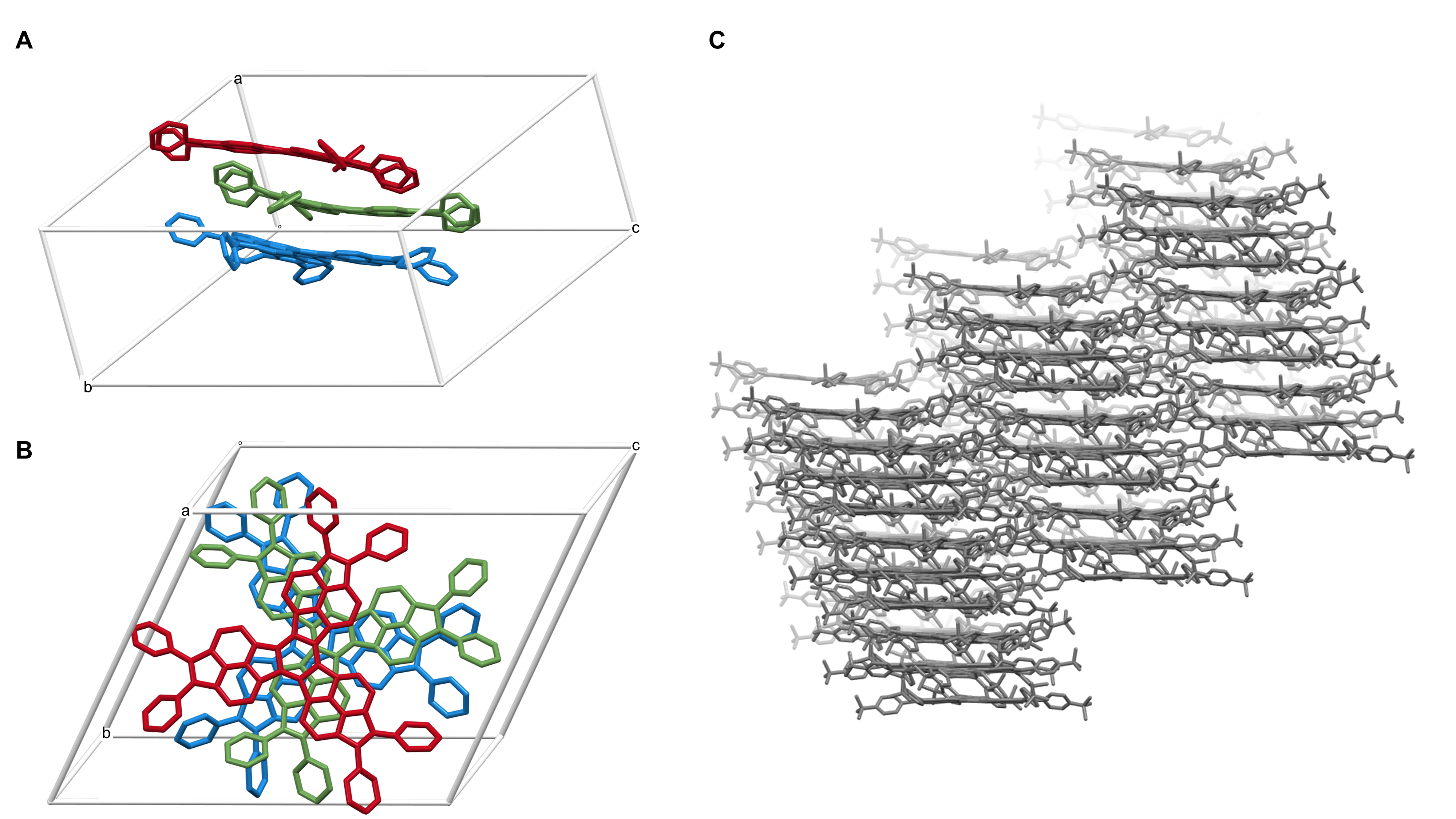


**Figure S48.** Solid state characterization of **D‑Cp‑*^t^*Bu**. Color code: carbon (grey). a) Side view of the unit cell with three stacked molecules. *tert*-Butyl groups removed for clarity. b) Top view of the unit cell with three stacked molecules. *tert*-Butyl groups removed for clarity. c) Stacks of **D‑Cp‑*^t^*Bu** in the solid state.


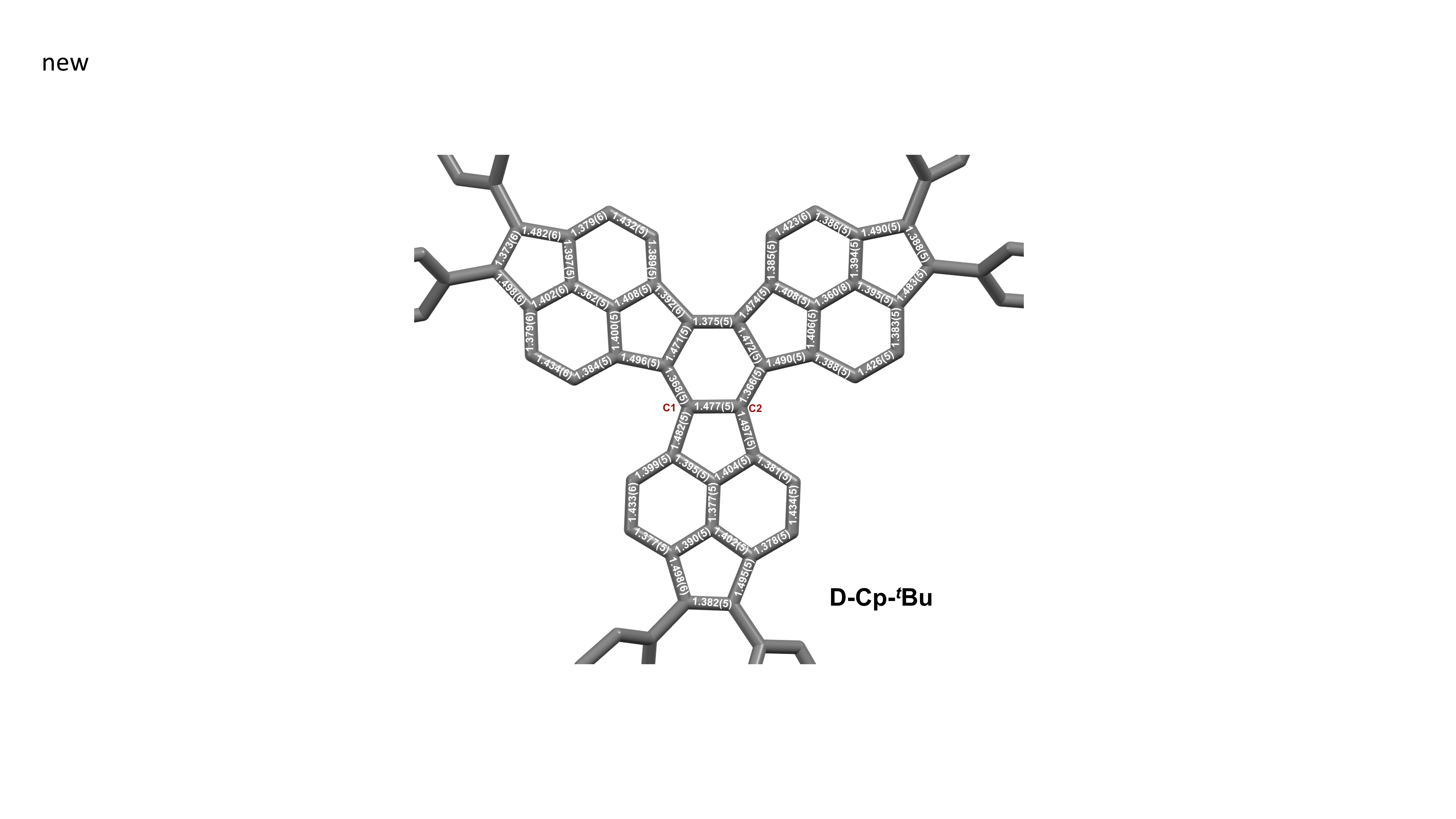


**Figure S49.** Carbon-carbon bond lengths in **D‑Cp‑*^t^*Bu** extracted from the X-ray crystal structure. Color code: carbon (grey). Values are averaged for the three independent molecules of the unit cell. Values given in Å with standard deviation of the averaged values as calculated below.

The mean bond lengths displayed in Figure S46 were calculated as the weighted mean bond lengths according to the formula $\overline{x}=\frac{\sum\frac{x_{i}}{\sigma_{i}^{2}}}{\sum\frac{1}{\sigma_{i}^{2}}}$ with $\overline{x}$ as the weighted mean bond lengths, $x_{i}$ as the bond lengths of the respective independent molecules and $\sigma_{i}$ as their respective standard deviation. The standard deviation of the mean values $\sigma_{\bar{x}}$ given in parenthesis was calculated as $\sigma_{\bar{x}}=\sqrt{\frac{1}{\sum\frac{1}{\sigma_{i}^{2}}}}$.


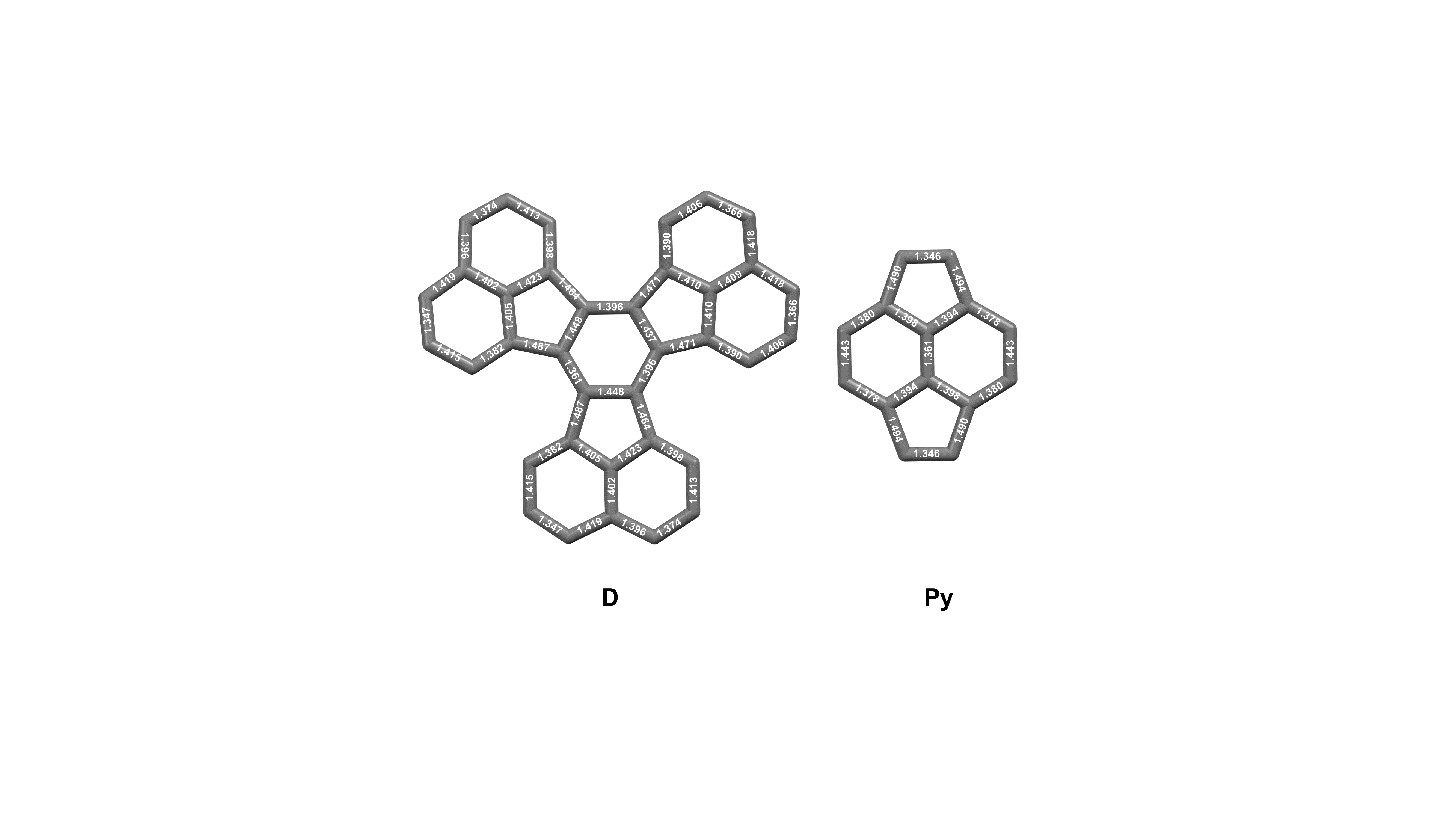


**Figure S50.** Carbon-carbon bond lengths in **D**^[25]^ and pyracylene (**Py**)^[26]^ extracted from the respective X-ray crystal structure available in the literature. Color code: carbon (grey). Values given in Å.


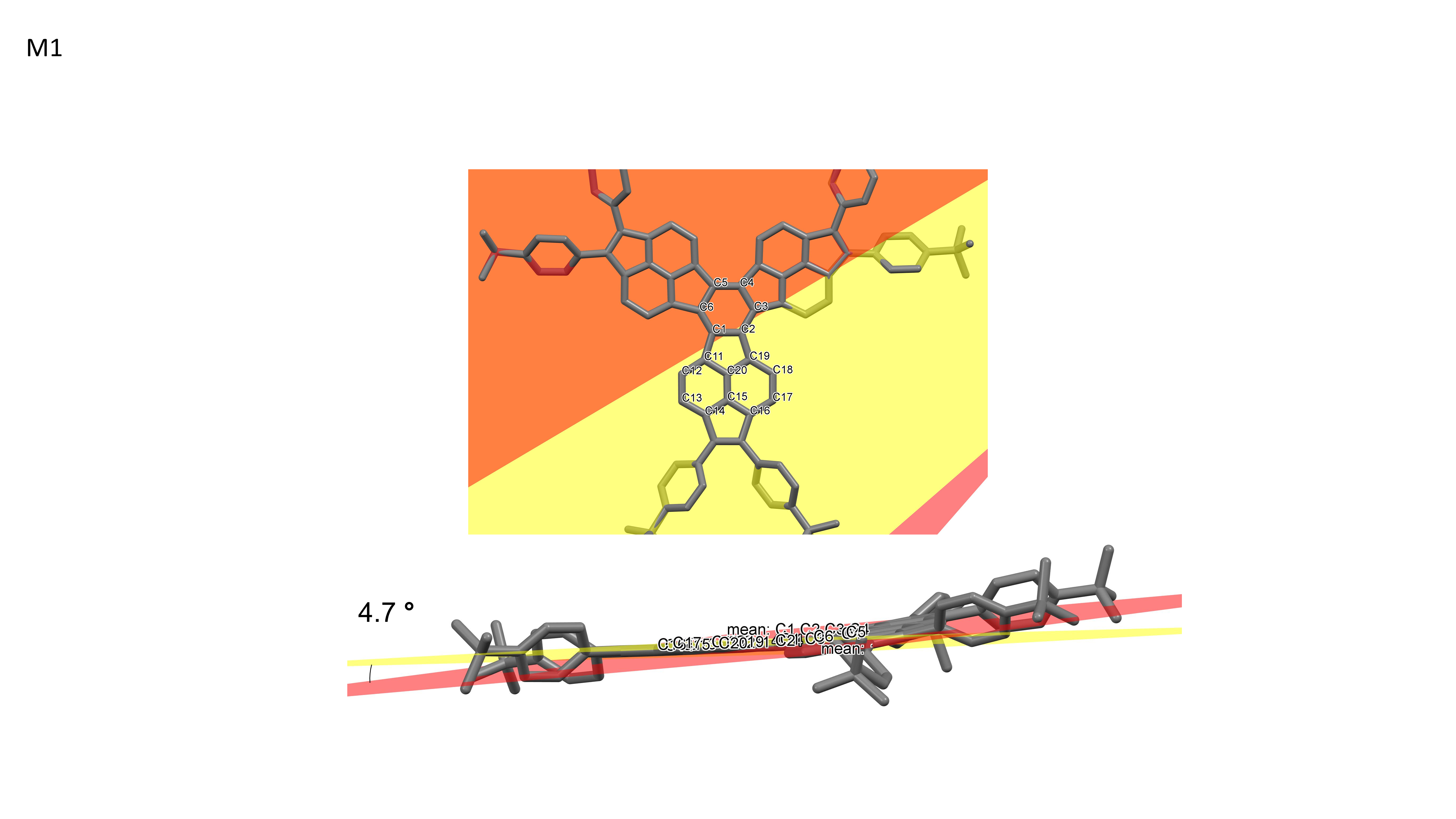


**Figure S51.** Top (top) and side view (bottom) on calculation of the angle between the central hexagonal ring and the naphthalene moiety containing C11 of independent molecule 1 in the X-ray crystal structure.


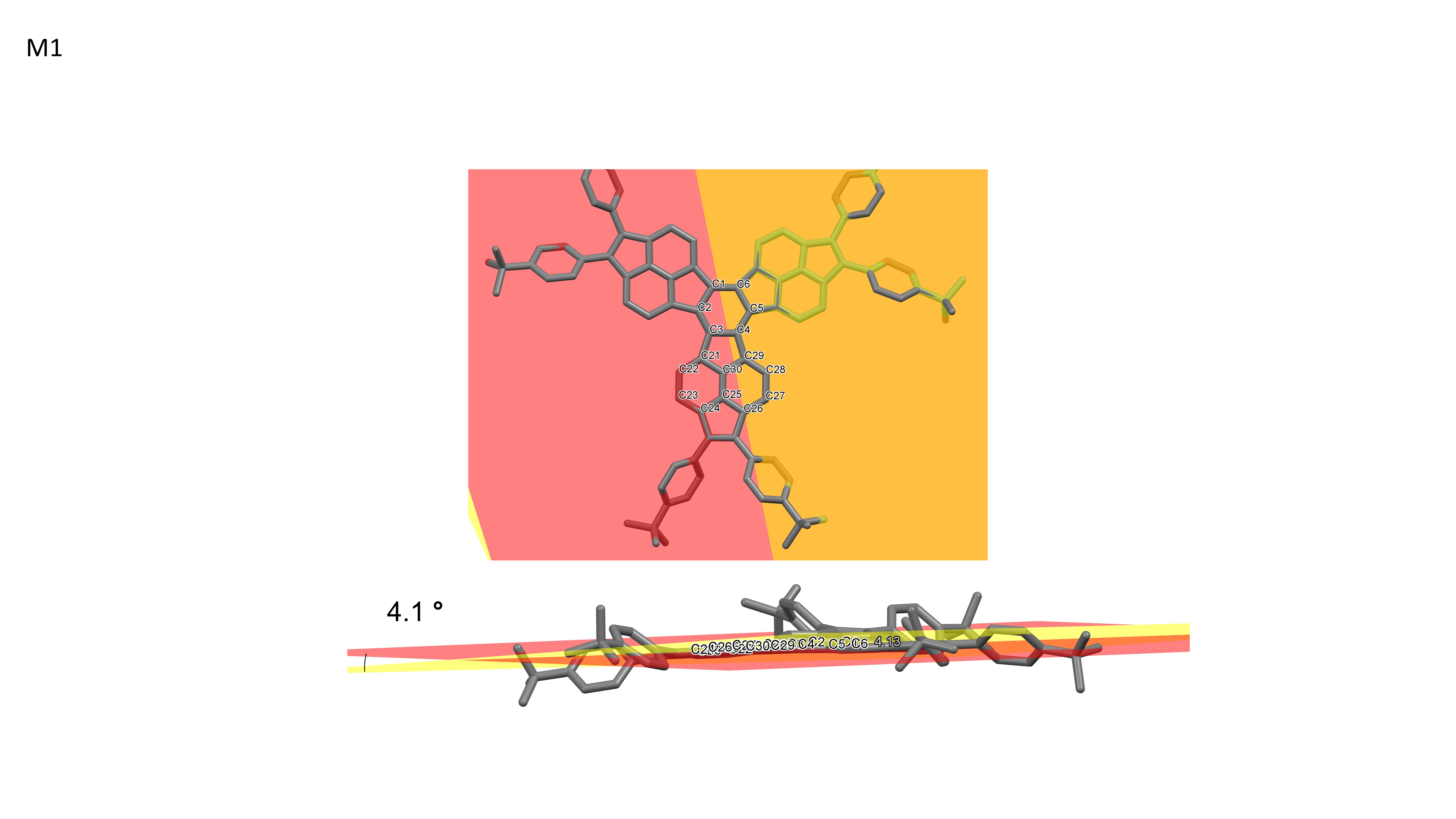


**Figure S52.** Top (top) and side view (bottom) on calculation of the angle between the central hexagonal ring and the naphthalene moiety containing C21 of independent molecule 1 in the X-ray crystal structure.


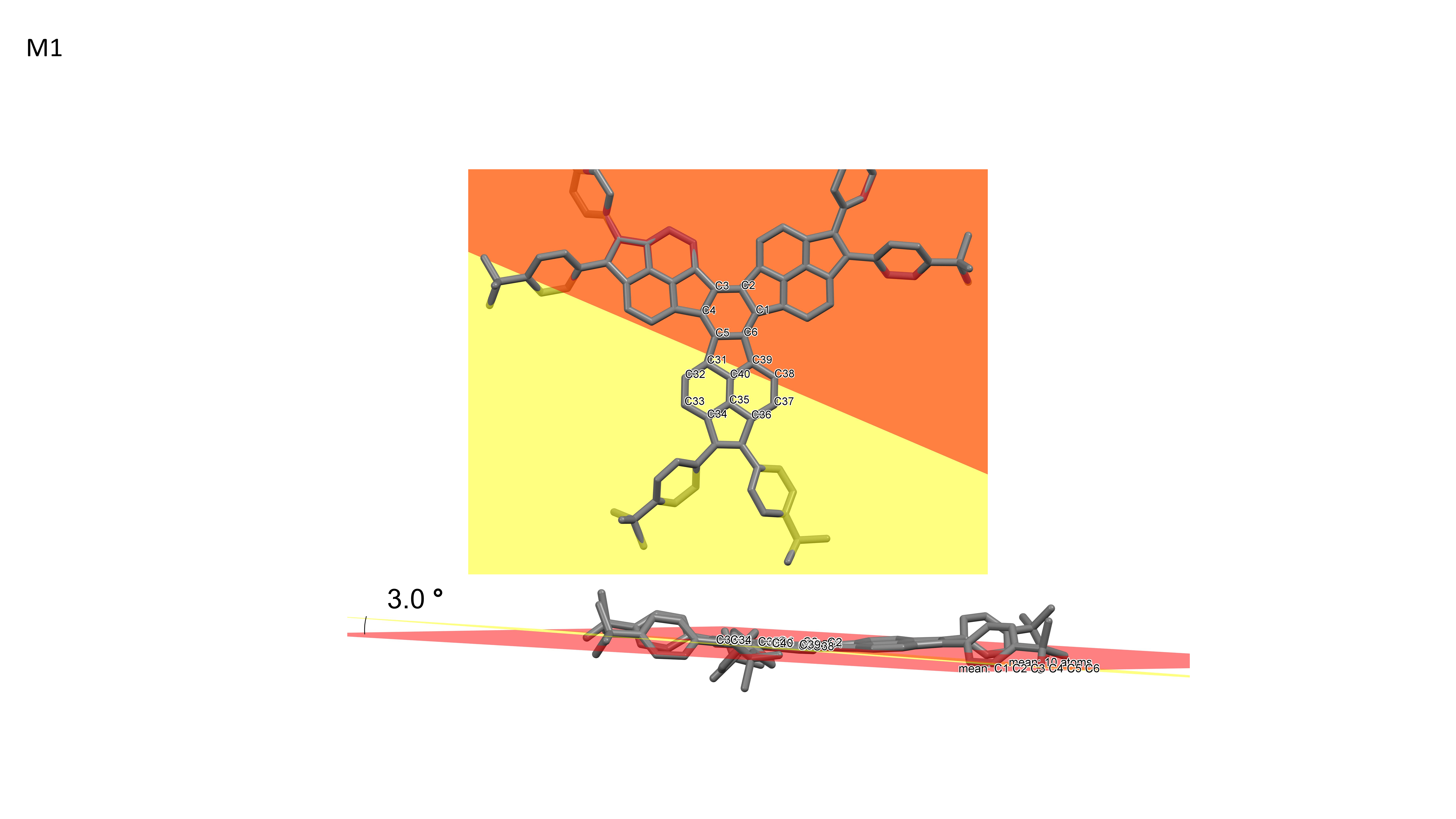


**Figure S53.** Top (top) and side view (bottom) on calculation of the angle between the central hexagonal ring and the naphthalene moiety containing C31 of independent molecule 1 in the X-ray crystal structure.


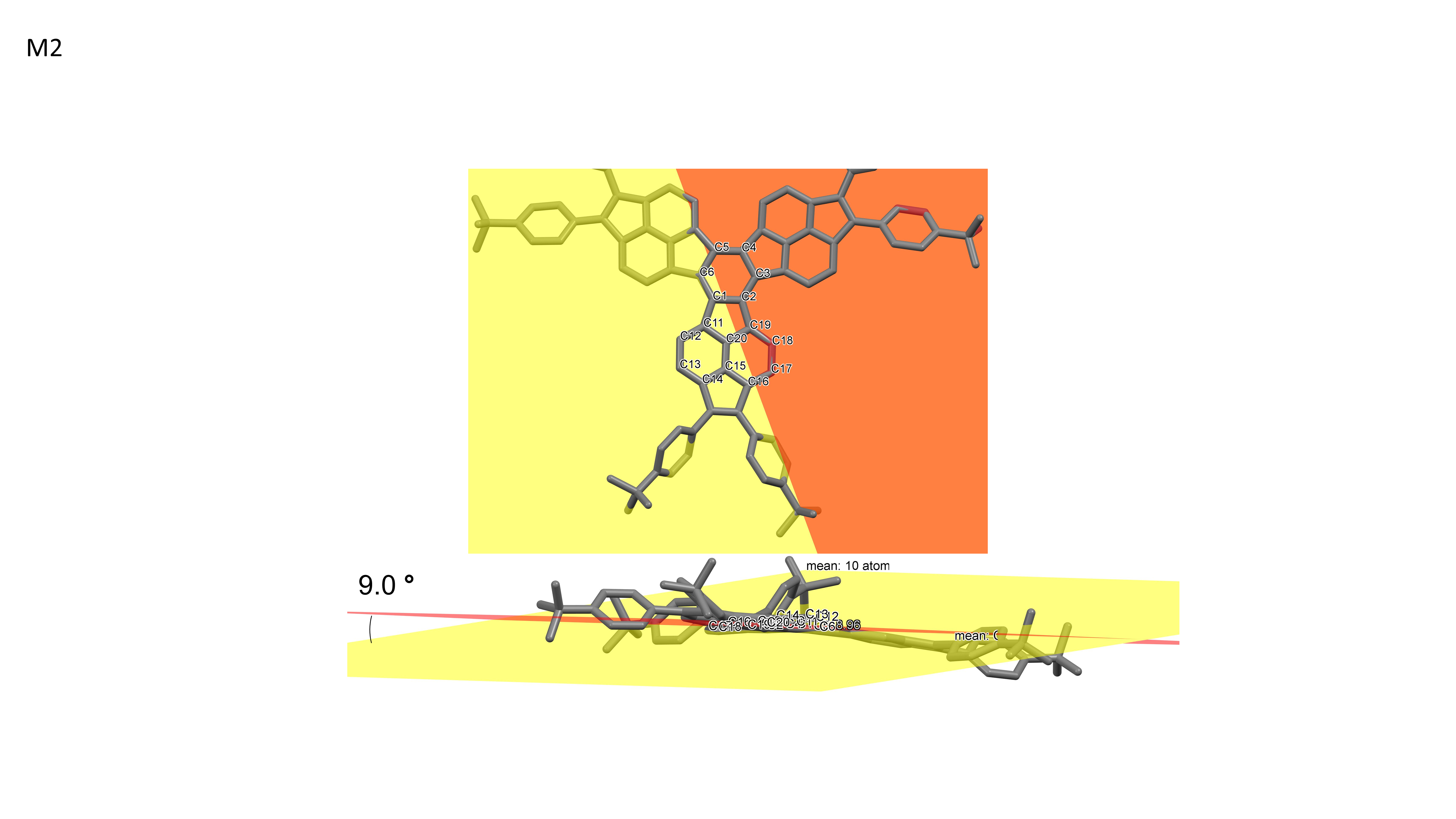


**Figure S54.** Top (top) and side view (bottom) on calculation of the angle between the central hexagonal ring and the naphthalene moiety containing C11 of independent molecule 2 in the X-ray crystal structure.


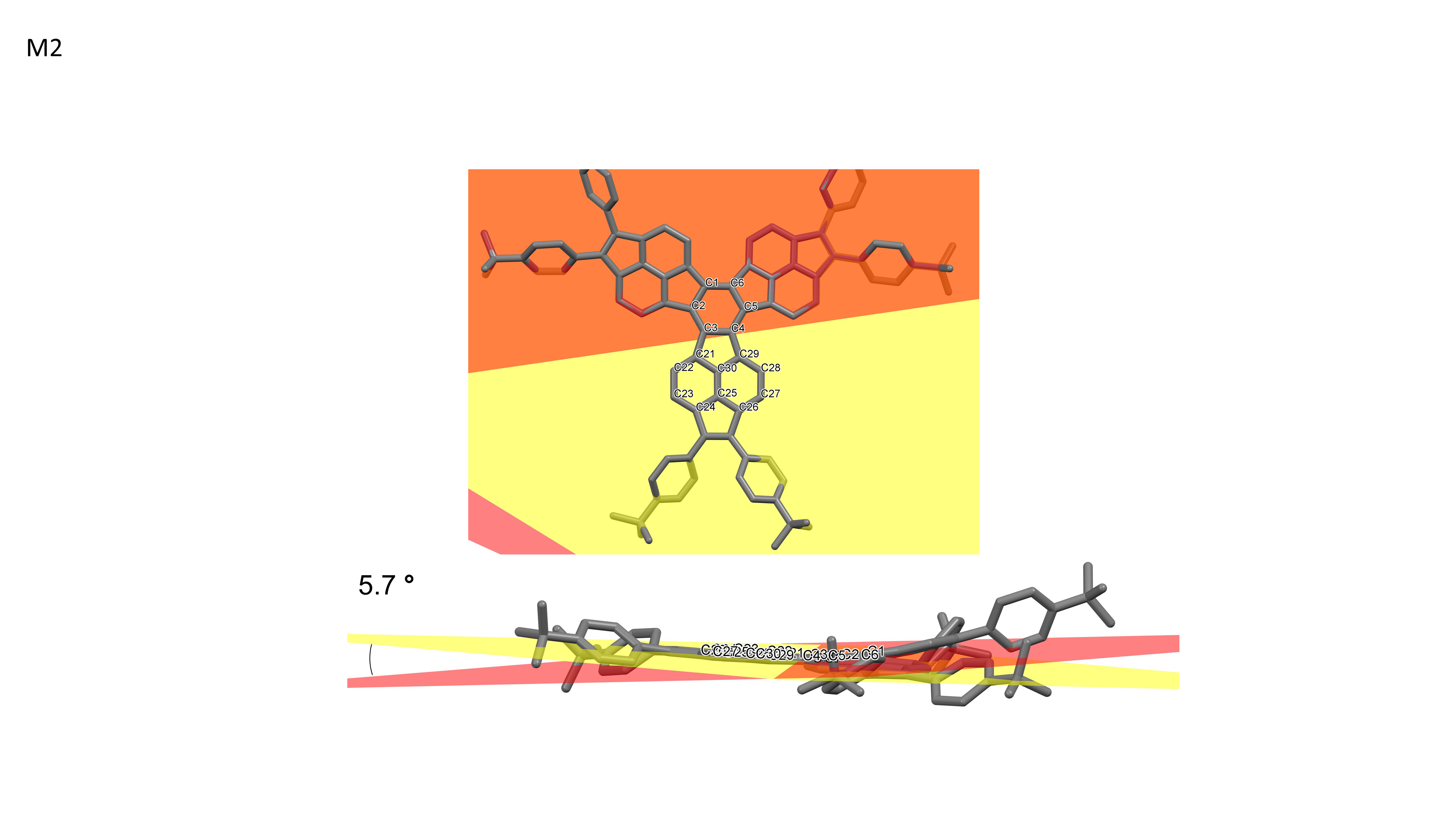


**Figure S55.** Top (top) and side view (bottom) on calculation of the angle between the central hexagonal ring and the naphthalene moiety containing C21 of independent molecule 2 in the X-ray crystal structure.


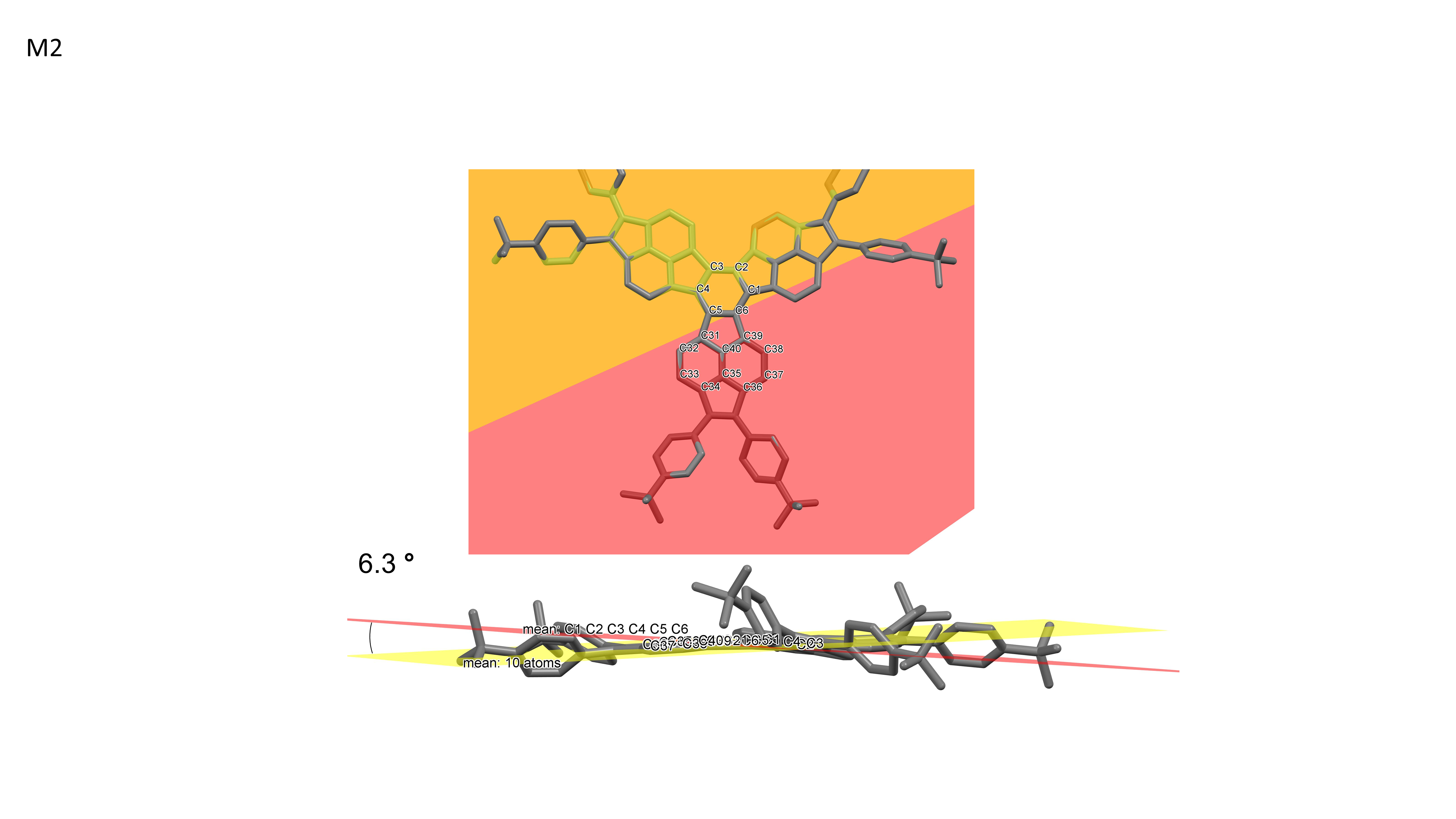


**Figure S56.** Top (top) and side view (bottom) on calculation of the angle between the central hexagonal ring and the naphthalene moiety containing C31 of independent molecule 2 in the X-ray crystal structure.


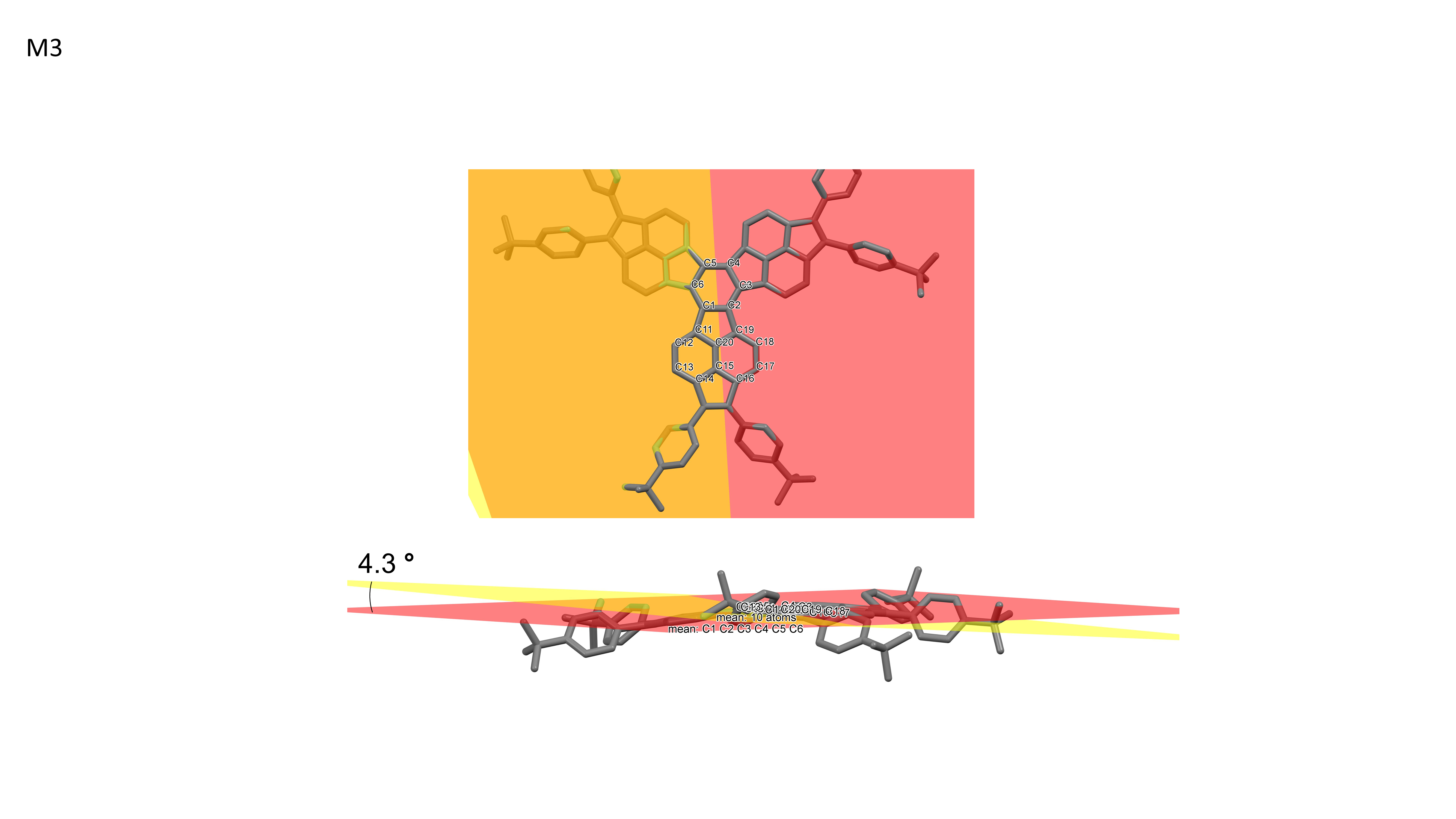


**Figure S57.** Top (top) and side view (bottom) on calculation of the angle between the central hexagonal ring and the naphthalene moiety containing C11 of independent molecule 3 in the X-ray crystal structure.


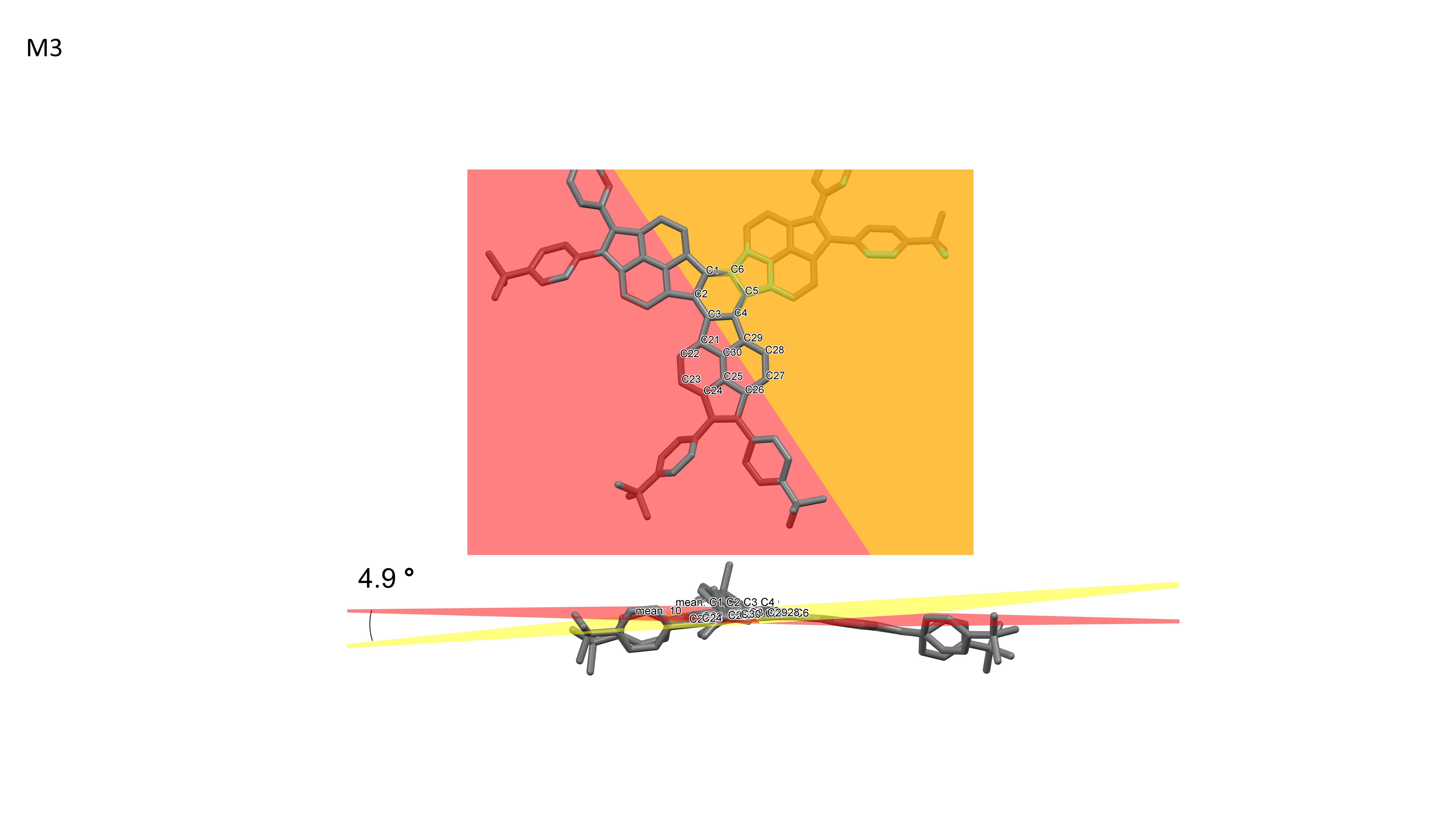


**Figure S58.** Top (top) and side view (bottom) on calculation of the angle between the central hexagonal ring and the naphthalene moiety containing C21 of independent molecule 3 in the X-ray crystal structure.


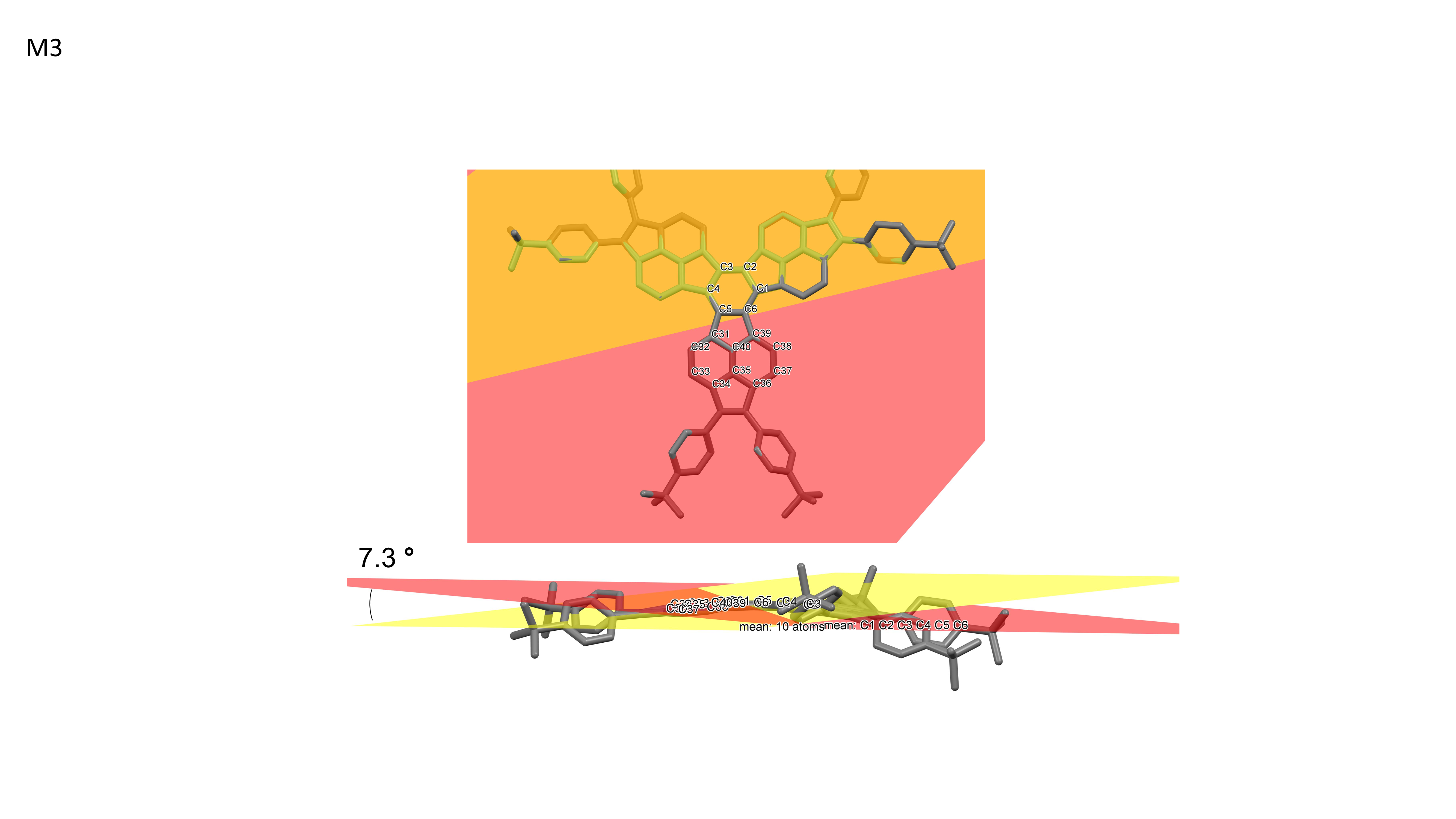


**Figure S59.** Top (top) and side view (bottom) on calculation of the angle between the central hexagonal ring and the naphthalene moiety containing C31 of independent molecule 3 in the X-ray crystal structure.


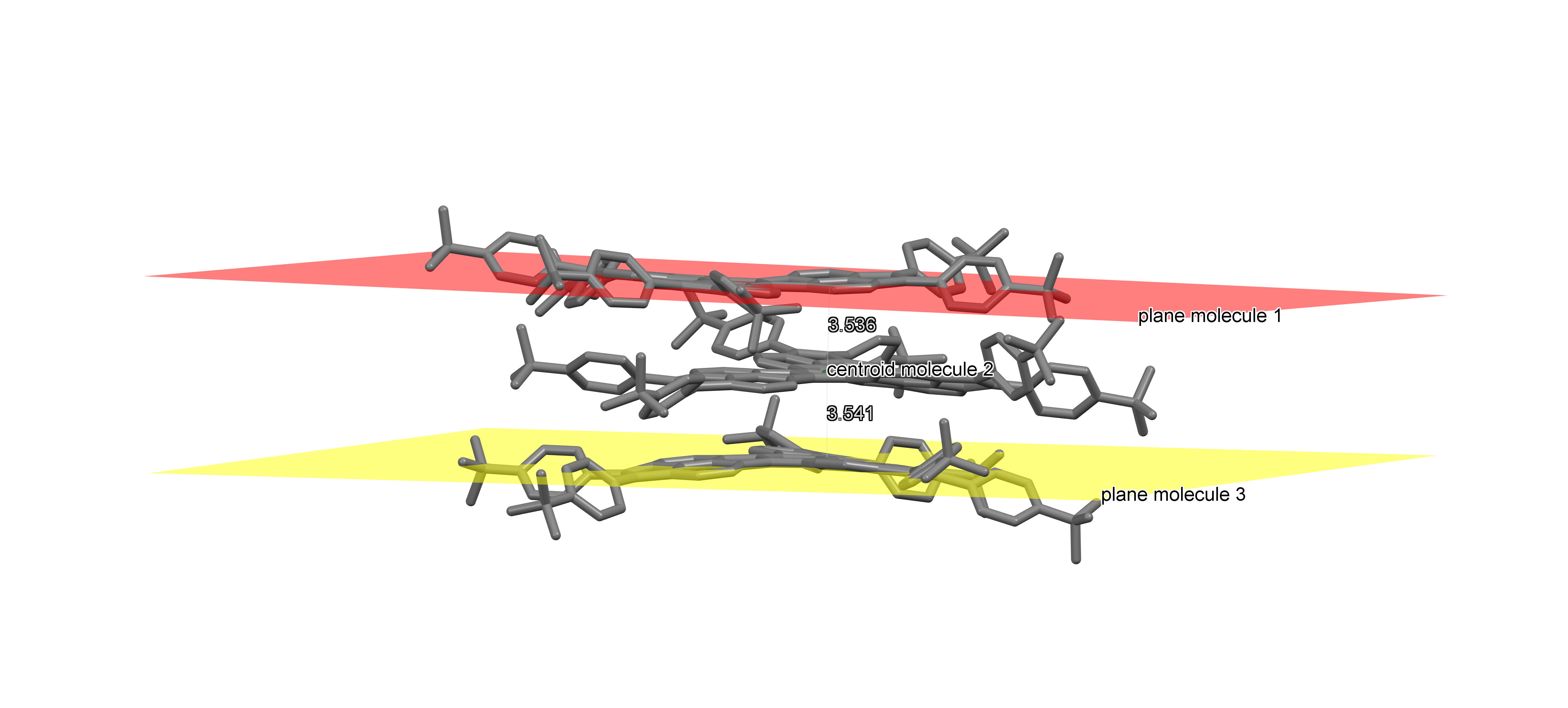


**Figure S60.** Calculation of ‑ stacking distances in the unit cell of **D‑Cp‑*^t^*Bu**. The planes for molecules 1 and 3 correspond to the mean planes considering all carbon atoms of the cyclopentannulateddecacyclene core. The centroid in molecule 2 was placed in the middle of the molecule considering the carbon atoms of the cyclopentannulateddecacyclene core.

1. UV/Vis Absorption Spectroscopy Data

**Figure S61.** UV/Vis absorption spectra of 1,2-dihydropyracylene precursors **4**, **5** and **6** in CH_2_Cl_2_ at rt.

**Figure S62.** UV/Vis absorption spectra of pyracylene precursors **7**, **8,** **9** and **S12** in CH_2_Cl_2_ at rt.

**Figure S63.** UV/Vis absorption (solid line) and emission (dashed line) spectrum of **D** in CH_2_Cl_2_ at rt. (excitation wavelength 378 nm).

**Figure S64.** UV/Vis absorption spectrum of **D‑Cp‑*^t^*Bu** in CH_2_Cl_2_ at rt.


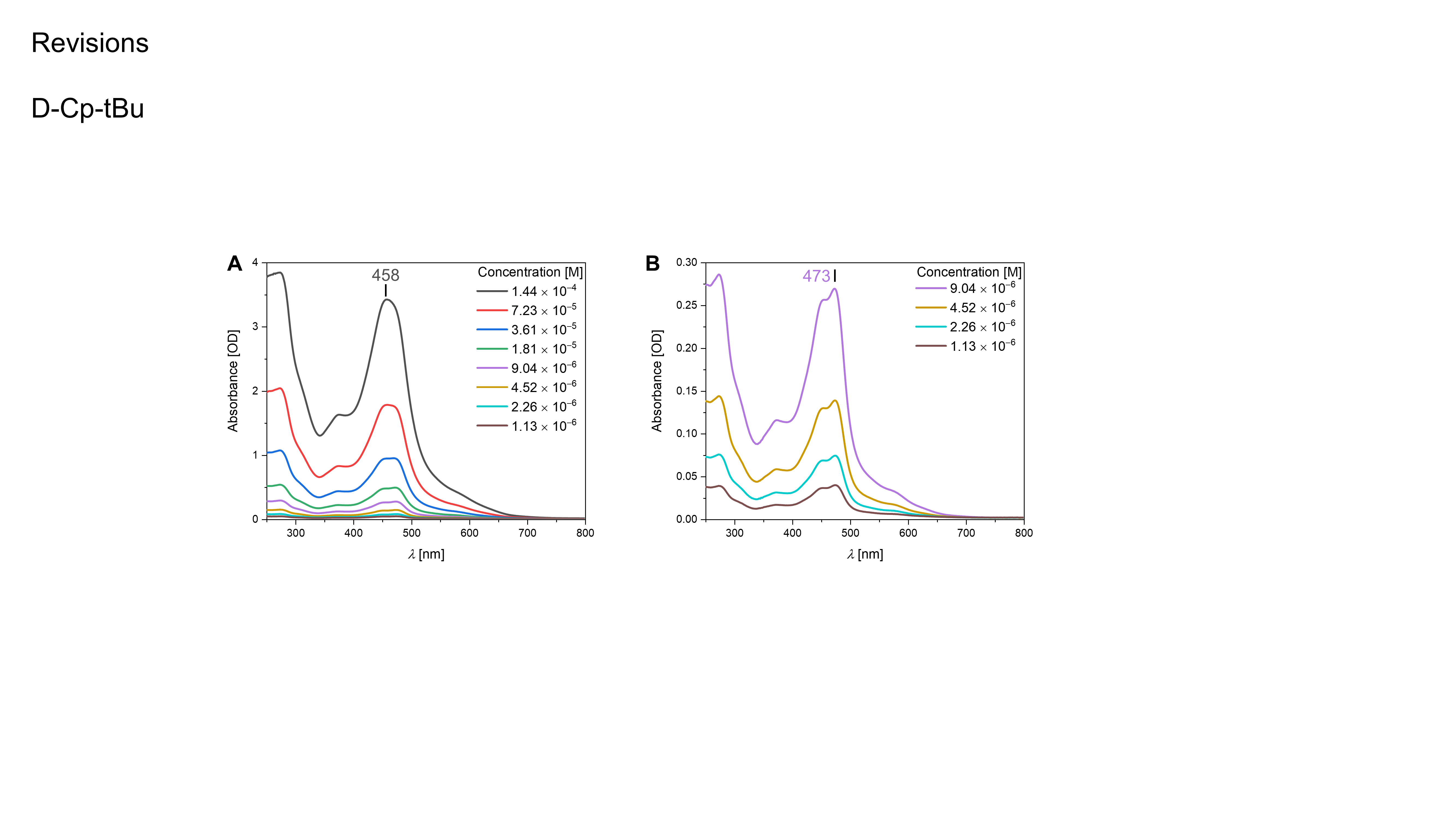


**Figure S65.** UV/Vis absorption spectrum of **D‑Cp‑*^t^*Bu** in CH_2_Cl_2_ at rt at concentrations up to 1.44 × 10^–4^ m (**A**) and up to 9.04 × 10^–6^ m (**B**).

**Figure S66.** UV/Vis absorption spectrum of **D‑Cp‑*^t^*Bu‑Cyc** in CH_2_Cl_2_ at rt.


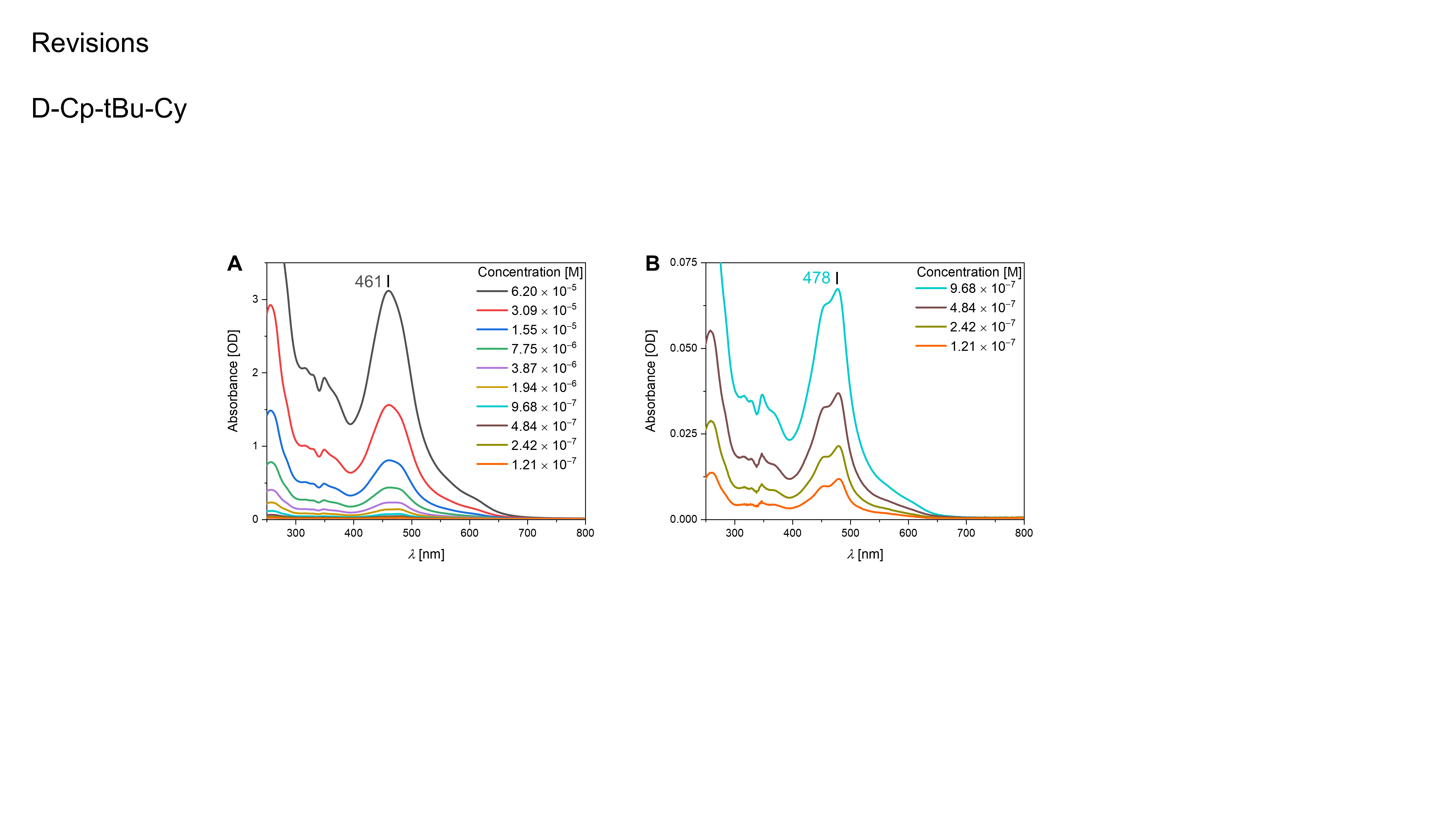


**Figure S67.** UV/Vis absorption spectrum of **D‑Cp‑*^t^*Bu‑Cyc** in CH_2_Cl_2_ at rt at concentrations up to 6.20 × 10^–5^ m (**A**) and up to
9.68 × 10^–7^ m (**B**).

**Figure S68.** UV/Vis absorption spectrum of **D-Cp-OMe** in CH_2_Cl_2_ at rt.


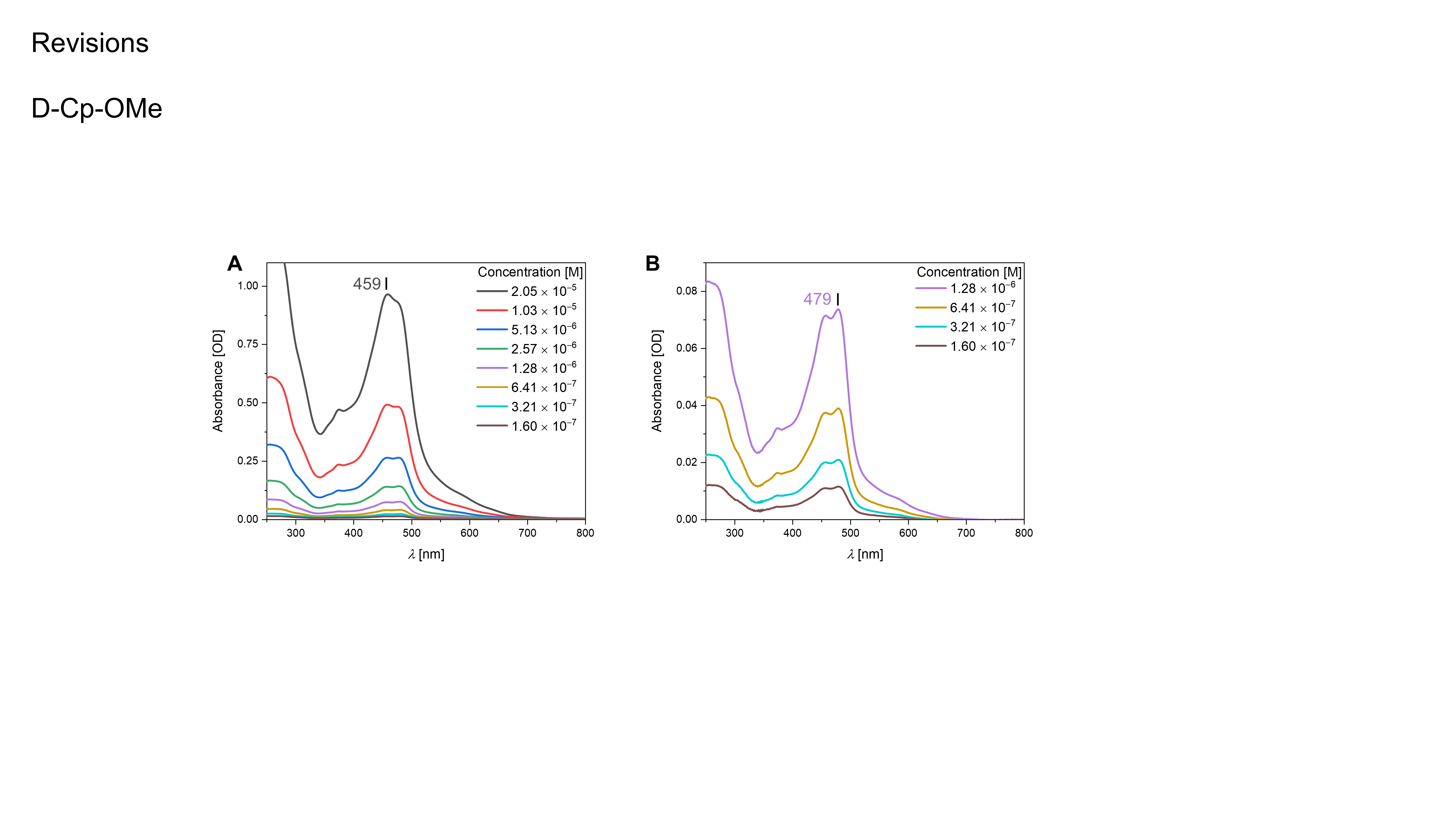


**Figure S69.** UV/Vis absorption spectrum of **D-Cp-OMe** in CH_2_Cl_2_ at rt at concentrations up to 2.05 × 10^–5^ m (**A**) and up to
1.28 × 10^–6^ m (**B**).

**Figure S70.** UV/Vis absorption spectrum of **D‑Cp‑F** in CH_2_Cl_2_ at rt.


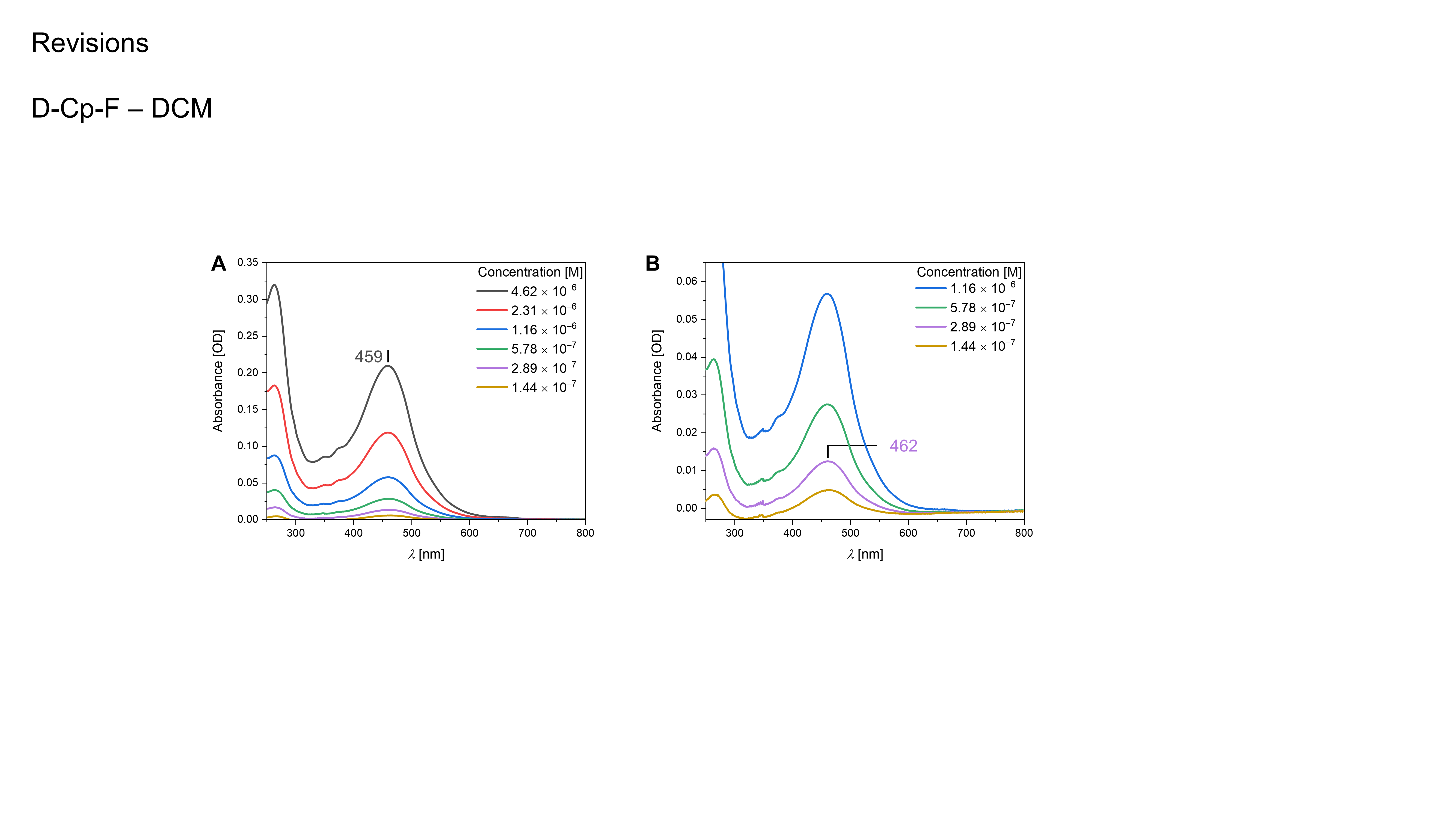


**Figure S71.** UV/Vis absorption spectrum of **D‑Cp‑F** in CH_2_Cl_2_ at rt at concentrations up to 4.62 × 10^–6^ m (**A**) and up to 1.16 × 10^–6^ m (**B**).


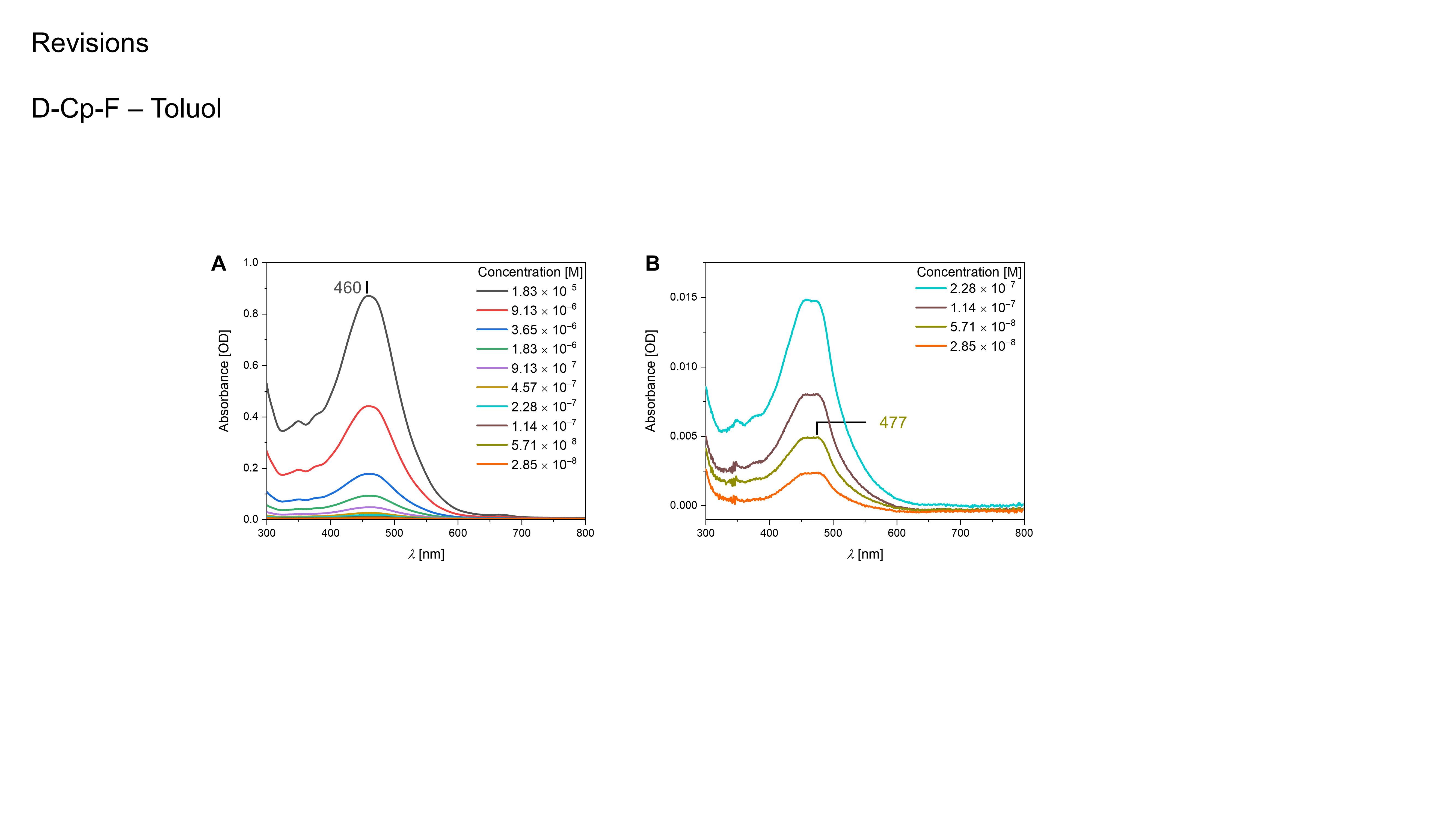


**Figure S72.** UV/Vis absorption spectrum of **D‑Cp‑F** in toluene at rt at concentrations up to 1.83 × 10^–5^ m (**A**) and up to 2.28 × 10^–7^ m (**B**).

**Table S8.** Summary of the optical properties of the precursors **4**–**9** and **S12**.

| PAH | *λ*_max_ [nm]^[a]^ | *ε* (*λ*_max_)[m^–1^cm^–1^] | | *λ*_on_ [nm]^[b]^ | E_g, opt_ [eV]^[c]^ | |
| --- | --- | --- | --- | --- | --- | --- |
| **4** | 431 | 3100 | 518 | | | 2.39 |
| **5** | 421 | 2400 | 498 | | | 2.49 |
| **6** | 446 | 3200 | 532 | | | 2.33 |
| **7** | 386 | 16900 | 506 | | | 2.45 |
| **8** | 382 | 15500 | 498 | | | 2.49 |
| **9** | 377 | 21400 | 528 | | | 2.35 |
| **S12** | 425 | 8400 | 477 | | | 2.60 |

[a] Longest wavelength absorption peak in CH_2_Cl_2_ at rt. [b] Estimated from the onset of absorption via tangent method.^[27]^ [c] Calculated from the onset of the UV/Vis absorption using the equation E_g,opt_ = (hc) /λ_on_.

**Table S9.** Summary of the optical properties of decacyclene **D** and the series of cyclotrimers **D‑Cp‑*^t^*Bu‑Cyc**, **D‑Cp‑*^t^*Bu**, **D‑Cp‑F** and **D‑Cp‑OMe**.

| PAH | *λ*_max_ [nm]^[a]^ | *ε* (*λ*_max_)[m^–1^cm^–1^] | | *λ*_on_ [nm]^[b]^ | E_g, opt_ [eV]^[c]^ | |
| --- | --- | --- | --- | --- | --- | --- |
| **D** | 444 | 7810 | 467 | | | 2.66 |
| **D‑Cp‑*^t^*Bu‑Cyc** | 478 | 67457 | 664 | | | 1.87 |
| **D‑Cp‑*^t^*Bu** | 473 | 59982 | 653 | | | 1.90 |
| **D‑Cp‑F** | 466 | 49180 | 645 | | | 1.92 |
| **D‑Cp‑OMe** | 479 | 51681 | 660 | | | 1.88 |

[a] Longest wavelength absorption peak in CH_2_Cl_2_ at rt. [b] Estimated from the onset of absorption via tangent method.^[27]^
[c] Calculated from the onset of the UV/Vis absorption using the equation E_g,opt_ = (hc) /λ_on_.

1. Electrochemical Data

**Figure S73.** Cyclic and square-wave voltammogram of compounds **4**, **5** and **6** measured in THF at rt (scan rate of 149 mV s^-1^) and referenced against Fc/Fc^+^. Reduction potentials were obtained from SWV.

**Figure S74.** Cyclic and square-wave voltammogram of compounds **7**, **8**, **9** and **S12** measured in THF at rt (scan rate of 149 mVs^-1^) and referenced against Fc/Fc^+^. Reduction potentials were obtained from SWV.

**Figure S75.** CV (middle), SWV (top) and DPV (bottom) data of **D** measured in THF at rt (scan rate of 149 mVs^-1^) and referenced against Fc/Fc^+^. Reduction potentials were obtained from SWV.

**Figure S76.** CV (middle), SWV (top) and DPV (bottom) data of **D** measured in THF at rt (scan rate of 149 mVs^-1^) and referenced against Fc/Fc^+^. Reduction potentials were obtained from SWV.

**Figure S77.** CV (middle), SWV (top) and DPV (bottom) data of **D‑Cp‑*^t^*Bu** measured in THF at rt (scan rate of 149 mVs^-1^) and referenced against Fc/Fc^+^. Reduction potentials were obtained from SWV.

**Figure S78.** CV (middle), SWV (top) and DPV (bottom) data of **D‑Cp‑*^t^*Bu** measured in THF at rt (scan rate of 149 mVs^-1^) and referenced against Fc/Fc^+^. Reduction potentials were obtained from SWV.

**Figure S79.** CV (middle), SWV (top) and DPV (bottom) data of **D‑Cp-*^t^*Bu‑Cyc** measured in THF at rt (scan rate of 149 mVs^-1^) and referenced against Fc/Fc^+^. Reduction potentials were obtained from SWV. * marks signal corresponding to the Fc/Fc^+^ redox couple.

**Figure S80.** CV (middle), SWV (top) and DPV (bottom) data of **D‑Cp‑*^t^*Bu‑Cyc** measured in THF at rt (scan rate of 149 mVs^-1^) and referenced against Fc/Fc^+^. Reduction potentials were obtained from SWV.

**Figure S81.** CV (middle), SWV (top) and DPV (bottom) data of **D‑Cp‑OMe** measured in THF at rt (scan rate of 149 mVs^-1^) and referenced against Fc/Fc^+^. Reduction v potentials were obtained from SWV.

**Figure S82.** CV (middle), SWV (top) and DPV (bottom) data of **D‑Cp-OMe** measured in THF at rt (scan rate of 149 mVs^-1^) and referenced against Fc/Fc^+^. Reduction potentials were obtained from SWV.

**Figure S83.** CV (middle), SWV (top) and DPV (bottom) data of **D‑Cp-F** measured in THF at rt (scan rate of 149 mVs^-1^) and referenced against Fc/Fc^+^. Reduction potentials were obtained from SWV.

**Figure S84.** CV (middle), SWV (top) and DPV (bottom) dataof **D‑Cp-F** measured in THF at rt (scan rate of 149 mVs^-1^) and referenced against Fc/Fc^+^. Reduction potentials were obtained from SWV.

**Table S10.** Summary of the electrochemical properties of the precursors **4**–**9** and **S12**.

| PAH | E_red,1_[V]^[a]^ | E_red,2_[V]^[b]^ | E_HOMO, CV_[eV] ^[c]^ | E_LUMO, CV_[eV] ^[d]^ | E_g, opt_ [eV] ^[e]^ |
| --- | --- | --- | --- | --- | --- |
| **4** | –2.20 | –2.82 | –5.29 | –2,90 | 2.39 |
| **5** | –2.15 | –2.75 | –5.44 | –2.95 | 2.49 |
| **6** | –2.23 | –2.82 | –5.20 | –2.87 | 2.33 |
| **7** | –1.34 | –1.78 | –6.21 | –3.76 | 2.45 |
| **8** | –1.37 | –1.82 | –6.22 | –3.73 | 2.49 |
| **9** | –1.37 | –1.82 | –6.08 | –3.73 | 2.35 |
| **S12** | –1.34 | –1.78 | –6.36 | –3.76 | 2.60 |

[a] First reduction potentials from SWV maximum in THF against Fc/Fc^+^ as internal standard at rt with *^n^*Bu_4_NPF_6_ as supporting electrolyte at a scan rate of
149 mVs^–1^. [b] Second reduction potentials obtained from SWV maximum under the same conditions as in [a]. [c] E_HOMO_ = E_LUMO_ – E_g,opt_.
[d] E_LUMO_ = – (5.1 eV + E_red,1_).^[28]^ [e] Calculated from the onset of absorption using the equation E_g,opt_ = (hc) /*λ*_on_.

**Table S11.** Summary of the electrochemical properties of the series of cyclotrimers **D‑Cp‑*^t^*Bu‑Cyc**, **D‑Cp‑*^t^*Bu**, **D‑Cp‑F** and **D‑Cp‑OMe**.

| PAH | E_red,1_ / E_red,2_ / E_red,3_ / E_red,4_ / E_red,5_ /E_red,6_ ^[a]^ [V] | E_HOMO, CV_^[b]^ [eV] | E_LUMO, CV_^[c]^ [eV] | E_g, opt_ [eV]^[d]^ |
| --- | --- | --- | --- | --- |
| **D‑Cp‑*^t^*Bu‑Cyc** | –1.56 / –1.70 / –1.90 / –2.26 / –2.57 / –2.86 | –5.34 | –3.54 | 1.80 |
| **D‑Cp‑*^t^*Bu** | –1.50 / –1.70 / –1.97 / –2.29 / –2.58 | –5.47 | –3.60 | 1.87 |
| **D‑Cp‑F** | –1.45 / –1.66 / (–1.82) / –2.25 / –2.51 / –2.77 | –5.47 | –3.65 | 1.82 |
| **D‑Cp‑OMe** | –1.52 / –1.74 / –2.00 / –2.34 / –2.62 | –5.40 | –3.58 | 1.82 |

[a] All reduction potentials from square wave voltammetry maxima in THF at rt with *^n^*Bu_4_NPF_6_ as supporting electrolyte against Fc/Fc^+^ as internal standard at
149 mVs^–1^. Values given in volt [V].[b] E_HOMO_ = E_LUMO_ – E_g,opt_. Values given in electron volt [eV].[c] E_LUMO_ = – (5.1 eV + *E*_red,1_).^[28]^ Values given in electron volt [eV].[d] Calculated from the onset of absorption using equation E_g,opt_ = (hc) /*λ*_on_.

1. Frontier Molecular Orbitals


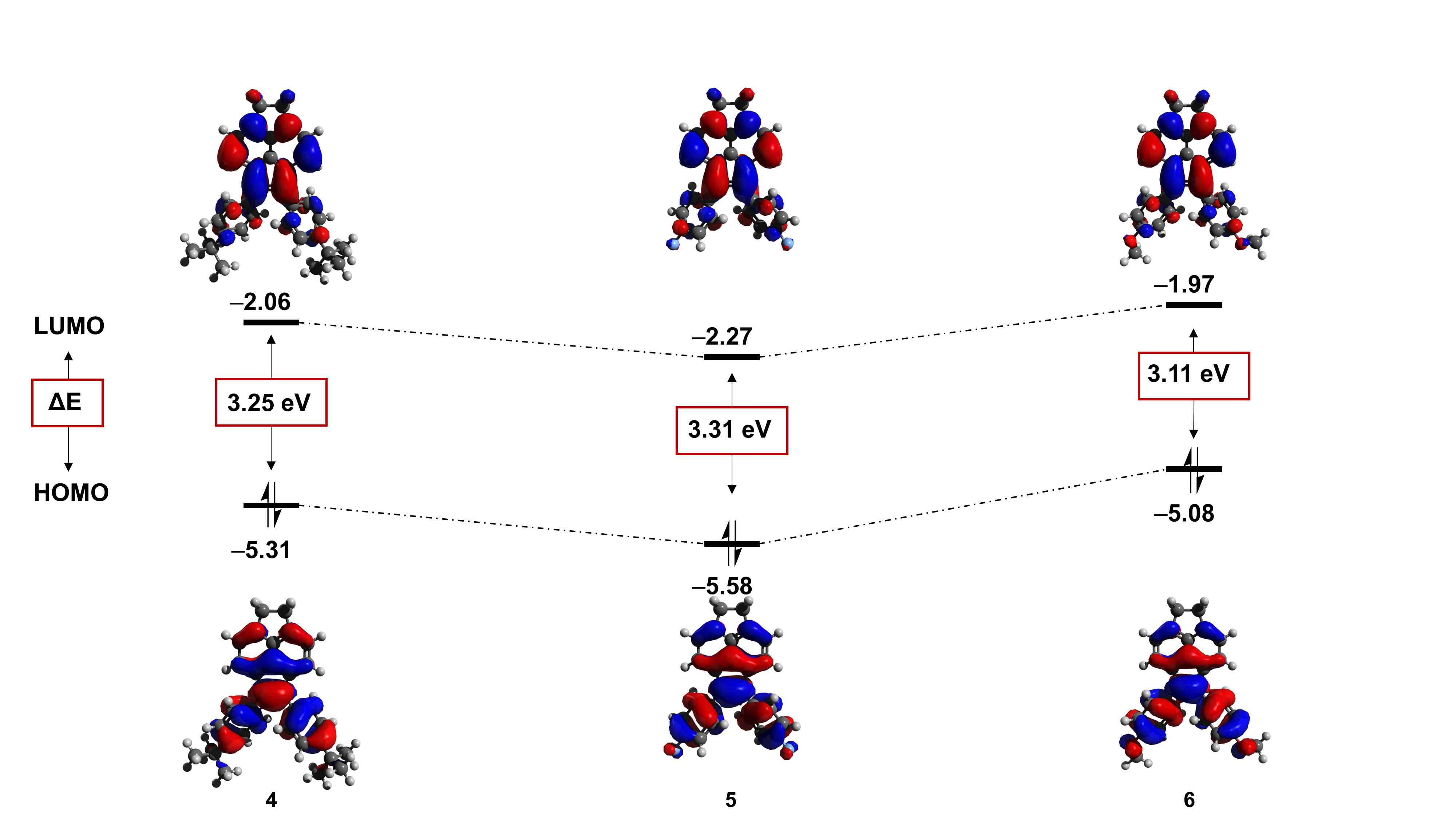


**Figure S85.** Frontier molecular orbitals of the 1,2-dihydropyracylene precursors **4**, **5** and **6** as calculated by DFT at the B3LYP(D3BJ)/6-311G(d,p) level of theory. Iso-surfaces plotted at 0.02 Bohr^–3/2^. Orbital energy levels given in eV.


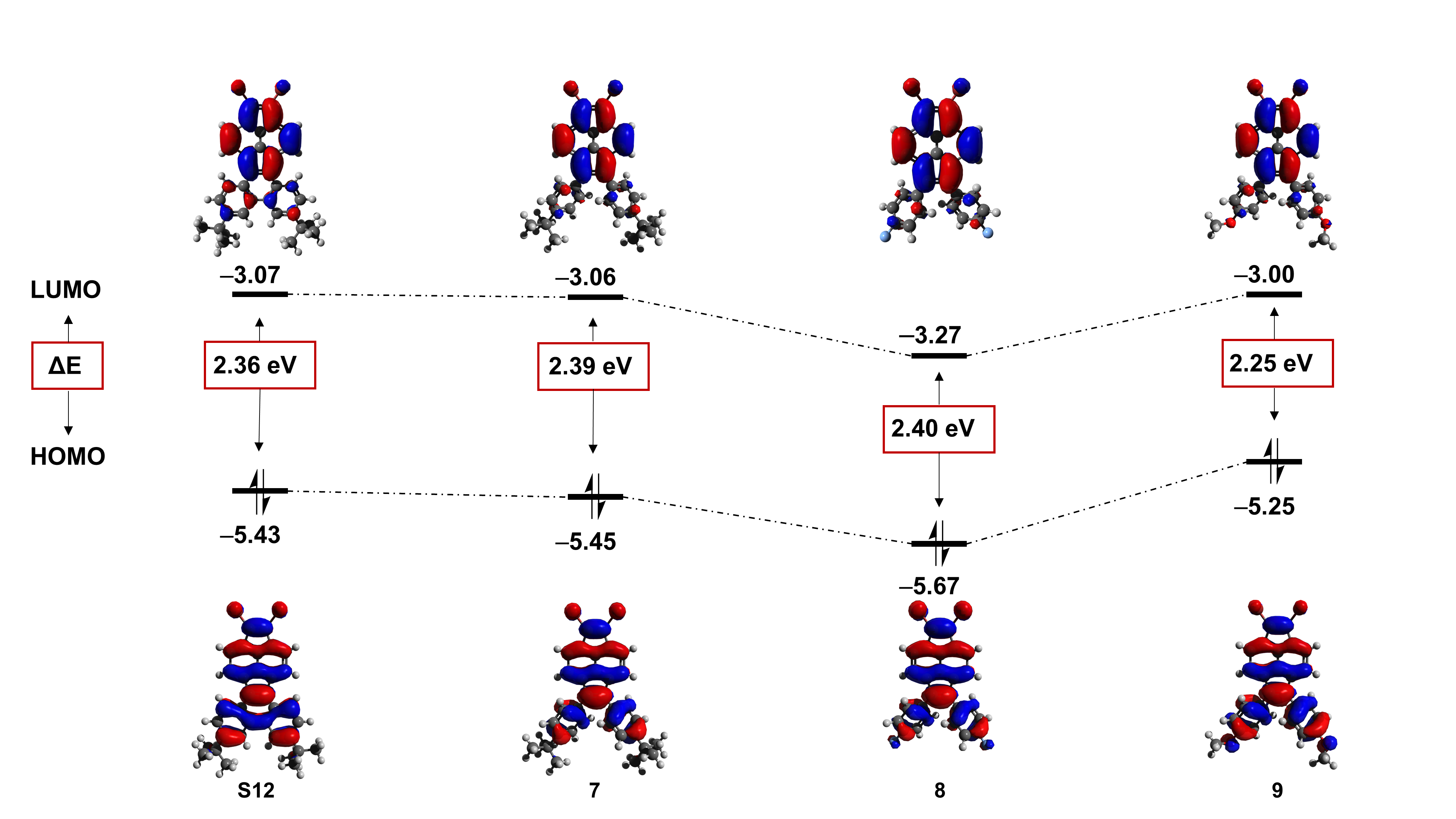


**Figure S86.** Frontier molecular orbitals of the pyracylene precursors **S12**, **7**, **8** and **9** as calculated by DFT at the B3LYP(D3BJ)/6-311G(d,p) level of theory. Iso-surfaces plotted at 0.02 Bohr^–3/2^. Orbital energy levels given in eV.


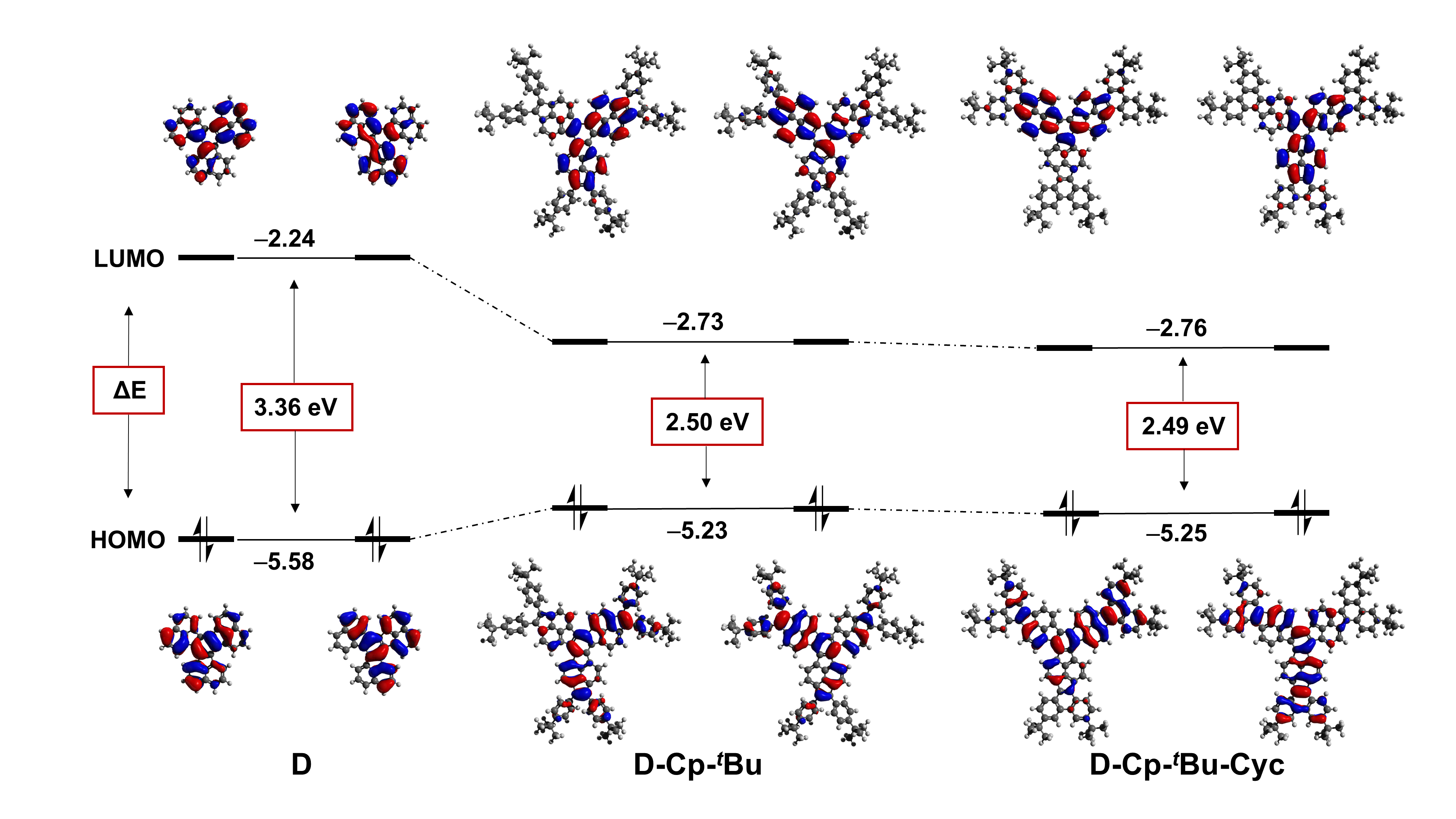


**Figure S87.** Frontier molecular orbitals of **D**, **D‑Cp‑*^t^*Bu** and **D‑Cp‑*^t^*Bu‑Cyc** as calculated by DFT at the B3LYP(D3BJ)/
6-311G(d,p) level of theory. Iso-surfaces plotted at 0.02 Bohr^–3/2^. Orbital energy levels given in eV.


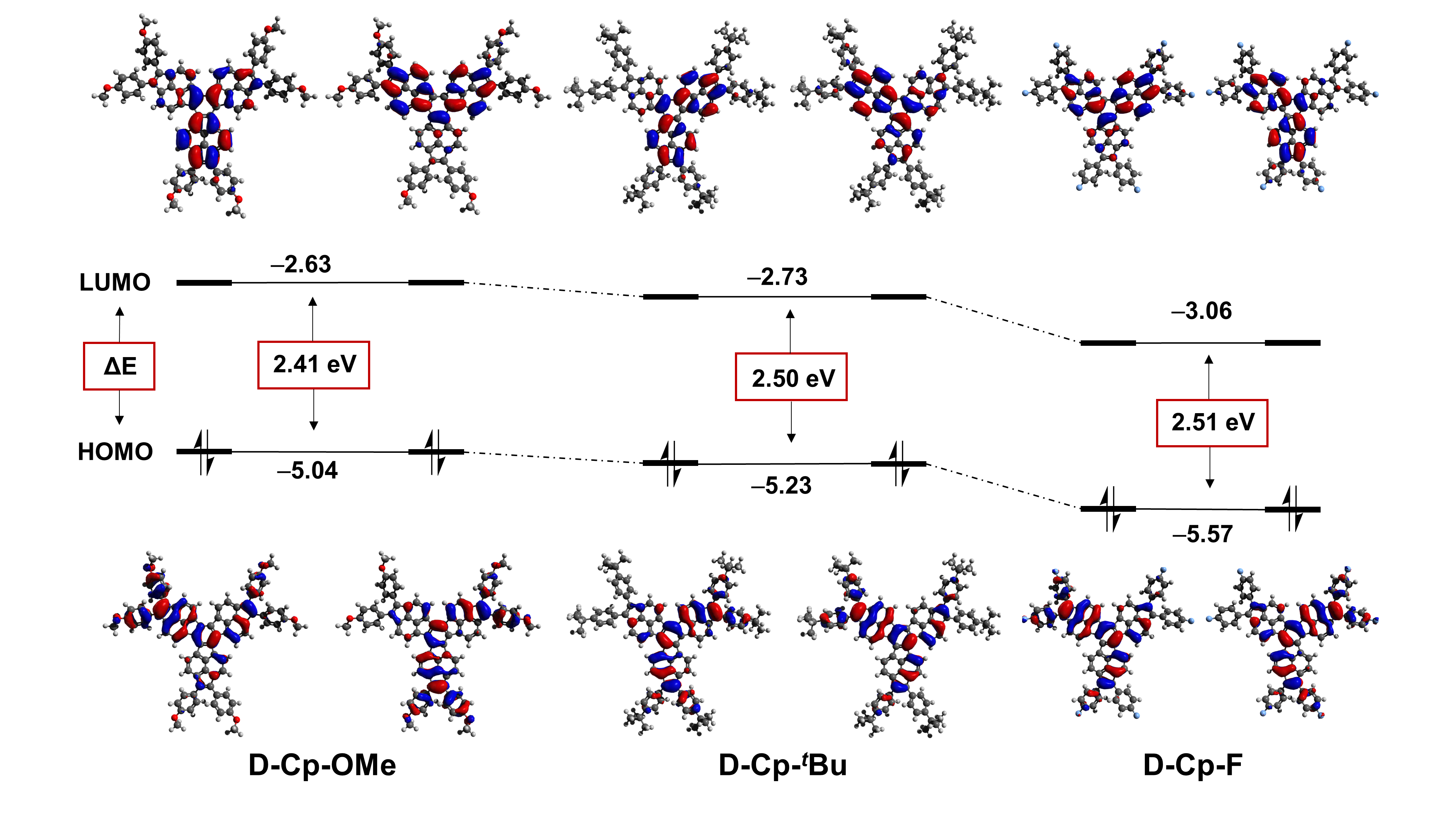


**Figure S88.** Frontier molecular orbitals of **D‑Cp‑*^t^*Bu**, **D‑Cp‑F** and **D‑Cp‑OMe** as calculated by DFT at the B3LYP(D3BJ)/6-311G(d,p) level of theory. Iso-surfaces plotted at 0.02 Bohr^–3/2^. Orbital energy levels given in eV.

**Table S12.** Summary of the computational data and experimental optical gaps for scaffolds **4**–**9** and **S12**.

| PAH | E_HOMO, DFT_[eV] | E_LUMO, DFT_[eV] | E_g, DFT_[eV] | E_g, opt_ [eV]^[a]^ |
| --- | --- | --- | --- | --- |
| **4** | –5.31 | –2.06 | 3.25 | 2.39 |
| **5** | –5.58 | –2.27 | 3.31 | 2.49 |
| **6** | –5.08 | –1.97 | 3.11 | 2.33 |
| **7** | –5.45 | –3.06 | 2.39 | 2.45 |
| **8** | –5.67 | –3.27 | 2.40 | 2.49 |
| **9** | –5.25 | –3.00 | 2.25 | 2.35 |
| **S12** | –5.43 | –3.07 | 2.36 | 2.60 |

[a] Calculated from experimental data, namely by the onset of absorption using the equation E_g,opt_ = (hc) /*λ*_on_.

**Table S13.** Summary of the computational data and experimental optical gaps for cyclotrimers **D**, **D‑Cp‑*^t^*Bu‑Cyc**, **D‑Cp‑*^t^*Bu**, **D‑Cp‑F** and **D‑Cp‑OMe**

| PAH | E_HOMO, DFT_[eV] | E_LUMO, DFT_[eV] | E_g, DFT_ [eV] | E_g, opt_ [eV]^[a]^ |
| --- | --- | --- | --- | --- |
| **D** | –5.32 | –1.96 | 3.35 | 2.65 |
| **D‑Cp‑*^t^*Bu‑Cyc** | –5.25 | –2.76 | 2.49 | 1.80 |
| **D‑Cp‑*^t^*Bu** | –5.23 | –2.73 | 2.50 | 1.87 |
| **D‑Cp‑F** | –5.57 | –3.06 | 2.51 | 1.82 |
| **D‑Cp‑OMe** | –5.04 | –2.63 | 2.40 | 1.82 |

[a] Calculated from experimental data, namely by the onset of absorption using the equation E_g,opt_ = (hc) /*λ*_on_.

1. Theoretical UV/Vis Absorption Data

**Figure S89.** Computed steady-state absorption spectrum of the series of cyclotrimers **D‑Cp‑*^t^*Bu‑Cyc**, **D‑Cp‑*^t^*Bu**, **D‑Cp‑F** and **D‑Cp‑OMe** (TD, CAM-B3LYP(D3BJ)/6-311G(d,p), PCM (CH_2_Cl_2_)). The simulated spectrum was broadened using Gaussian function and full width at half maximum set to 0.20 eV.

**Table S14.** Vertical excitations of **D-Cp-*^t^*Bu** (TD, CAM-B3LYP(D3BJ)/6-311G(d,p), PCM (CH_2_Cl_2_)). Only the contributions with largest linear response coefficients (in parentheses) are shown.

| Excitation | *E* [eV] | * *[nm] | Type | F |
| --- | --- | --- | --- | --- |
| S_1_ | 2.28 | 544 | HOMO → LUMO (0.43) | 0.002 |
| S_2_ | 2.36 | 525 | HOMO → LUMO+2 (0.37) | 0.04 |
| S_3_ | 2.36 | 525 | HOMO–1 → LUMO+2 (0.38) | 0.04 |
| S_4_ | 2.74 | 453 | HOMO–1 → LUMO (0.44) | 0.0 |
| S_5_ | 2.90 | 427 | HOMO–1 → LUMO+2 (0.37) | 2.14 |
| S_6_ | 2.90 | 427 | HOMO → LUMO+2 (0.37) | 2.14 |
| S_7_ | 3.18 | 389 | HOMO–4 → LUMO (0.31) | 0.002 |
| S_8_ | 3.36 | 369 | HOMO–2 → LUMO (0.33) | 0.01 |
| S_9_ | 3.36 | 369 | HOMO–2 → LUMO+1 (0.33) | 0.01 |
| S_10_ | 3.66 | 339 | HOMO–2 → LUMO+1 (0.43) | 0.22 |

**Table S15.** Vertical excitations of **D-Cp-OMe** (TD, CAM-B3LYP(D3BJ)/6-311G(d,p), PCM (CH_2_Cl_2_)). Only the contributions with largest linear response coefficients (in parentheses) are shown.

| Excitation | *E* [eV] | * *[nm] | Type | F |
| --- | --- | --- | --- | --- |
| S_1_ | 2.23 | 556 | HOMO → LUMO (0.33) | 0.002 |
| S_2_ | 2.30 | 538 | HOMO → LUMO+2 (0.35) | 0.06 |
| S_3_ | 2.31 | 537 | HOMO–1 → LUMO+2 (0.35) | 0.06 |
| S_4_ | 2.73 | 455 | HOMO–1 → LUMO (0.33) | 0.0 |
| S_5_ | 2.89 | 429 | HOMO–1 → LUMO+2 (0.33) | 2.17 |
| S_6_ | 2.89 | 429 | HOMO → LUMO+2 (0.33) | 2.17 |
| S_7_ | 3.13 | 396 | HOMO–3 → LUMO (0.35) | 0.002 |
| S_8_ | 3.31 | 375 | HOMO–3 → LUMO+2 (0.39) | 0.01 |
| S_9_ | 3.31 | 374 | HOMO–4 → LUMO+2 (0.39) | 0.01 |
| S_10_ | 3.57 | 347 | HOMO–2 → LUMO (0.34) | 0.1 |

**Table S16.** Vertical excitations of **D-Cp-F** (TD, CAM-B3LYP(D3BJ)/6-311G(d,p), PCM (CH_2_Cl_2_)). Only the contributions with largest linear response coefficients (in parentheses) are shown.

| Excitation | *E* [eV] | * *[nm] | Type | F |
| --- | --- | --- | --- | --- |
| S_1_ | 2.30 | 538 | HOMO → LUMO (0.37) | 0.002 |
| S_2_ | 2.39 | 518 | HOMO → LUMO+2 (0.37) | 0.03 |
| S_3_ | 2.39 | 518 | HOMO–1 → LUMO+2 (0.37) | 0.03 |
| S_4_ | 2.74 | 452 | HOMO–1 → LUMO (0.39) | 0.0 |
| S_5_ | 2.91 | 426 | HOMO–1 → LUMO+2 (0.34) | 2.02 |
| S_6_ | 2.91 | 426 | HOMO → LUMO+2 (0.34) | 2.02 |
| S_7_ | 3.20 | 387 | HOMO–4 → LUMO+1 (0.31) | 0.002 |
| S_8_ | 3.37 | 368 | HOMO–4 → LUMO+2 (0.36) | 0.01 |
| S_9_ | 3.37 | 368 | HOMO–3 → LUMO+2 (0.36) | 0.01 |
| S_10_ | 3.69 | 336 | HOMO–2 → LUMO+1 (0.38) | 0.29 |

**Table S17.** Vertical excitations of **D-Cp-^t^Bu-Cyc** (TD, CAM-B3LYP(D3BJ)/6-311G(d,p), PCM (CH_2_Cl_2_)). Only the contributions with largest linear response coefficients (in parentheses) are shown.

| Excitation | *E* [eV] | * *[nm] | Type | F |
| --- | --- | --- | --- | --- |
| S_1_ | 2.33 | 533 | HOMO → LUMO (0.33) | 0.001 |
| S_2_ | 2.41 | 513 | HOMO → LUMO+2 (0.36) | 0.02 |
| S_3_ | 2.41 | 513 | HOMO–1 → LUMO+2 (0.36) | 0.02 |
| S_4_ | 2.75 | 451 | HOMO–1 → LUMO (0.33) | 0.0 |
| S_5_ | 2.86 | 433 | HOMO → LUMO+2 (0.27) | 2.46 |
| S_6_ | 2.86 | 433 | HOMO–1 → LUMO+2 (0.27) | 2.46 |
| S_7_ | 3.18 | 390 | HOMO–3 → LUMO (0.35) | 0.002 |
| S_8_ | 3.35 | 369 | HOMO–3 → LUMO+2 (0.36) | 0.01 |
| S_9_ | 3.35 | 369 | HOMO–4 → LUMO+2 (0.36) | 0.01 |
| S_10_ | 3.59 | 346 | HOMO–5 → LUMO+2 (0.38) | 0.0 |

1. Nucleus Independent Chemical Shift (NICS)

**Table S18.** NICS values for the 1,2-dihydropyracylene precursors **4**, **5** and **6** calculated at B3LYP/6-311G(d,p) level of theory. The calculated NICS(1)_av_ values are visualized as colored dots, whose size was chosen relative to the largest computed value of the series. NICS values indicating a diatropic ring current are highlighted in blue, NICS values indicating a paratropic ring current are highlighted in red.


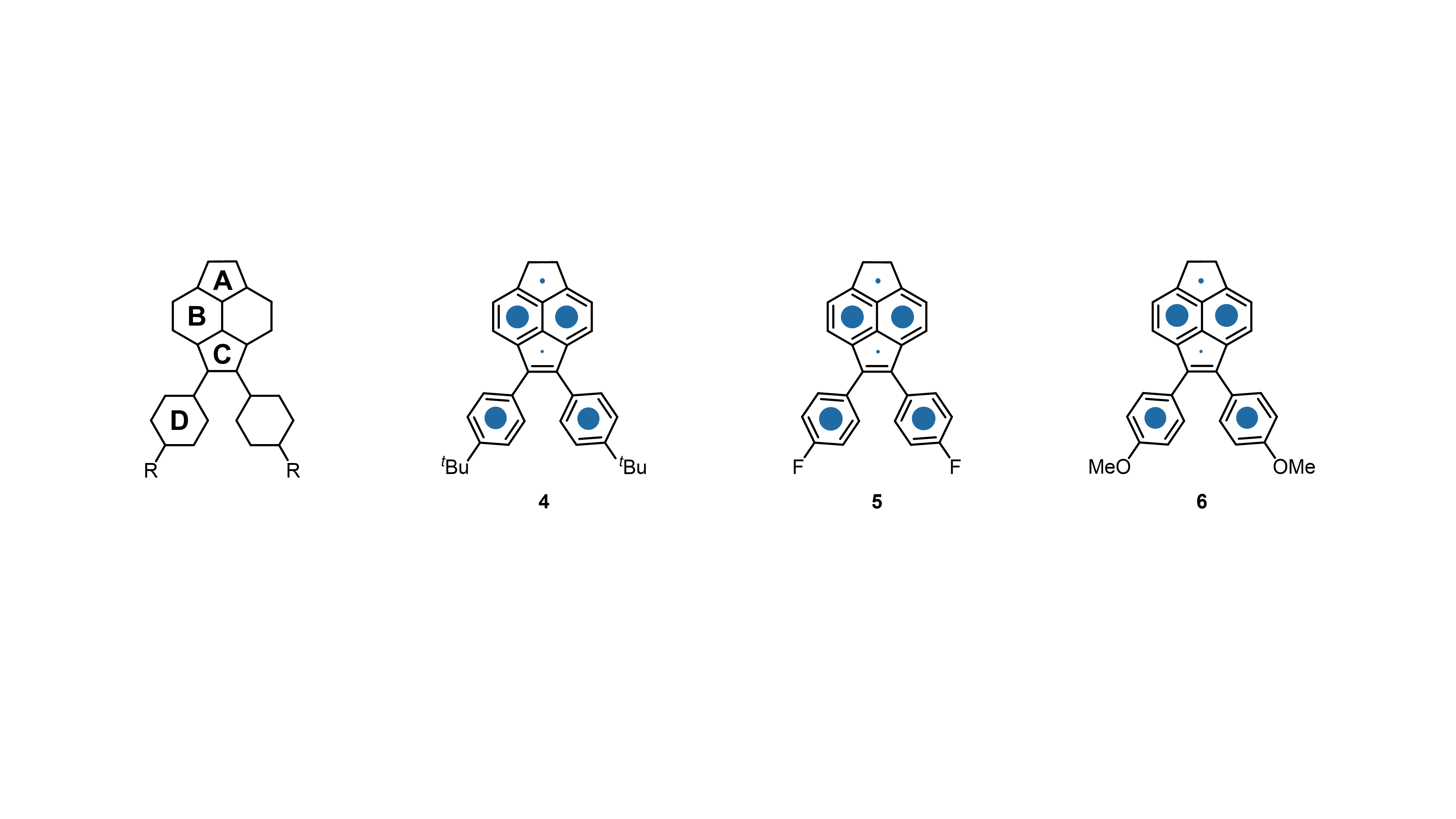


| **PAH** | **Ring A** | **Ring B** | **Ring C** | **Ring D** |
| --- | --- | --- | --- | --- |
| **4**  NICS(+1)  NICS(0)  NICS(–1)  NICS(1)_av_  **5**  NICS(+1)  NICS(0)  NICS(–1)  NICS(1)_av_  **6**  NICS(+1)  NICS(0)  NICS(–1)  NICS(1)_av)_ | –2.16  +0.79  –2.16  –2.16  –2.17  +0.81  –2.17  –2.17  –2.18  +0.76  –2.18  –2.18 | –9.71  –7.29  –9.71  –9.71  –9.76  –7.32  –9.76  –9.76  –9.72  –7.31  –9.72  –9.72 | –1.36  +2.66  –1.36  –1.36  –1.56  +2.36  –1.56  –1.56  –1.29  +2.76  –1.29  –1.29 | –9.57  –7.61  –9.85  –9.71  –10.0  –9.28  –9.73  –9.88  –9.31  –8.38  –9.63  –9.47 |

**Table S19.** NICS values for the pyracylene precursors **S12**, **7**, **8** and **9** calculated at B3LYP/6-311G(d,p) level of theory. The calculated NICS(1)_av_ values are visualized as colored dots, whose size was chosen relative to the largest computed value of the series. NICS values indicating a diatropic ring current are highlighted in blue, NICS values indicating a paratropic ring current are highlighted in red.


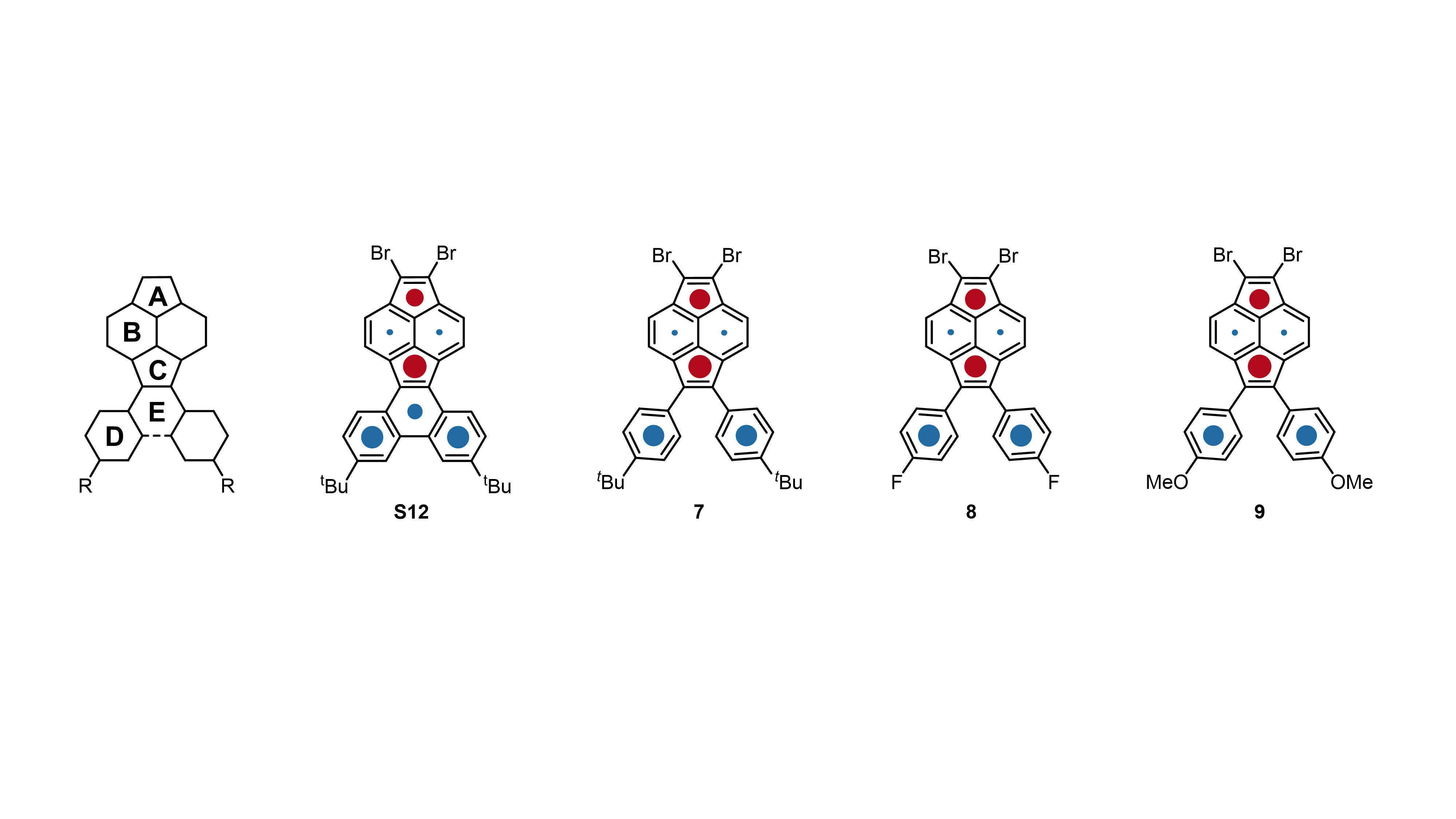


| **PAH** | **Ring A** | **Ring B** | **Ring C** | **Ring D** | **Ring E** |
| --- | --- | --- | --- | --- | --- |
| **S12**  NICS(+1)  NICS(0)  NICS(–1)  NICS(1)_av_  **7**  NICS(+1)  NICS(0)  NICS(–1)  NICS(1)_av_  **8**  NICS(+1)  NICS(0)  NICS(–1)  NICS(1)_av_  **9**  NICS(+1)  NICS(0)  NICS(–1)  NICS(1)_av_ | +8.30  +13.6  +8.30  +8.30  +9.46  +14.6  +9.46  +9.46  +9.56  +15.2  +9.56  +9.56  +9.34  +14.8  +9.34  +9.34 | –2.89  +0.12  –2.89  –2.89  –2.87  +0.24  –2.87  –2.87  –2.98  +0.12  –2.98  –2.98  –2.81  +0.30  –2.81  –2.81 | +10.9  +17.5  +10.9  +10.9  +10.7  +16.8  +10.7  +10.7  +10.2  +16.3  +10.2  +10.2  +10.9  +17.1  +10.9  +10.9 | –10.3  –7.93  –10.3  –10.3  –9.91  –8.03  –10.3  –10.1  –10.1  –9.71  –10.4  –10.3  –9.61  –8.76  –10.0  –9.81 | –7.04  –4.14  –7.04  –7.04 |

**Table S20.** NICS values for **D‑Cp‑*^t^*Bu‑Cyc**, **D‑Cp‑*^t^*Bu**, **D‑Cp‑F** and **D‑Cp‑OMe** calculated at B3LYP/6-311G(d,p) level of theory. The calculated NICS(1)_av_ values are visualized as colored dots, whose size was chosen relative to the largest computed value of the series. NICS values indicating a diatropic ring current are highlighted in blue, NICS values indicating a paratropic ring current are highlighted in red.


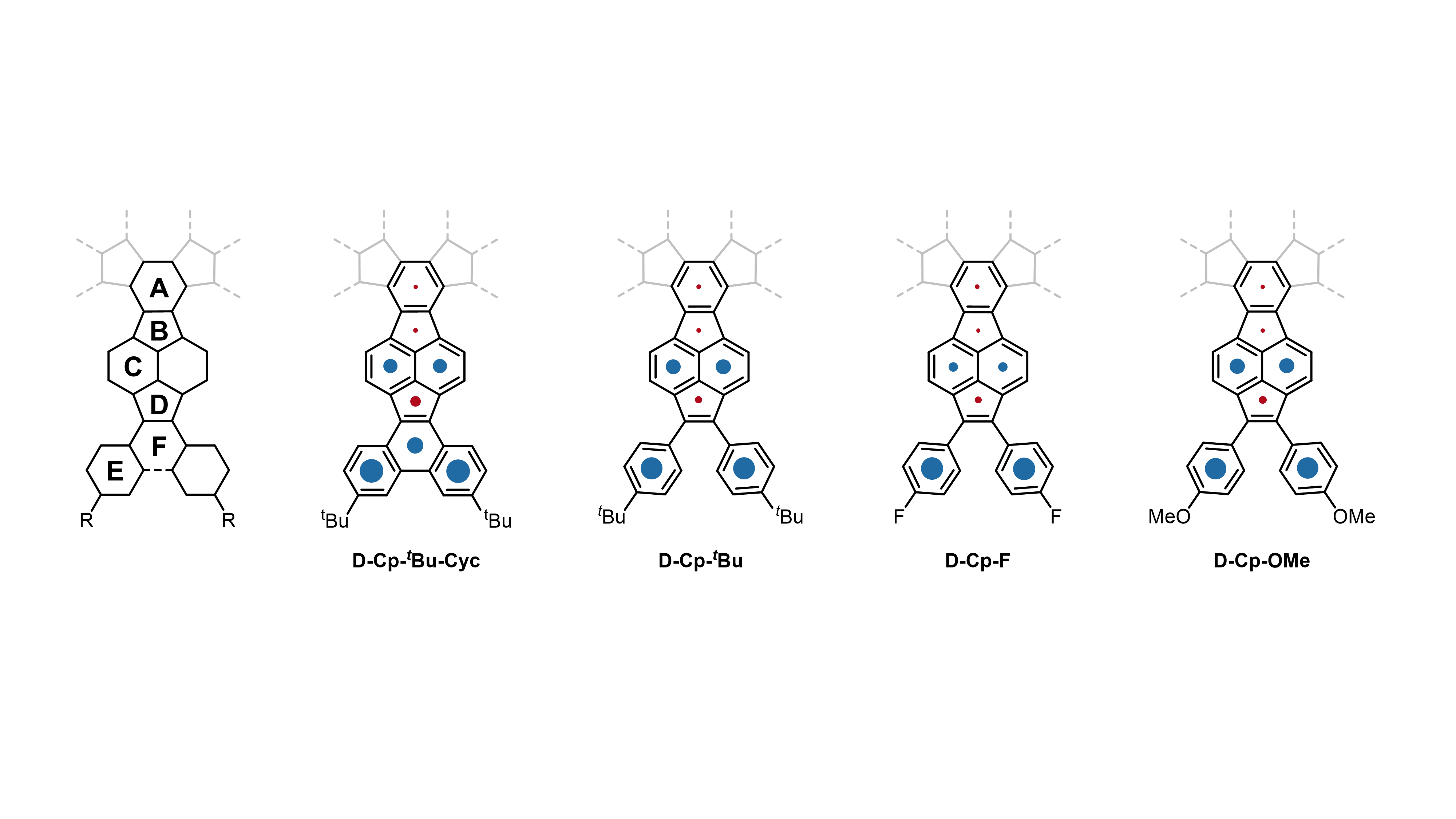


| **PAH** | **Ring A** | **Ring B** | **Ring C** | **Ring D** | **Ring E** | **Ring F** |
| --- | --- | --- | --- | --- | --- | --- |
| **D‑Cp‑*^t^*Bu‑Cyc**  NICS(+1)  NICS(0)  NICS(–1)  NICS(1)_av_  **D‑Cp‑*^t^*Bu**  NICS(+1)  NICS(0)  NICS(–1)  NICS(1)_av_  **D-Cp-F**  NICS(+1)  NICS(0)  NICS(–1)  NICS(1)_av_  **D‑Cp‑OMe**  NICS(+1)  NICS(0)  NICS(–1)  NICS(1)_av_ | +1.40  +4.50  +1.40  +1.40  +2.00  +5.18  +2.00  +2.00  +2.02  +5.20  +2.02  +2.02  +1.92  +5.08  +1.92  +1.92 | +2.15  +7.63  +2.13  +2.14  +1.97  +7.50  +2.06  +2.01  +1.96  +7.49  +1.96  +1.96  +1.98  +7.41  +1.98  +1.98 | –6.17  –3.57  –6.32  –6.24  –6.72  –4.09  –6.92  –6.82  –6.76  –4.14  –6.99  –6.87  –6.70  –4.08  –6.93  –6.81 | +4.66  +10.27  +4.70  +4.68  +3.22  +8.15  +3.22  +3.22  +3.06  +7.92  +3.06  +3.06  +3.32  +8.28  +3.32  +3.32 | –10.35  –7.98  –10.35  –10.35  –9.62  –7.65  –9.87  –9.74  –9.77  –9.33  –10.1  –9.93  –9.36  –8.40  –9.65  –9.50 | –7.18  –4.70  –7.20  –7.19 |

**Table S21.** NICS values for decacyclene **D** and pyracylene **Py** calculated at B3LYP/6-311G(d,p) level of theory. The calculated NICS(1)_av_ values are visualized as colored dots, whose size was chosen relative to the largest computed value of the series. NICS values indicating a diatropic ring current are highlighted in blue, NICS values indicating a paratropic ring current are highlighted in red.


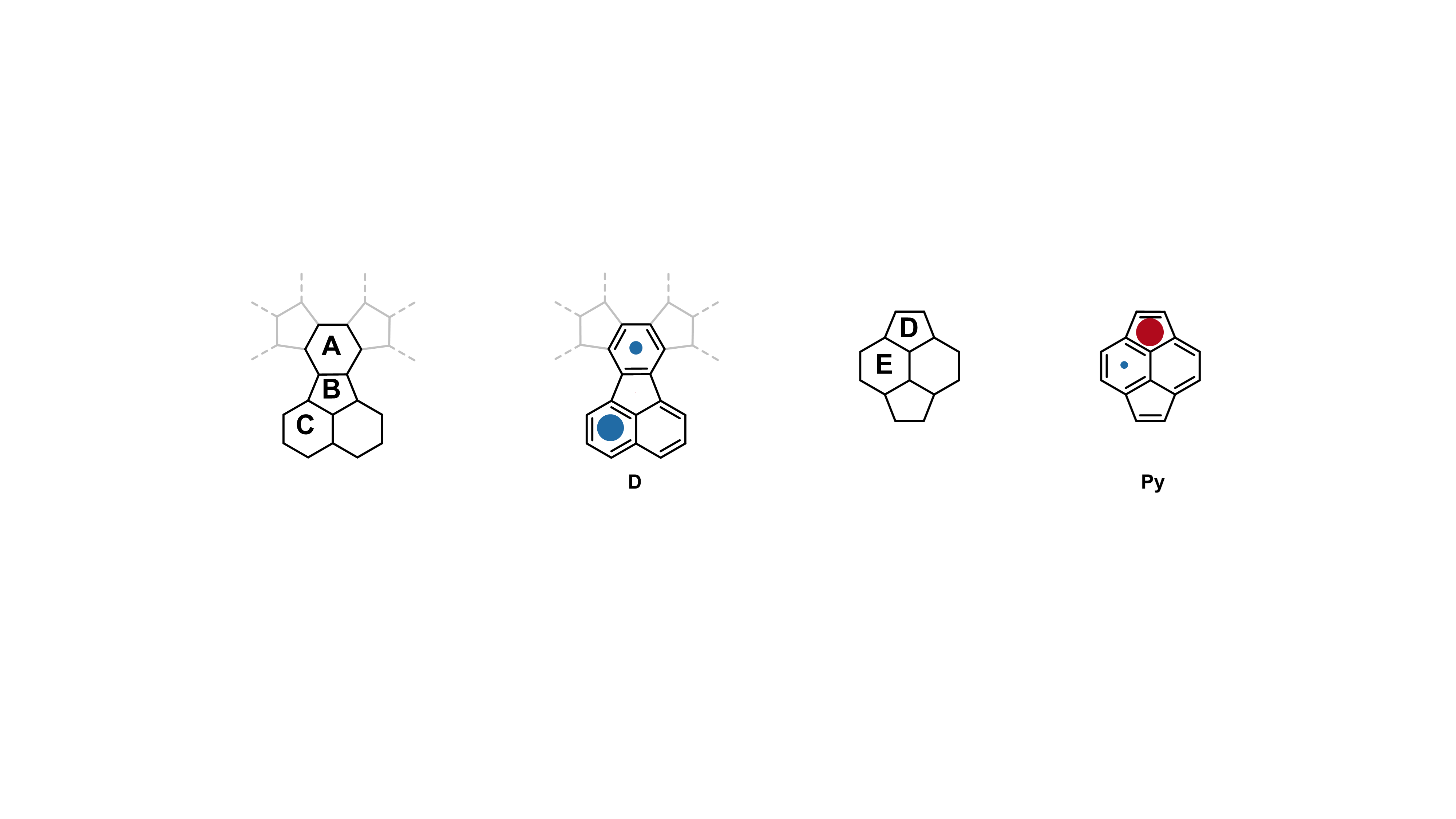


| **PAH** | **Ring A** | **Ring B** | **Ring C** | **Ring D** | **Ring E** |
| --- | --- | --- | --- | --- | --- |
| **D**  NICS(+1)  NICS(0)  NICS(–1)  NICS(1)_av_  **Py**  NICS(+1)  NICS(0)  NICS(–1)  NICS(1)_av_ | –4.80  –2.56  –4.80  –4.80 | +0.33  +5.32  +0.35  +0.34 | –9.73  –7.16  –9.37  –9.55 | +10.2  +16.7  +10.2  +10.2 | –2.81  +0.49  –2.81  –2.81 |

1. Anisotropy of Induced Current Density (ACID)

**Figure S90.** ACID plots for **D‑Cp‑*^t^*Bu**. Contributions from only -orbitals (left) (isovalue 0.02 a.u.) and from all orbitals (right) (isovalue 0.04 a.u.). The magnetic field points out of the paper plane.

**Figure S91.** ACID plots for **D‑Cp‑**^t^**Bu‑Cyc**. Contributions from only -orbitals (left) (isovalue 0.02 a.u.) and from all orbitals (right) (isovalue 0.04 a.u.). The magnetic field points out of the paper plane.

**Figure S92.** ACID plots for **D**. Contributions from only -orbitals (left) (isovalue 0.02 a.u.) and from all orbitals (right) (isovalue 0.04 a.u). The magnetic field points out of the paper plane.

**Figure S93.** ACID plots for **Py**. Contributions from only -orbitals (left) (isovalue 0.02 a.u.) and from all orbitals (right) (isovalue 0.04 a.u). The magnetic field points out of the paper plane.

1. Harmonic Oscillator Model of Aromaticity (HOMA)


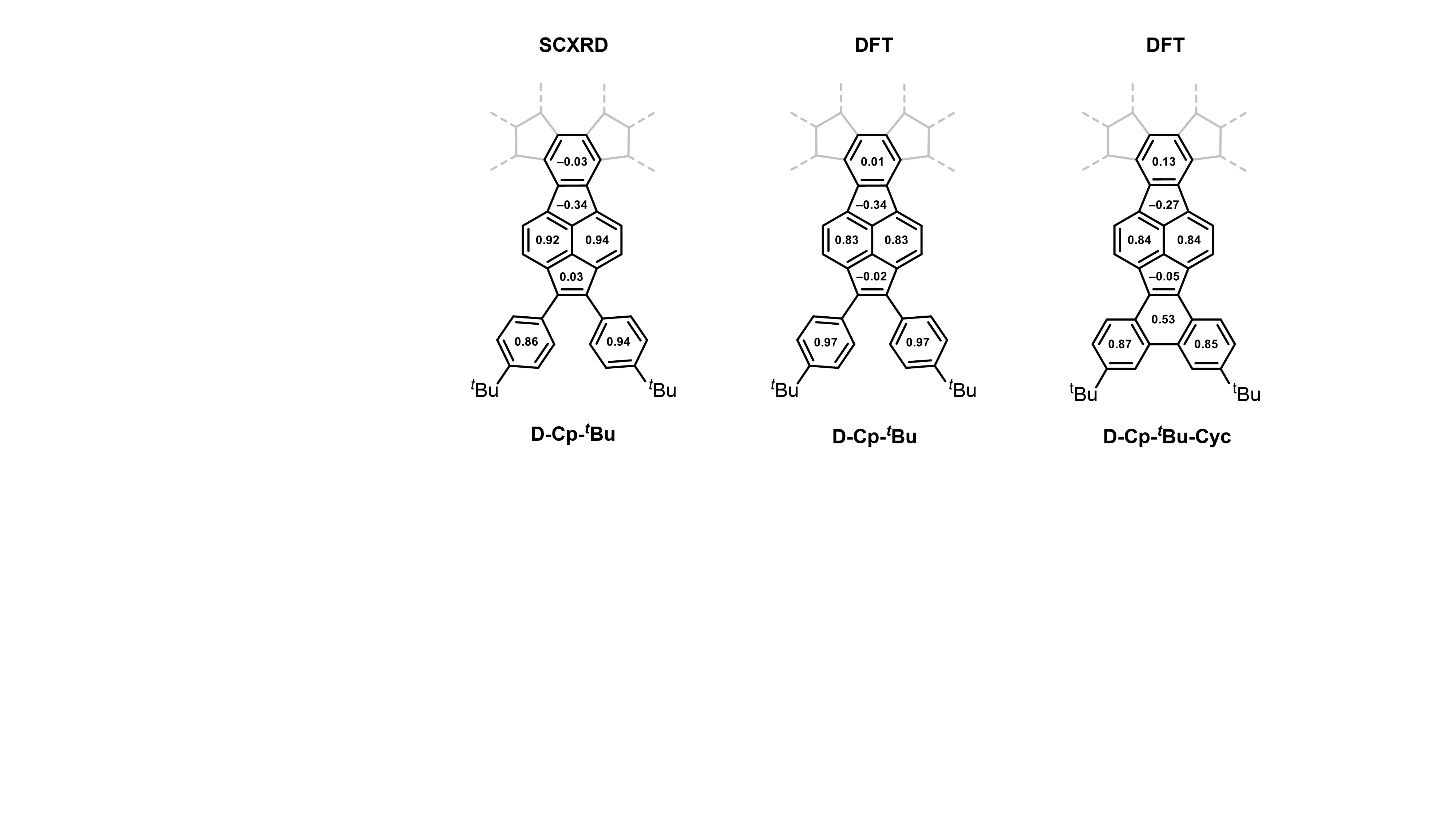


**Figure S94.** HOMA values for **D‑Cp‑*^t^*Bu** calculated from the X-ray crystal structure (left) and the DFT‑optimized structure (middle). HOMA values for **D‑Cp‑*^t^*Bu‑Cyc** calculated from the DFT‑optimized structure (right).

1. References

[1] G. R. Fulmer, A. J. M. Miller, N. H. Sherden, H. E. Gottlieb, A. Nudelman, B. M. Stoltz, J. E. Bercaw, K. I. Goldberg, *Organometallics* **2010**, *29*, 2176.

[2] G. M. Sheldrick, *Acta Crystallogr., Sect. A: Found. Crystallogr.* **2008**, *64*, 112.

[3] G. M. Sheldrick, *Acta Crystallogr., Sect. C: Cryst. Struct. Commun.* **2015**, *71*, 3.

[4] C. F. Macrae, P. R. Edgington, P. McCabe, E. Pidcock, G. P. Shields, R. Taylor, M. Towler, J. van de Streek, *J. Appl. Crystallogr.* **2006**, *39*, 453.

[5] M. J. Frisch, G. W. Trucks, H. B. Schlegel, G. E. Scuseria, M. A. Robb, J. R. Cheeseman, G. Scalmani, V. Barone, G. A. Petersson, H. Nakatsuji, X. Li, M.Caricato, A. V. Marenich, J. Bloino, B. G. Janesko, R. Gomperts, B. Mennucci, H. P. Hratchian, J. V. Ortiz, A. F. Izmaylov, J. L. Sonnenberg, Williams, F.Ding, F. Lipparini, F. Egidi, J. Goings, B. Peng, A. Petrone, T. Henderson, D. Ranasinghe, V. G. Zakrzewski, J. Gao, N. Rega, G. Zheng, W. Liang, MHada, M. Ehara, K. Toyota, R. Fukuda, J. Hasegawa, M. Ishida, T. Nakajima, Y. Honda, O. Kitao, H. Nakai, T. Vreven, K. Throssell, J. A. Montgomery Jr.,J. E. Peralta, F. Ogliaro, M. J. Bearpark, J. J. Heyd, E. N. Brothers, K. N. Kudin, V. N. Staroverov, T. A. Keith, R. Kobayashi, J. Normand, K. Raghavachari,A. P. Rendell, J. C. Burant, S. S. Iyengar, J. Tomasi, M. Cossi, J. M. Millam, M. Klene, C. Adamo, R. Cammi, J. W. Ochterski, R. L. Martin, K. Morokuma,O. Farkas, J. B. Foresman, D. J. Fox, *Gaussian 16, Rev. C. 01*, Wallingford, CT, **2016**.

[6] a) P. J. Stephens, F. J. Devlin, C. F. Chabalowski, M. J. Frisch, *J. Phys. Chem.* **1994**, *98*, 11623; b) A. D. Becke, *J. Chem. Phys.* **1993**, *98*, 5648; c) Lee, Yang, Parr, *Phys. Rev., B Condens.* **1988**, *37*, 785; d) S. H. Vosko, L. Wilk, M. Nusair, *Can. J. Phys.* **1980**, *58*, 1200.

[7] R. Krishnan, J. S. Binkley, R. Seeger, J. A. Pople, *J. Chem. Phys.* **1980**, *72*, 650.

[8] S. Grimme, J. Antony, S. Ehrlich, H. Krieg, *J. Chem. Phys.* **2010**, *132*, 154104.

[9] S. Grimme, S. Ehrlich, L. Goerigk, *J. Comput. Chem.* **2011**, *32*, 1456.

[10] J. R. Cheeseman, G. W. Trucks, T. A. Keith, M. J. Frisch, *J. Chem. Phys.* **1996**, *104*, 5497.

[11] a) K. Wolinski, J. F. Hinton, P. Pulay, *J. Am. Chem. Soc.* **1990**, *112*, 8251; b) R. Ditchfield, *Mol. Phys* **1974**, *27*, 789; c) R. McWeeny, *Phys. Rev.* **1962**, *126*, 1028; d) F. London, *J. Phys. Radium* **1937**, *8*, 397.

[12] T. Lu, F. Chen, *J. Comput Chem.* **2012**, *33*, 580.

[13] a) T. A. Keith, R. F. Bader, *Chem. Phys. Lett.* **1993**, *210*, 223; b) T. A. Keith, R. Bader, *Chem. Phys. Lett.* **1992**, *194*, 1.

[14] a) D. Geuenich, K. Hess, F. Köhler, R. Herges, *Chem. Rev.* **2005**, *105*, 3758; b) R. Herges, D. Geuenich, *J. Phys. Chem. A* **2001**, *105*, 3214.

[15] py.Aroma: An Intuitive Graphical User Interface for Diverse Aromaticity Analyses. *ChemRxiv*. **2024**; *doi:10.26434/chemrxiv-2024-mjmj8*.

[16] T. Yanai, D. P. Tew, N. C. Handy, *Chem. Phys. Lett.* **2004**, *393*, 51.

[17] a) M. Cossi, N. Rega, G. Scalmani, V. Barone, *J. Comput. Chem.* **2003**, *24*, 669; b) V. Barone, M. Cossi, *J. Phys. Chem. A* **1998**, *102*, 1995.

[18] R. Dennington, T. A. Keith, J. M. Millam, *GaussView Version 6*, **2019**.

[19] J. Bergner, C. Walla, F. Rominger, A. Dreuw, M. Kivala, *Chem. Eur. J.* **2022**, *28*, e202201554.

[20] S. R. Bheemireddy, P. C. Ubaldo, P. W. Rose, A. D. Finke, J. Zhuang, L. Wang, K. N. Plunkett, *Angew. Chem. Int. Ed.* **2015**, *54*, 15762.

[21] A. W. Amick, L. T. Scott, *J. Org. Chem.* **2007**, *72*, 3412.

[22] K. F. Lang, M. Zander, *Chem. Ber.* **1961**, *94*, 1871.

[23] M. J. Mio, L. C. Kopel, J. B. Braun, T. L. Gadzikwa, K. L. Hull, R. G. Brisbois, C. J. Markworth, P. A. Grieco, *Org. Lett.* **2002**, *4*, 3199.

[24] W. An, G. Li, J. Ma, Y. Tian, F. Xu, *Synlett* **2014**, *25*, 1585.

[25] D. M. Ho, R. A. Pascal, *Chem. Mater.* **1993**, *5*, 1358.

[26] B. Freiermuth, S. Gerber, A. Riesen, J. Wirz, M. Zehnder, *J. Am. Chem. Soc.* **1990**, *112*, 738.

[27] J. C. Costa, R. J. Taveira, C. F. Lima, A. Mendes, L. M. Santos, *Opt. Mater* **2016**, *58*, 51.

[28] a) C. M. Cardona, W. Li, A. E. Kaifer, D. Stockdale, G. C. Bazan, *Adv. Mater.* **2011**, *23*, 2367; b) A. J. Bard, L. R. Faulkner, *Electrochemical methods. Fundamentals and applications*, Wiley, New York, Weinheim, 2001; c) W. N. Hansen, G. J. Hansen, *Phys. Rev. A* **1987**, *36*, 1396; d) S. Trasatti, *Pure Appl. Chem.* **1986**, *58*, 955.
